# Voluntary Wheel Running Reverses Age-Induced Changes in Hippocampal Gene Expression

**DOI:** 10.1371/journal.pone.0022654

**Published:** 2011-08-08

**Authors:** Rachel A. Kohman, Sandra L. Rodriguez-Zas, Bruce R. Southey, Keith W. Kelley, Robert Dantzer, Justin S. Rhodes

**Affiliations:** 1 Department of Psychology, Beckman Institute, University of Illinois, Urbana, Illinois, United States of America; 2 Department of Animal Sciences, University of Illinois at Urbana-Champaign, Urbana, Illinois, United States of America; 3 Integrated Immunology and Behavior Program, University of Illinois at Urbana-Champaign, Urbana, Illinois, United States of America; Centre National de la Recherche Scientifique, University of Bordeaux, France

## Abstract

Normal aging alters expression of numerous genes within the brain. Some of these transcription changes likely contribute to age-associated cognitive decline, reduced neural plasticity, and the higher incidence of neuropathology. Identifying factors that modulate brain aging is crucial for improving quality of life. One promising intervention to counteract negative effects of aging is aerobic exercise. Aged subjects that exercise show enhanced cognitive performance and increased hippocampal neurogenesis and synaptic plasticity. Currently, the mechanisms behind the anti-aging effects of exercise are not understood. The present study conducted a microarray on whole hippocampal samples from adult (3.5-month-old) and aged (18-month-old) male BALB/c mice that were individually housed with or without running wheels for 8 weeks. Results showed that aging altered genes related to chromatin remodeling, cell growth, immune activity, and synapse organization compared to adult mice. Exercise was found to modulate many of the genes altered by aging, but in the opposite direction. For example, wheel running increased expression of genes related to cell growth and attenuated expression of genes involved in immune function and chromatin remodeling. Collectively, findings show that even late-onset exercise may attenuate age-related changes in gene expression and identifies possible pathways through which exercise may exert its beneficial effects.

## Introduction

Normal aging is associated with a decline in cognitive function, reduced neural plasticity, and is a primary risk factor for many neurodegenerative diseases including Alzheimer’s disease [1]–[5]. As the number of individuals surviving into old age continues to increase, the need to identify the mediating factors of brain aging and potential treatments to slow or prevent its onset is crucial for ensuring healthy aging and reducing health care costs.

One area of the brain that is particularly vulnerable to age-related alterations is the hippocampus. Aging is associated with a decrease in hippocampal volume as well as functional deficits [1], [2], [6]–[8]. For example, aged animals consistently show impaired performance on hippocampus-dependent cognitive tasks including the water maze, contextual fear conditioning, and the radial arm maze [8]–[10]. Corresponding age-related impairments have been reported in several neurobiological correlates of learning and memory. For instance, aged mice show a reduction in both the production and survival of new neurons in the hippocampus [2], [5], [11]. Further, aged animals show an impaired ability to sustain long-term potentiation as well as reduced density of dendritic spines [3], [12]. Analysis of hippocampal gene expression indicates that these age-related functional deficits may result from increased expression of genes related to oxidative stress and inflammation and decreased expression of genes involved in mitochondrial function and neural plasticity [13], [14]. Clearly, the hippocampus undergoes substantial age-related changes and is a candidate brain region to investigate mechanisms of brain aging.

Though aging is inevitable, the age-related decline in neural function is not. Lifestyle interventions, such as environmental enrichment, aerobic exercise, and caloric restriction, are reported to have preventative and possibly restorative effects in aged individuals [2], [4], [6], [15], [16]. Exercise is one potent intervention well known to enhance performance on a number of cognitive tasks in both humans and animals, including those that involve the hippocampus [2], [6], [17]–[19]. For example, van Praag et al. [2] reported that wheel running enhanced spatial learning in aged mice compared to sedentary mice. In addition to its cognitive benefits, exercise improves cardiovascular health and reduces the risk of several age-related pathologies, such as metabolic syndrome, diabetes, Alzheimer’s and Parkinson’s disease [20]–[22]. The cognitive and health benefits of exercise are associated with several physiological and anatomical changes, many of which take place in the hippocampus, such as increased production of growth factors, increased efficiency of the cerebral vascular system, enhanced hippocampal neurogenesis, and regulation of the immune and endocrine systems [23]–[27]. While these changes likely contribute, the precise mechanisms behind the anti-aging effects of exercise are not completely understood.

One approach to identify pathways that are altered by exercise in aged subjects is to assess changes in gene expression using microarray technology. A study by Stranahan et al. [28] evaluated hippocampal gene expression of aged mice that had lifelong access to running wheels compared to aged mice housed under sedentary conditions. They report that wheel running reduced expression of genes related to oxidative stress and apoptosis and increased expression of genes related to protein trafficking, glutamatergic neurotransmission, and the stress response. These data reveal that engaging in lifelong exercise may be protective in some respects. However, the ability of shorter periods of exercise, beginning late in life, to modulate global transcription has yet to be determined.

The present study evaluated age-related changes in hippocampal gene expression and assessed whether aerobic exercise differentially modulates gene expression in adult and aged mice. While prior reports have evaluated the impact of exercise on hippocampal gene expression in aged *or* adult subjects [28]–[30], the current study is unique in that it compares the effects of late onset exercise in both adult and aged mice. This approach is advantageous in that it allowed for identification of genes/networks of genes that are differentially regulated in adult and aged subjects in response to exercise as well as revealed age-induced changes in the hippocampus that are reversed by wheel running. Ultimately, these data have the potential to facilitate development of efficacious treatments that will help reduce the growing problem of age-related cognitive decline and neuropathologies.

## Methods

### 2.1. Experimental subjects

Subjects were 12 adult (3.5-month-old) and 8 aged (18-month-old) male BALB/c mice from an in-house aging colony. Mice were given *ad libitum* access to food and water and housed under a 12 hr light/dark cycle in the AAALAC approved Edward R. Madigan Laboratory Animal Facility. Prior to the start of the experiment mice were group housed (2–3 mice per cage), but during the experiment all mice were individually housed. Animals were treated in compliance with the *Guide for the Care and Use of Laboratory Animals* and the experiment was conducted in accordance with a protocol approved by the Institutional Animal Care and Use Committee (IACUC) at the University of Illinois at Urbana-Champaign (protocol number 09167 and Animal Welfare Assurance Number A3118-01).

### 2.2. Experimental design

Mice were divided by age into either the exercise (access to a running wheel) or sedentary condition, for a total of four treatment groups (adult exercise n = 6, adult sedentary = 6, aged exercise n = 4, aged sedentary n = 4). Mice in the sedentary condition were individually housed in standard polypropylene shoebox cages (29 cm L×19 cm W×13 cm H). Mice in the exercise condition were individually housed in cages (36 cm L×20 cm W×14 cm H) with a 23 cm diameter running wheel (Respironics, Bend, OR). Throughout the 8 weeks wheel rotations were continuously collected in 1 min intervals via magnetic switches interfaced to a computer using the VitalView software (Respironics, Bend, OR). Sedentary mice were deliberately not housed in cages with locked wheels since mice climb in locked wheels [31]–[33] and we wanted to limit physical activity in the sedentary group. Animals were weighted weekly, throughout the experiment.

### 2.3. Tissue collection and RNA isolation

Animals were sacrificed by rapid decapitation without anesthesia in the early afternoon 8 weeks after individually housing with or without a running wheel. The whole hippocampus was dissected on a chilled glass dish and immediately placed into RNAlater solution (Qiagen, Valencia, CA) and stored at −20°C until RNA isolation. Hippocampal samples were homogenized and RNA purified (RNeasy Mini kit, Qiagen, Valencia, CA), then quantified and assessed for purity using a NanoDrop ND-1000 Spectrophotometer (NanoDrop Technologies, Wilmington, DE) and an Agilent 2100 bioanalyzer (Agilent Technologies, Santa Clara CA).

### 2.4. Illumina microarray

For Illumina microarray analysis, samples were prepared and analyzed by the W. M. Keck Center for Comparative and Functional Genomics in the Roy J. Carver Biotechnology Center at the University of Illinois at Urbana-Champaign. RNA quality was determined using the Agilent 2100 Bioanalyzer (Agilent Technologies, Palo Alto, CA). 300 ng of high quality total RNA were primed with an oligo(dT) primer bearing a T7 promoter, and reverse transcribed in the first-strand cDNA synthesis using the Illumina TotalPrep RNA Amplification Kit (Ambion, Inc., Austin, TX). Single stranded cDNA was then converted into double stranded cDNA according to the manufacturer’s instructions. The double-stranded cDNA was purified and served as a template in the 14-hour *in vitro* transcription (IVT) reaction. Prior to hybrifdization, the synthesized biotin labeled cRNA was cleaned up using the same Amplification Kit. After the quality control assessment, 1.5 µg of cRNA from each experimental sample along with hybridization controls were hybridized for 16 hours to the MouseWG-6 v2 Expression BeadChips (Illumina, Inc., San Diego, CA) in the 58°C Illumina Hybridization Oven. Washing, staining with streptavidin-Cy3 (GE Healthcare Bio-Sciences, Piscataway, NJ), and scanning was performed according to the Illumina Whole-Genome Gene Expression Direct Hybridization Assay Guide (revision A). Four BeadChips were used in this study, each containing six arrays. The arrays were scanned using an Illumina BeadArray Reader. Each array image was visually screened to discount for signal artifacts, scratches or debris. The images were analyzed using the GeneExpression Analysis Module (version 1.6.0) of the Illumina GenomeStudio software.

### 2.5. qRT-PCR

To confirm microarray results, five genes (see Table 1) that showed significant changes in expression from exercise and/or age were selected from the microarray results. The amount of specific mRNA transcript present in each hippocampal sample was determined by two-step quantitative real-time reverse-transcription polymerase chain reaction (qRT-PCR). Each sample was run in triplicate for each gene. The amount of mRNA present was determined by utilizing TaqMan™ probe and primer chemistry (Applied Biosystems, Foster City, CA) specifically designed to bind to reverse-transcribed cDNA of the genes of interest using an Applied Biosystems 7900HT PCR instrument (Applied Biosystems, Foster City, CA). Florescence data (ΔRn) was exported from the Applied Biosystems SDS software and analyzed by DART (Data Analysis for RT-PCR) [34]. Gene expression data were normalized by dividing the Ro values of the target genes by the Ro values of the endogenous control gene 18S.

**Table 1:** Genes selected for RT-PCR.

*Gene name*	*Assay ID*
Brain derived neurotrophic factor	Mm01334047_m1
Beta-2 microglobulin	Mm00437762_m1
Glutathione peroxidase 8 (putative)	Mm01297261_m1
Complement component 4B	Mm00437893_g1
Histocompatibility 2, D region locus 1	Mm04208019_gH
18S (endogenous control)	Hs99999901_s1

### 2.6. Statistical analysis

RT-PCR gene expression data were analyzed by two-way analysis of variance (ANOVA) with Age and Exercise condition as the between-subjects variables. Body weight and distance ran was analyzed by repeated measures ANOVA with Age and Exercise condition as the between-subjects variables and Day as the within-subjects (i.e., repeated-measure) variable. An alpha level of *p*<0.05 was considered statistically significant.

### 2.7. Microarray data analysis

Probe-level bead summary data were provided by the Illumina Bead Studio toolkit. The intensities were normalized using the Bioconductor lumi R package [35]. Transformation included background correction, variance stabilizing transformation and quantile normalization method [36]. The normalized values were analyzed using the Bioconductor R/manova package and the PROC MIXED procedure in SAS/STAT Version 9.2 (SAS Institute Inc., Cary, NC, USA). The mixed effects model used to describe the normalized expression measurements included the main fixed effects of age and exercise, the interaction between age and exercise and the random effect of Bead array. A False Discovery Rate multiple-test adjustment was implemented on the P-values resulting from the analysis [37], only genes significant at p<0.005 were included in the analysis.

The raw and normalized microarray data were deposited into the Gene Expression Omnibus (GEO) public database in compliance with Minimum Information About a Microarray Experiment (MIAME) guidelines (Accession number is GSE29075).

### 2.8. Functional class analysis by gene ontology

To identify functionally related categories of genes, significant genes from the main effects of Age and Exercise were individually imported into DAVID Bioinformatics database [38], [39] and clustered into biological process gene ontology (GO) terms. Enriched functional categories were calculated by a Fisher Exact test. Only categories that consisted of 3 or more genes and had an FDR of less than 5% were included in the final list of overrepresented GO categories.

## Results

### 3.1. Body weight

**Figure 1:**
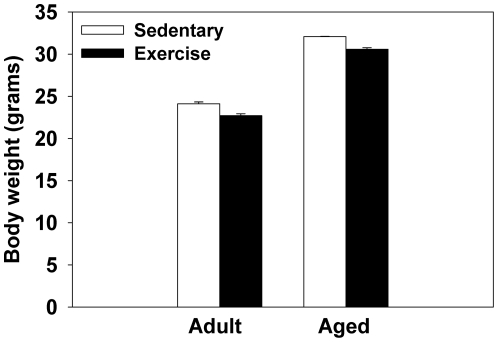
Average body weight (grams) of adult and aged mice. Means ± standard error of the mean (SEM).

As expected, aged mice weighted more than adult mice (F(1,16) = 107.7;p<0.0001, see Figure 1). There was also a significant main effect of Exercise condition (F(1,16) = 6.46;p<0.05; see Figure 1), as mice with access to running wheels weighed less than sedentary mice.

### 3.2. Wheel running data

**Figure 2:**
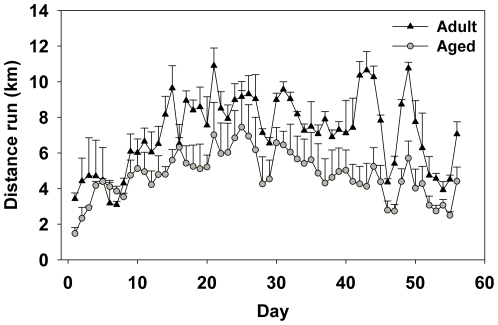
Average distance (km) ran per day by aged and adult mice over eight weeks of running wheel access. Means ± SEM.

There was a significant main effect of Day for distance (km) run per day (F(55,220) = 5.93;p<0.0001). Inspection of Figure 2 shows that distance run tended to increase daily across both age groups the first month and then decline daily the second month. Overall aged mice ran significantly less than adult mice as show by a significant main effect of Age (F(1,4) = 12.7;p<0.05; see Figure 2). Aged mice ran an average of 4.75 km/day and adult mice ran an average of 7.20 km/day.

### 3.3. Microarray results

#### 3.3.1. Age-related changes in hippocampal gene expression

A total of 1,193 genes were differentially expressed in adult and aged mice (596 upregulated and 597 downregulated; see Table S2). GO analysis revealed that aged mice showed significant enrichment of categories related to chromatin remodeling, antigen presentation, cell cycle, response to hormones, organization of synapses, and lipid metabolism (EASE score p<0.002 and FDR<5%; see Table 2). The top five gene ontologies, starting with the most enriched, are “cellular macromolecular complex subunit organization, DNA packaging, antigen processing and presentation of peptide antigen, macromolecular complex subunit organization, and chromosome organization” indicating that aged mice show numerous alterations in chromatin organization as well as some changes in immune activity compared to adult mice.

**Table 2:** Age-induced changes.

GO term	Gene count	P value	FDR
***Chromatin remodeling***			
GO:0051276∼chromosome organization	43	0.0001	0.25
GO:0006325∼chromatin organization	35	0.0003	0.52
GO:0006333∼chromatin assembly or disassembly	17	0.0003	0.59
GO:0006323∼DNA packaging	17	0.0001	0.20
GO:0031497∼chromatin assembly	14	0.0002	0.30
GO:0043933∼macromolecular complex subunit organization	40	0.0001	0.20
GO:0065003∼macromolecular complex assembly	35	0.0008	1.38
GO:0034621∼cellular macromolecular complex subunit organization	30	0.0001	0.18
GO:0034622∼cellular macromolecular complex assembly	26	0.0004	0.72
GO:0065004∼protein-DNA complex assembly	14	0.0002	0.35
GO:0034728∼nucleosome organization	14	0.0002	0.35
GO:0006334∼nucleosome assembly	13	0.0005	0.86
***Antigen presentation***			
GO:0019882∼antigen processing and presentation	13	0.0009	1.67
GO:0048002∼antigen processing & presentation of peptide antigen	10	0.0001	0.20
GO:0002474∼antigen processing & presentation via MHC I	7	0.0002	0.35
***Cell cycle***			
GO:0000278∼mitotic cell cycle	28	0.0011	2.00
GO:0022403∼cell cycle phase	36	0.0004	0.68
GO:0007049∼cell cycle	57	0.0004	0.72
GO:0022402∼cell cycle process	39	0.0015	2.67
***Hormone response***			
GO:0009725∼response to hormone stimulus	21	0.0009	1.50
GO:0009719∼response to endogenous stimulus	23	0.0006	1.11
GO:0043434∼response to peptide hormone stimulus	15	0.0008	1.33
***Synapse organization***			
GO:0050808∼synapse organization	10	0.0017	3.03
GO:0050803∼regulation of synapse structure and activity	6	0.0013	2.26
***Lipid metabolism***			
GO:0045834∼positive regulation of lipid metabolic process	7	0.0022	3.87

Enriched functional categories associated with aging. GO terms are ordered within subcategories by Fisher exact test p value (i.e., EASE score) with highest enriched term at the top of each list. False discovery rates (FDR) are expressed as percentage scores.

#### 3.3.2. Exercise-induced changes in hippocampal gene expression

Wheel running altered expression of 838 (526 upregulated and 312 downregulated; collapsed across age groups) genes (see Table S3). Functional analysis via DAVID and assignment to GO categories revealed that for both the adult and aged mice exercise influenced expression of genes that participate in chromatin remodeling, intracellular transport, growth, and protein autophosphorylation. A majority of the GO terms were related to chromatin remodeling. The top five enriched categories consisted of “cellular macromolecular complex assembly, chromatin assembly or disassembly, nucleosome assembly, chromatin organization, and chromatin assembly” (EASE score p<0.002 and FDR<5%; see Table 3).

**Table 3:** Exercise-induced changes.

GO Term	Gene count	P value	FDR
***Chromatin remodeling***			
GO:0034622∼cellular macromolecular complex assembly	22	0.0001	0.21
GO:0006333∼chromatin assembly or disassembly	14	0.0003	0.58
GO:0006334∼nucleosome assembly	11	0.0004	0.72
GO:0006325∼chromatin organization	27	0.0005	0.81
GO:0031497∼chromatin assembly	11	0.0005	0.92
GO:0065004∼protein-DNA complex assembly	11	0.0006	1.03
GO:0034728∼nucleosome organization	11	0.0006	1.03
GO:0034621∼cellular macromolecular complex subunit organization	22	0.0007	1.19
GO:0065003∼macromolecular complex assembly	27	0.0010	1.80
GO:0006323∼DNA packaging	12	0.0018	3.01
GO:0051276∼chromosome organization	30	0.0021	3.54
***Intracellular transport***			
GO:0046907∼intracellular transport	33	0.0006	1.12
***Regulation of growth***			
GO:0040008∼regulation of growth	22	0.0014	2.49
***Protein autophosphorylation***			
GO:0046777∼protein amino acid autophosphorylation	10	0.0014	2.39

Enriched functional categories associated with wheel running. GO terms are ordered within subcategories by p value (EASE score) with highest enriched term at the top of the list. FDRs are expressed as percentage scores.

#### 3.3.3. Opposing effects of age and exercise on hippocampal gene expression

**Figure 3:**
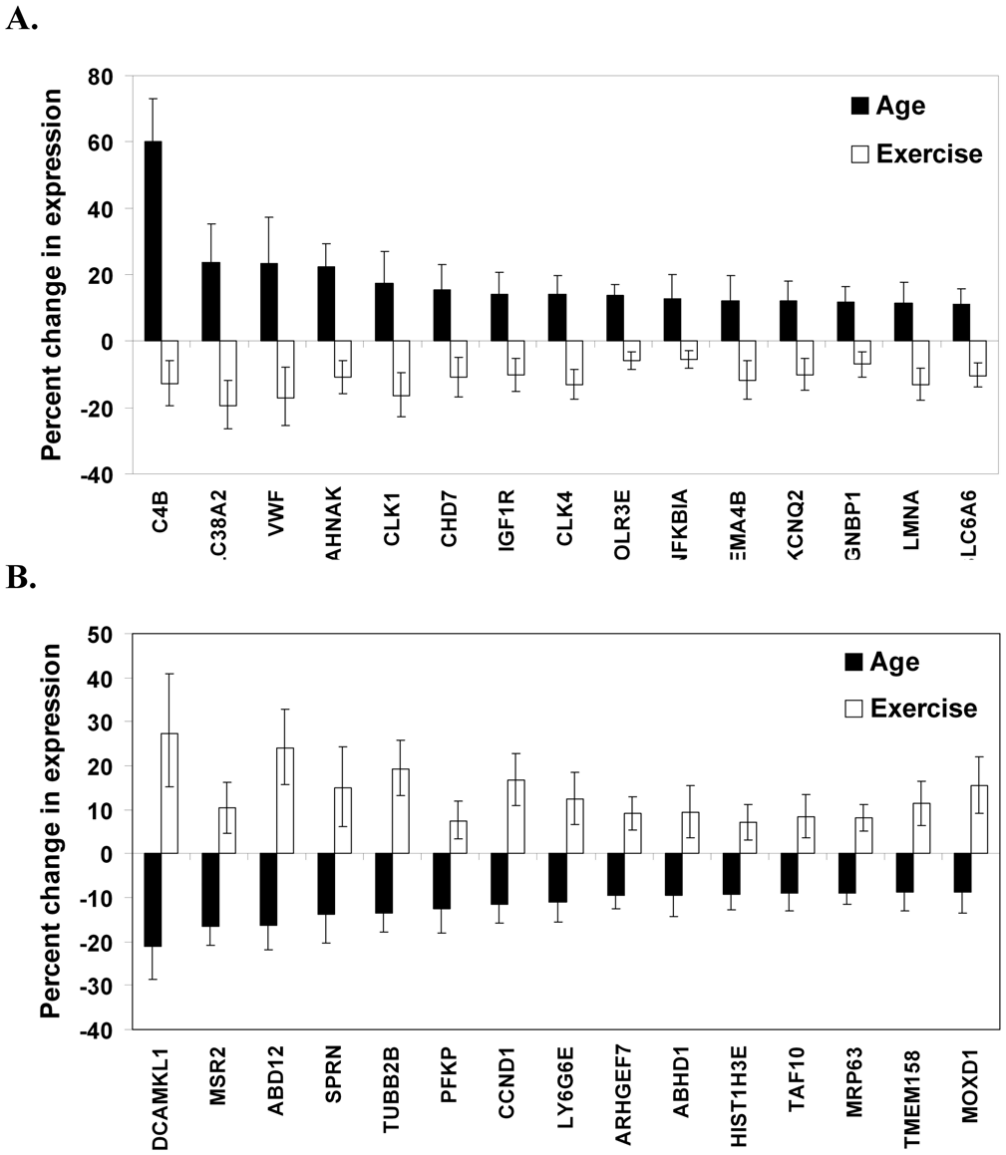
Differential regulation of gene expression by age and exercise. (A) Individual genes that showed the largest increase in expression in aged mice, and were downregulated by wheel running. (B) Genes that showed the largest decrease in expression in aged mice relative to adults, but were increased by exercise. Black bars represent percent change in gene expression in aged mice (collapsed across exercise condition) relative to adult mice (age comparison) ±95% confidence intervals. White bars represent percent change in gene expression in runners (collapsed across age groups) relative to sedentary mice (exercise comparison) ±95% confidence intervals. For gene description see Table 4.

Comparison of the genes altered by age or exercise revealed that 117 genes showed differential expression in response to aging and exercise (for complete list see Table S1). Analysis of the top 30 genes that showed the largest age-related increase or decrease in expression demonstrates that wheel running modulated expression of genes involved in a variety of physiological processes such as cell growth and migration, immune activity, chromatin organization, and mRNA translation. Aged mice showed a significant increase in expression of complement component 4B (C4B), solute carrier family 38 (SLC38A2), Von Willebrand factor (VWF), AHNAK nucleoprotein (AHNAK), CDC-like kinase 1 (CLK1), chromodomain helicase DNA binding protein 7 (CHD7), insulin-like growth factor receptor 1 (IGF1R), CDC-like kinase 4 (CLK4), polymerase III polypeptide (POLR3E), nuclear factor kappa polypeptide B-cell inhibitor (NFKBIA), sema domain immunoglobulin 4B (SEMA4B), potassium voltage-gated channel Q2 (KCNQ2), gametogenetin binding protein 1 (GGNBP1), lamin A (LMNA), and solute carrier family 6 (SLC6A6). Whereas wheel running significantly decreased expression of all of these genes (p<0.005; see Figure 3A and Table 4). Similarly, aged mice showed decreased expression of doublecortin-like kinase 1 (DCAMKL1), Fc receptor-like S (MSR2), abhydrolase domain containing 12 (ABD12), shadow of a prion protein (SPRN), tubulin beta 2 (TUBB2B), phosphofructokinase platelet (PFKP), cyclin D1 (CCND1), lymphocyte antigen complex 6 (LY6G6E), Rho guanine nucleotide exchange factor (ARHGEF7), abhydrolase domain containing 1 (ABHD1), histone cluster 2 H3e (HIST1H3E), TAF10 RNA polymerase II (TAF10), mitochondrial ribosomal protein 63 (MRP63), transmembrane protein 158 (TMEM158), and monooxygenase (MOXD1), however, wheel running increased expression of all of these genes (p<0.005; see Figure 3B and Table 4).

**Table 4:** Genes that showed differential expression from age and exercise.

Gene ID	Gene Name	% change Aged	% change Exercise
***Chromatin***			
HIST1H3E	histone cluster 2, H3e	−9.32	7.11
CHD7	chromodomain helicase DNA binding protein 7	15.20	−10.95
LMNA	lamin A	11.39	−13.06
***Cell growth/migration***			
DCAMKL1	doublecortin-like kinase 1	−21.17	27.34
TUBB2B	tubulin, beta 2a, pseudogene 2; tubulin, beta 2B	−13.43	19.24
CCND1	cyclin D1	−11.51	16.77
TMEM158	transmembrane protein 158	−8.86	11.38
SEMA4B	sema domain, immunoglobulin domain, transmembrane, 4B	12.07	−11.71
***Immune function***			
MSR2	Fc receptor-like S, scavenger receptor	−16.49	10.33
SPRN	shadow of prion protein	−13.90	14.95
LY6G6E	lymphocyte antigen 6 complex, locus G6E	−11.06	12.41
C4B	complement component 4B	59.96	−12.89
NFKBIA	nuclear factor kappa light polypeptide enhancer in B-cells inhibitor, α	12.81	−5.58
***Transcription/translation***			
TAF10	TAF10 RNA polymerase II, TATA box binding protein-associated factor	−9.01	8.41
CLK1	CDC-like kinase 1	17.40	−16.37
CLK4	CDC like kinase 4	13.92	−13.11
POLR3E	polymerase (RNA) III (DNA directed) polypeptide E	13.60	−5.87
***Blood glycoprotein***			
VWF	Von Willebrand factor homolog	23.38	−17.02
***Cell communication***			
KCNQ2	potassium voltage-gated channel, subfamily Q, member 2	11.96	−10.27
SLC6A6	solute carrier family 6 (neurotransmitter transporter, taurine)	11.10	−10.35
***Intracellular signaling***			
ARHGEF7	Rho guanine nucleotide exchange factor (GEF7)	−9.53	9.12
***Mitochondria***			
MRP63	mitochondrial ribosomal protein 63	−8.92	8.19
***Neuroblast differentiation***			
AHNAK	AHNAK nucleoprotein (desmoyokin)	22.40	−10.91
***Dopamine activity***			
MOXD1	monooxygenase, DBH-like 1	−8.69	15.50
***Endocannabinoid***			
ABD12	abhydrolase domain containing 12	−16.18	24.01
***Glutatmate activity***			
SLC38A2	solute carrier family 38, member 2	23.52	−19.50
***Gycolysis***			
PFKP	phosphofructokinase, platelet	−12.51	7.50
***IGF activity***			
IGF1R	insulin-like growth factor I receptor	13.93	−10.26
***Enzymatic function***			
ABHD1	abhydrolase domain containing 1	−9.50	9.43
***Spermatogenesis***			
GGNBP1	gametogenetin binding protein 1	11.64	−6.99

Functional categorization of the top 30 genes that showed differential expression in response to age and exercise. Positive values indicate the percent increase in expression and negative values indicate a decrease in expression.

### 3.4. qRT-PCR

Analysis revealed that BDNF, H2-D1, β2M, C4B, and GPX8 showed comparable expression when determined by RT-PCR or microarray, confirming results from the microarray experiment. Aged mice had significantly increased expression of β2M, H2-D1, and C4B compared to adult mice [main effects of Age (F(1,16) = 5.69;p<0.05) β2M, (F(1,16) = 16.63; p<0.001) H2-D1, and (F(1,14) = 121.69; p<0.001) C4B]. BDNF expression did not differ between adult and aged mice. Additionally, aged mice showed decreased expression of GPX8 (F(1,16) = 4.66; p<0.05) compared to adult mice. Wheel running was confirmed to significantly increase expression of BDNF (F(1,16) = 6.94;p<0.05) and decrease C4B expression (F(1,14) = 16.743.86;p<0.001) compared to sedentary mice.

## Discussion

The rate of brain aging appears to be dependent on lifestyle factors, as individuals that maintain an active healthy lifestyle show reduced risk for age-related neuropathologies [22]. The primary objective of this study was to identify genes and/or functional categories of genes that showed differential regulation in response to aging and exercise to provide insight into the anti-aging effects of exercise. Such knowledge may facilitate development of novel treatments to slow or prevent the effects of both normal and pathological aging.

We identified one-hundred and seventeen genes that showed differential regulation by age and exercise (see Table S1). Analysis of the top 30 genes revealed that several of them participate in cell growth and/or migration (see Figure 3). Aging is well known to disrupt cellular division. For instance, the rate of neurogenesis is drastically reduced with aging [2], [5], [11]. In agreement, we found that aged mice showed reduced expression of several genes involved in cellular mitosis, such as cyclin D1 (CCND1), cell division cycle associated 2 (CDC2), cell division cycle associated 8 (CDC8), leishmanolysin-like (LMLN), and baculoviral IAP repeat-containing 5 (BIRC5) (see Table S2). These data in combination with prior work that show reduced hippocampal neurogenesis in aged animals [2], [5], [11] indicate that aging is associated with reduced cellular proliferation.

Engaging in aerobic exercise is known to enhance hippocampal neurogenesis in both young and aged animals [2], [17], [18]. Our data provide potential transcription changes that may contribute to the exercise-induced increase in neurogenesis. A portion of the age-related changes in genes related to cell grow showed enhanced expression in response to exercise. For example, aged mice showed reduced expression of doublecortin-like kinase-1 (DCAMKL1), cyclin D1 (CCND1), and tublin β2 (TUBB2B) compared to adult mice, whereas exercise was found to upregulate these genes in both adult and aged mice. DCAMKL1 participates in several cellular processes such as neurogenesis, neural migration, and retrograde transport [40]. TUBB2B is a microtubule element expressed mainly in post-mitotic neurons and CCND1 participates in cellular proliferation by facilitating progression through the cell-cycle [41]. Collectively, these findings suggest that exercise can restore the proliferative capacity of the hippocampus and highlight potential transcription alterations that may underlie this effect.

By far the largest category of genes modified by age was chromatin remodeling. Maintaining the structure of chromatin is crucial for normal transcription. Our data show an age-related decline in the expression of genes for histone proteins, the core components of nucleosomes, and increased expression of genes for ATP-dependent chromatin remodelers chromatin helicase DNA-binding protein 7 (CHD7) and SWI/SNF related, matrix associated, actin dependent regulator of chromatin (SMARCD2) [42], [43]. Prior work suggests that the age-related reduction in histone proteins may loosen the chromatin structure increasing the likelihood of aberrant transcription and access to agents that can damage DNA [44]. The observed increase in CHD7 expression in combination with the reduction in histone expression in the aged may indicate increased transcription. In accordance we observed increased expression of over 500 genes in the aged compared to the adult mice. There is evidence in yeast cells that restoring histone levels increases lifespan, indicating that aspects of aging can be improved by normalizing chromatin structure [45].

In addition to transcriptional changes, abnormal mRNA translation may increase with age. In support, we observed that aged mice showed increased expression of CDC-like kinase-1 (CLK-1) and CDC-like kinase-4 (CLK-4) that appear to regulate mRNA splicing through altering the activity of splicesomes [46]. Improper RNA splicing can result in abnormal translation of RNA and is associated with many age-related diseases including macular degeneration and Alzheimer’s disease [47]. Additionally, aged mice showed reduced expression of TAF10 RNA polymerase (TAF10) that plays a role initiating transcription [48] and decreased expression of RNA polymerase III (POLR3E) that participates in pre-mRNA splicing and transcription [49]. While we cannot confirm from the present data whether aging increases the frequency of improper RNA slicing, the results indicate that pre-mRNA splicing may be vulnerable to age-related alterations.

Exercise was found to differentially influence expression of many of the genes involved in chromatin remodeling and transcription that showed an age-related change in expression. For example, aged mice showed reduced expression of the histone protein H3E, but wheel running was found to increase H3E expression. Additionally, we found that exercise reduced the expression of CHD7, which showed increased expression in hippocampal samples from the aged animals. Exercise reduced expression of the genes involved in pre-mRNA splicing CLK-1, CLK-4, and POLR3E and increased expression of TAF10 that participates in basal transcription. These data indicate that exercise may restore age-related changes in chromatin modification and possibly prevent these changes in adults both of which would better protect DNA and regulate transcription.

Aging is associated with the development of low-grade neuroinflammation that may result from increased activation of the brain’s resident immune cells, microglia [14], [50]. In agreement, our findings indicate an age-related increase in immune activity within the brain [14], [51], [52]. For example, a major group of genes upregulated in the aged mice were related to the major histocompatibility complex (MHC) class I receptor (e.g., β2-microglubin and H2-D1). In addition, aged mice showed increased expression of several elements of the complement system including complement component 4A (C4A), C4B, C3, and C1q, which facilitate phagocytosis of cells or bacteria through opsonization. C1q binds to neurons as they have low expression of complement resistant molecules such as DAF and CD59 [53]. This raises the possibility that neurons in the aged brain may be at greater risk of destruction through C1q labeling. Additionally, increased expression of C1, C3 and C4 mRNA has been found in the brains of Alzheimer patients, and is thought to contribute to the progression of Alzheimer’s disease by inducing microglia activation and proinflammatory cytokine release [54]. Collectively, these age-related changes in neuroinflammation may contribute to the increased vulnerability to age-related cognitive decline and the progression of neurodegenerative diseases.

Exercise may offer neuroprotection through regulating aspects of immune activity. Prior studies have found that exercise in adult subjects increases expression of anti-inflammatory molecules while reducing inflammatory mediators [14], [29], [52], [55]. Our data show that wheel running reduced expression of C4B which is released from microglia following an immune stimulus [56]. Additionally, wheel running enhanced expression of shadow of a prion protein (SPRN), a gene that encodes for the protein Sho that has neuroprotective-like effects against infection with a prion [57]. SPRN expression was reduced in the aged mice, but was elevated in response to exercise. Though more work is needed to fully elucidate the ability of exercise to attenuate neuroinflammation, particularly in aged subjects, these data provide evidence that exercise may afford some protection by modulating immune activity within the brain.

The trophic factor, insulin-like growth factor (IGF), plays a complex role in the aging process. Research on age-related alterations in IGF levels in the brain has show inconsistent results, as some report a decrease whereas others fail to detect a difference [58], [59]. However, there is evidence to suggest that expression of the IGF type I receptor (IGFR1) increases with age [58]. Our findings confirm this result, as aged mice showed increased expression of IGFR1 in the hippocampus, and additionally show that wheel running reduced IGFR1 expression. The age-related increase in IGFR1 may occur in response to low IGF levels or may be a compensatory mechanism to overcome resistance or functional deficits in the receptor signaling cascade [60], [61]. Given that exercise increases expression of IGF [62], the exercise-induced reduction in IGFR1 may result from stabilizing trophic support in the aged brain.

Alterations in mitochondria function are a central theory of aging. Mitochondria are a primary energy source throughout the body and therefore a decline in their function disrupts normal cellular activity and could ultimately lead to cell death [63]. In support, we observed that aged mice showed reduced expression of mitochondrial ribosomal protein 63 (MPR63), which is known to aid in protein synthesis within mitochondria [64], [65]. Further, we observed that exercise increased expression of MRP63. This finding is in agreement with prior work that indicates some of the beneficial effects of exercise are mediated through its influence on mitochondria [63].

In agreement with prior reports, wheel running significantly increased expression of brain derived neurotrophic factor (BDNF) in both adult and aged mice [29]. BDNF is believed to mediate the beneficial effects of exercise on cell growth, proliferation, and possibly cognitive enhancements [66], [67]. In addition to BDNF, we observed that a GO term related to growth was significantly enriched from exercise. For example, wheel running was found to increase expression of poly (ADP-ribose) polymerase 1 (PARP1) and RuvB-like protein 1 (RUVBL1), genes involved in repairing damage to DNA and maintaining genomic stability [68], [69]. Exercise was found to suppress expression of BCL2 binding component 3 (BBC3), a gene that can initiate apoptosis [70]. These findings confirm that exercise independent of age supports brain health by initiating growth and protection against destructive events.

Collectively, these data highlight transcriptional changes that may mediate the anti-aging effects of exercise. Our findings confirm prior microarray experiments that assessed gene transcription changes in response to exercise [29], aging [13], [14], [52], [71], or exercise only in aged mice [28]. Ultimately, our findings indicate that the beneficial effects of exercise likely result from changes in multiple pathways that may be restorative in aged subjects, but also act as a preventive measure in younger subjects. The data emphasize that effective anti-aging treatments need to combat a complex array of changes. Wheel running was found to regulate chromatin structure, cell growth, immune activity, and trophic factors opposing many of the age-related changes in these categories. Findings argue that the therapeutic effects of exercise likely results from its ability to modulate a broad range of processes that are altered by normal aging.

## Supporting Information

Table S1 **List the individual genes that showed differential regulation in response to aging and wheel running.** The percent change in aged column shows the percent change in gene expression in aged mice (collapsed across exercise condition) relative to adult mice (age comparison) ±95% confidence intervals. The percent change in exercise column list the percent change in gene expression in runners (collapsed across age groups) relative to sedentary mice (exercise comparison) ±95% confidence intervals.
(DOC)

Table S2 **Age-induced changes in gene expression in the hippocampus.** Columns list the percent change in gene expression in aged mice (collapsed across exercise condition) relative to adult mice ±95% confidence intervals for an individual gene. Positive values indicate the percent increase in expression and negative values indicate a decrease in expression. FDRs are expressed as percentage scores.
(DOC)

Table S3 **Exercise-induced changes in gene expression in the hippocampus.** Columns list the percent change in gene expression in runners (collapsed across age) compared to sedentary mice ±95% confidence intervals for an individual gene. Positive values indicate the percent increase in expression and negative values indicate a decrease in expression. FDRs are expressed as percentage scores.
(DOC)
