# Supplementary material for: Voluntary Wheel Running Reverses Age-Induced Changes in Hippocampal Gene Expression
Source: PLoS One. 2011 Aug 8;6(8):e22654. doi: 10.1371/journal.pone.0022654 (PMC3152565; doi:10.1371/journal.pone.0022654)
Supplement: Table S1 — List the individual genes that showed differential regulation in response to aging and wheel running. The percent change in aged column shows the percent change in gene expression in aged mice (collapsed across exercise condition) relative to adult mice (age comparison) ±95% confidence intervals. The percent change in exercise column list the percent change in gene expression in runners (collapsed across age groups) relative to sedentary mice (exercise comparison) ±95% confidence intervals. (DOC) [file pone.0022654.s001.doc]

| **Supplementary Table S1.** | |  |  | | | | | | |  |  |  |  |  |  |
| --- | --- | --- | --- | --- | --- | --- | --- | --- | --- | --- | --- | --- | --- | --- | --- |
|  | |  |  | | | | | | |  | | | | | |
| **Downregulated in aged and upregulated from exercise** | | ***95% confidence intervals*** | | |  | | ***95% confidence intervals*** | |  |  | | | | | |
| ***Gene name*** | ***Percent change in Aged*** | ***Lower Age*** | | ***Upper Age*** | ***FDR Age*** | ***Percent change in Exercised*** | ***Lower Exercise*** | ***Upper Exercise*** | ***FDR Exercise*** |  | | | | | |
| doublecortin-like kinase 1 | -21.17 | -12.81 | | -28.74 | 0.03 | 27.34 | 15.12 | 40.85 | 0.08 |  | | | | | |
| Fc receptor-like S, scavenger receptor | -16.49 | -11.96 | | -20.80 | 0.00 | 10.33 | 4.65 | 16.32 | 0.17 |  | | | | | |
| abhydrolase domain containing 12 | -16.18 | -10.19 | | -21.78 | 0.02 | 24.01 | 15.70 | 32.91 | 0.03 |  | | | | | |
| shadow of prion protein | -13.90 | -6.82 | | -20.45 | 0.09 | 14.95 | 6.21 | 24.41 | 0.19 |  | | | | | |
| tubulin, beta 2a, pseudogene 2; tubulin, beta 2B | -13.43 | -8.77 | | -17.86 | 0.02 | 19.24 | 13.09 | 25.72 | 0.03 |  | | | | | |
| phosphofructokinase, platelet | -12.51 | -6.58 | | -18.07 | 0.07 | 7.50 | 3.27 | 11.91 | 0.18 |  | | | | | |
| cyclin D1 | -11.51 | -6.96 | | -15.84 | 0.03 | 16.77 | 11.02 | 22.83 | 0.03 |  | | | | | |
| lymphocyte antigen 6 complex, locus G6E | -11.06 | -6.37 | | -15.52 | 0.04 | 12.41 | 6.75 | 18.37 | 0.10 |  | | | | | |
| Rho guanine nucleotide exchange factor (GEF7) | -9.53 | -6.43 | | -12.54 | 0.01 | 9.12 | 5.47 | 12.90 | 0.06 |  | | | | | |
| abhydrolase domain containing 1 | -9.50 | -4.50 | | -14.23 | 0.10 | 9.43 | 3.68 | 15.49 | 0.22 |  | | | | | |
| histone cluster 2, H3e | -9.32 | -5.80 | | -12.70 | 0.02 | 7.11 | 3.09 | 11.29 | 0.18 |  | | | | | |
| TAF10 RNA polymerase II, TATA box binding protein (TBP)-associated factor | -9.01 | -4.82 | | -13.01 | 0.06 | 8.41 | 3.61 | 13.43 | 0.18 |  | | | | | |
| mitochondrial ribosomal protein 63 | -8.92 | -6.34 | | -11.44 | 0.01 | 8.19 | 5.19 | 11.27 | 0.05 |  | | | | | |
| transmembrane protein 158 | -8.86 | -4.63 | | -12.91 | 0.07 | 11.38 | 6.43 | 16.55 | 0.08 |  | | | | | |
| monooxygenase, DBH-like 1 | -8.69 | -3.54 | | -13.56 | 0.15 | 15.50 | 9.29 | 22.06 | 0.06 |  | | | | | |
| dynactin 5 | -8.51 | -5.58 | | -11.34 | 0.01 | 7.80 | 4.43 | 11.28 | 0.08 |  | | | | | |
| similar to Protein disulfide isomerase associated 6 | -8.38 | -4.74 | | -11.88 | 0.04 | 11.32 | 7.07 | 15.75 | 0.05 |  | | | | | |
| mitochondrial ribosomal protein S16; predicted gene 9173 | -8.25 | -5.37 | | -11.05 | 0.02 | 6.17 | 2.93 | 9.52 | 0.16 |  | | | | | |
| small nuclear ribonucleoprotein polypeptide A | -8.22 | -3.83 | | -12.42 | 0.11 | 5.94 | 2.43 | 9.57 | 0.20 |  | | | | | |
| syntaxin 12 | -8.17 | -3.51 | | -12.60 | 0.13 | 8.94 | 3.68 | 14.46 | 0.20 |  | | | | | |
| RIKEN cDNA 2210008F06 gene | -7.80 | -3.93 | | -11.50 | 0.08 | 8.09 | 3.74 | 12.62 | 0.16 |  | | | | | |
| translocase of outer mitochondrial membrane 40 homolog-like (yeast) | -7.74 | -4.90 | | -10.50 | 0.02 | 5.72 | 2.54 | 9.01 | 0.18 |  | | | | | |
| leucine rich repeat containing 7 | -7.55 | -4.29 | | -10.70 | 0.04 | 8.69 | 4.95 | 12.56 | 0.08 |  | | | | | |
| transmembrane protein 183A | -7.13 | -3.60 | | -10.54 | 0.08 | 9.88 | 6.65 | 13.20 | 0.03 |  | | | | | |
| insulin-like growth factor binding protein-like 1 | -7.06 | -5.68 | | -8.42 | 0.00 | 5.67 | 4.12 | 7.24 | 0.02 |  | | | | | |
| RIKEN cDNA B230311B06 gene | -6.93 | -3.76 | | -10.00 | 0.05 | 5.73 | 2.21 | 9.36 | 0.23 |  | | | | | |
| inositol polyphosphate-4-phosphatase, type I | -6.89 | -4.18 | | -9.53 | 0.03 | 7.60 | 4.53 | 10.77 | 0.07 |  | | | | | |
| ras homolog gene family, member T1 | -6.80 | -2.85 | | -10.59 | 0.14 | 8.37 | 3.93 | 12.99 | 0.16 |  | | | | | |
| vesicle transport through interaction with t-SNAREs homolog 1A (yeast) | -6.78 | -2.73 | | -10.66 | 0.15 | 3.30 | 1.31 | 5.33 | 0.22 |  | | | | | |
| RIKEN cDNA 4933403G14 gene | -6.73 | -2.70 | | -10.59 | 0.15 | 10.14 | 5.58 | 14.89 | 0.10 |  | | | | | |
| expressed sequence AU022252 | -6.56 | -3.57 | | -9.47 | 0.05 | 6.86 | 2.81 | 11.07 | 0.20 |  | | | | | |
| dehydrogenase/reductase (SDR family) member 7B | -6.54 | -3.57 | | -9.41 | 0.05 | 8.05 | 4.72 | 11.48 | 0.07 |  | | | | | |
| predicted gene 715 | -6.43 | -4.24 | | -8.56 | 0.01 | 4.74 | 2.35 | 7.18 | 0.14 |  | | | | | |
| peptidylprolyl isomerase (cyclophilin)-like 3 | -6.34 | -2.75 | | -9.79 | 0.13 | 2.41 | 1.08 | 3.76 | 0.18 |  | | | | | |
| transforming growth factor beta regulated gene 1 | -6.31 | -3.21 | | -9.32 | 0.07 | 6.02 | 3.49 | 8.61 | 0.07 |  | | | | | |
| testis specific gene A14 | -6.27 | -3.64 | | -8.84 | 0.04 | 7.60 | 4.63 | 10.66 | 0.06 |  | | | | | |
| transmembrane protein 70 | -6.22 | -2.67 | | -9.64 | 0.13 | 9.86 | 5.85 | 14.03 | 0.07 |  | | | | | |
| coiled-coil-helix-coiled-coil-helix domain containing 8 | -6.10 | -3.76 | | -8.37 | 0.02 | 4.77 | 2.21 | 7.38 | 0.16 |  | | | | | |
| CLPTM1-like | -6.10 | -3.14 | | -8.97 | 0.07 | 6.27 | 3.00 | 9.66 | 0.15 |  | | | | | |
| solute carrier family 25 (mitochondrial carrier, brain), member 14 | -6.07 | -2.52 | | -9.50 | 0.14 | 7.28 | 3.34 | 11.37 | 0.17 |  | | | | | |
| histone cluster 2, H2ab | -5.97 | -3.76 | | -8.13 | 0.02 | 4.57 | 3.34 | 11.37 | 0.16 |  | | | | | |
| metallo-beta-lactamase domain containing 1 | -5.77 | -2.72 | | -8.72 | 0.10 | 6.31 | 2.98 | 9.75 | 0.16 |  | | | | | |
| phosphatidylserine synthase 2 | -5.62 | -2.94 | | -8.22 | 0.06 | 3.07 | 1.28 | 4.90 | 0.20 |  | | | | | |
| nudix (nucleoside diphosphate linked moiety X)-type motif 5 | -5.54 | -2.20 | | -8.76 | 0.15 | 11.04 | 5.02 | 17.41 | 0.17 |  | | | | | |
| histone cluster 2, H2be | -5.51 | -2.43 | | -8.48 | 0.12 | 5.52 | 2.18 | 8.97 | 0.22 |  | | | | | |
| transmembrane protein 107 | -5.50 | -2.65 | | -8.27 | 0.09 | 5.10 | 2.17 | 8.12 | 0.19 |  | | | | | |
| RIKEN cDNA 2310033P09 gene | -5.31 | -2.19 | | -8.33 | 0.14 | 7.67 | 3.90 | 11.58 | 0.13 |  | | | | | |
| REX2, RNA exonuclease 2 homolog (S. cerevisiae) | -5.29 | -2.44 | | -8.06 | 0.11 | 7.32 | 4.18 | 10.55 | 0.08 |  | | | | | |
| transmembrane protein 63c | -5.26 | -3.26 | | -7.22 | 0.02 | 5.46 | 3.26 | 7.71 | 0.07 |  | | | | | |
| mitochondrial ribosomal protein L15 | -5.25 | -2.74 | | -7.71 | 0.07 | 5.03 | 2.50 | 7.61 | 0.13 |  | | | | | |
| similar to Manbal protein; mannosidase, beta A, lysosomal-like | -5.25 | -2.78 | | -7.66 | 0.06 | 6.50 | 3.78 | 9.29 | 0.07 |  | | | | | |
| KN motif and ankyrin repeat domains 3 | -5.11 | -2.12 | | -8.00 | 0.14 | 5.68 | 2.44 | 9.02 | 0.19 |  | | | | | |
| neighbor of Brca1 gene 1 | -5.11 | -2.64 | | -7.50 | 0.07 | 5.67 | 3.00 | 8.41 | 0.11 |  | | | | | |
| predicted gene 5626; stomatin (Epb7.2)-like 2 | -4.79 | -2.46 | | -7.06 | 0.07 | 4.77 | 2.25 | 7.35 | 0.16 |  | | | | | |
| oligodendrocyte myelin glycoprotein | -4.36 | -2.34 | | -6.34 | 0.06 | 7.71 | 5.46 | 10.02 | 0.02 |  | | | | | |
| cyclin D binding myb-like transcription factor 1 | -4.35 | -1.81 | | -6.83 | 0.14 | 5.12 | 2.78 | 7.51 | 0.11 |  | | | | | |
| RIKEN cDNA 2310075K07 gene | -4.04 | -2.11 | | -5.94 | 0.06 | 3.41 | 1.35 | 5.52 | 0.22 |  | | | | | |
| WAP four-disulfide core domain 3 | -3.98 | -1.85 | | -6.06 | 0.10 | 4.53 | 2.24 | 6.86 | 0.14 |  | | | | | |
| FXYD domain-containing ion transport regulator 2 | -3.83 | -1.56 | | -6.05 | 0.14 | 5.90 | 3.44 | 8.42 | 0.07 |  | | | | | |
| cell division cycle associated 8 | -3.67 | -1.46 | | -5.83 | 0.15 | 3.84 | 1.49 | 6.24 | 0.23 |  | | | | | |
| HIG1 domain family, member 1B | -3.48 | -1.66 | | -5.26 | 0.09 | 3.20 | 1.28 | 5.16 | 0.22 |  | | | | | |
| RIKEN cDNA 2610017I09 gene | -16.63 | -7.73 | | -24.67 | 0.11 | 24.43 | 12.34 | 37.82 | 0.12 |  | | | | | |
| predicted gene 5617 | -9.74 | -6.05 | | -13.29 | 0.02 | 8.64 | 4.34 | 13.12 | 0.13 |  | | | | | |
| predicted gene 9731; RIKEN cDNA 1810009O10 gene | -8.77 | -4.81 | | -12.58 | 0.05 | 8.01 | 3.48 | 12.73 | 0.18 |  | | | | | |
| predicted gene 5064; cold shock domain containing E1, RNA binding | -9.48 | -4.76 | | -13.96 | 0.08 | 11.00 | 5.51 | 16.78 | 0.13 |  | | | | | |
| YdjC homolog (bacterial) | -9.09 | -4.97 | | -13.02 | 0.05 | 9.42 | 4.66 | 14.39 | 0.13 |  | | | | | |

| **Upregulated in aged and downregulated from exercise** |  | ***95% confidence intervals*** | |  |  | ***95% confidence intervals*** | |  |
| --- | --- | --- | --- | --- | --- | --- | --- | --- |
| ***Gene name*** | ***Percent change in Aged*** | ***Lower Age*** | ***Upper Age*** | ***FDR Age*** | ***Percent change Exercise*** | ***Lower Exercise*** | ***Upper Exercise*** | ***FDR Exercise*** |
| complement component 4B | 59.96 | 47.89 | 73.01 | 0.00 | -12.89 | -5.77 | -19.48 | 0.19 |
| solute carrier family 38, member 2 | 23.52 | 12.77 | 35.29 | 0.04 | -19.50 | -11.83 | -26.50 | 0.08 |
| Von Willebrand factor homolog | 23.38 | 11.00 | 37.15 | 0.07 | -17.02 | -7.76 | -25.35 | 0.19 |
| AHNAK nucleoprotein (desmoyokin) | 22.40 | 15.75 | 29.44 | 0.00 | -10.91 | -5.75 | -15.78 | 0.13 |
| CDC-like kinase 1 | 17.40 | 8.48 | 27.07 | 0.07 | -16.37 | -9.49 | -22.73 | 0.10 |
| chromodomain helicase DNA binding protein 7 | 15.20 | 7.87 | 23.04 | 0.05 | -10.95 | -4.84 | -16.67 | 0.19 |
| insulin-like growth factor I receptor | 13.93 | 7.68 | 20.55 | 0.04 | -10.26 | -5.04 | -15.19 | 0.16 |
| CDC like kinase 4 | 13.92 | 8.35 | 19.79 | 0.02 | -13.11 | -8.63 | -17.36 | 0.05 |
| polymerase (RNA) III (DNA directed) polypeptide E | 13.60 | 10.33 | 16.97 | 0.00 | -5.87 | -3.06 | -8.59 | 0.13 |
| nuclear factor of kappa light polypeptide gene enhancer in B-cells inhibitor, alpha | 12.81 | 6.00 | 20.05 | 0.08 | -5.58 | -2.90 | -8.20 | 0.13 |
| sema domain, immunoglobulin domain, transmembrane and short cytoplasmic , 4B | 12.07 | 4.98 | 19.64 | 0.13 | -11.71 | -5.75 | -17.30 | 0.16 |
| potassium voltage-gated channel, subfamily Q, member 2 | 11.96 | 6.11 | 18.14 | 0.06 | -10.27 | -5.32 | -14.96 | 0.13 |
| gametogenetin binding protein 1; RIKEN cDNA 0610031G08 gene | 11.64 | 7.12 | 16.34 | 0.02 | -6.99 | -3.07 | -10.75 | 0.19 |
| lamin A | 11.39 | 5.43 | 17.68 | 0.08 | -13.06 | -8.13 | -17.73 | 0.06 |
| solute carrier family 6 (neurotransmitter transporter, taurine), member 6 | 11.10 | 6.76 | 15.61 | 0.02 | -10.35 | -6.68 | -13.87 | 0.05 |
| filamin, beta | 10.55 | 4.23 | 17.25 | 0.13 | -10.52 | -5.05 | -15.68 | 0.17 |
| kelch-like 21 (Drosophila) | 10.55 | 5.12 | 16.25 | 0.07 | -8.60 | -3.85 | -13.12 | 0.19 |
| nanos homolog 2 (Drosophila) | 10.42 | 7.23 | 13.71 | 0.00 | -7.24 | -4.47 | -9.92 | 0.06 |
| SAPS domain family, member 3 | 10.29 | 6.15 | 14.59 | 0.02 | -6.17 | -4.47 | -9.92 | 0.22 |
| period homolog 3 (Drosophila) | 10.17 | 5.61 | 14.92 | 0.04 | -8.31 | -4.33 | -12.11 | 0.13 |
| glypican 5 | 9.55 | 4.82 | 14.49 | 0.06 | -6.17 | -4.12 | -8.17 | 0.04 |
| pyruvate dehydrogenase kinase, isoenzyme 4 | 9.40 | 4.44 | 14.61 | 0.08 | -11.89 | -7.69 | -15.90 | 0.05 |
| ATPase, class V, type 10A | 9.26 | 6.24 | 12.37 | 0.01 | -5.30 | -2.61 | -7.93 | 0.15 |
| zinc finger, FYVE domain containing 21 | 8.91 | 4.34 | 13.68 | 0.07 | -12.72 | -8.87 | -16.41 | 0.03 |
| peptidyl arginine deiminase, type II | 8.67 | 4.03 | 13.51 | 0.09 | -5.39 | -2.30 | -8.38 | 0.20 |
| CDC14 cell division cycle 14 homolog B (S. cerevisiae) | 8.50 | 4.52 | 12.63 | 0.05 | -7.23 | -3.70 | -10.63 | 0.14 |
| elongation factor RNA polymerase II | 8.48 | 5.35 | 11.71 | 0.02 | -5.82 | -3.00 | -8.57 | 0.13 |
| oviductal glycoprotein 1 | 8.14 | 4.14 | 12.30 | 0.06 | -7.90 | -4.36 | -11.31 | 0.11 |
| matrix-remodelling associated 7 | 8.10 | 3.23 | 13.20 | 0.14 | -7.83 | -3.47 | -12.01 | 0.19 |
| Rap guanine nucleotide exchange factor (GEF) 3 | 8.09 | 4.52 | 11.79 | 0.04 | -7.91 | -4.74 | -10.98 | 0.07 |
| UDP-GalNAc:betaGlcNAc beta 1,3-galactosaminyltransferase, polypeptide 2 | 8.03 | 3.39 | 12.87 | 0.12 | -3.98 | -2.17 | -5.76 | 0.11 |
| Sec24 related gene family, member B (S. cerevisiae) | 7.96 | 4.80 | 11.21 | 0.02 | -4.94 | -2.06 | -7.73 | 0.21 |
| RAB5B, member RAS oncogene family | 7.66 | 3.51 | 11.98 | 0.10 | -7.17 | -3.42 | -10.78 | 0.17 |
| dynamin 1 | 7.61 | 4.14 | 11.21 | 0.04 | -8.12 | -5.03 | -11.12 | 0.06 |
| choline kinase beta | 7.49 | 4.36 | 10.72 | 0.03 | -4.85 | -1.96 | -7.64 | 0.22 |
| peroxisomal delta3, delta2-enoyl-Coenzyme A isomerase | 7.07 | 3.82 | 10.42 | 0.05 | -5.44 | -2.45 | -8.34 | 0.18 |
| ribosomal protein L23 | 6.92 | 3.11 | 10.88 | 0.10 | -5.91 | -2.39 | -9.30 | 0.22 |
| regulator of G-protein signalling 9 binding protein | 6.85 | 2.94 | 10.92 | 0.12 | -6.56 | -3.01 | -9.99 | 0.18 |
| neuralized homolog 1A (Drosophila); similar to neuralized 1 | 6.07 | 3.15 | 9.08 | 0.06 | -6.90 | -4.26 | -9.46 | 0.06 |
| predicted gene 5747; RIKEN cDNA 1810026J23 gene | 5.91 | 2.91 | 8.99 | 0.07 | -5.28 | -2.49 | -7.98 | 0.17 |
| amiloride-sensitive cation channel 1, neuronal (degenerin) | 5.88 | 2.37 | 9.52 | 0.14 | -5.72 | -2.49 | -8.85 | 0.19 |
| methyl CpG binding protein 2 | 5.85 | 2.49 | 9.32 | 0.12 | -5.58 | -2.47 | -8.60 | 0.19 |
| lipin 1 | 5.64 | 2.39 | 8.98 | 0.12 | -6.51 | -3.01 | -9.88 | 0.18 |
| family with sequence similarity 82, member B | 5.27 | 2.84 | 7.75 | 0.05 | -3.96 | -1.68 | -6.19 | 0.20 |
| fat mass and obesity associated | 5.18 | 2.09 | 8.36 | 0.14 | -7.24 | -2.91 | -11.38 | 0.23 |
| MAP/microtubule affinity-regulating kinase 3 | 4.81 | 2.13 | 7.56 | 0.11 | -4.45 | -1.92 | -6.91 | 0.20 |
| molybdenum cofactor synthesis 1 | 4.57 | 2.05 | 7.16 | 0.11 | -6.78 | -4.48 | -9.03 | 0.04 |
| tumor necrosis factor, alpha-induced protein 3 | 3.34 | 1.32 | 5.41 | 0.15 | -3.74 | -1.81 | -5.64 | 0.16 |
| mucosa associated lymphoid tissue lymphoma translocation gene 1 | 3.02 | 1.44 | 4.64 | 0.09 | -3.08 | -1.55 | -4.59 | 0.14 |
| similar to mKIAA1021 protein | 12.30 | 5.70 | 19.32 | 0.09 | -10.82 | -5.20 | -16.11 | 0.17 |
| RIKEN cDNA 1500012F01 gene | 11.25 | 5.60 | 17.20 | 0.07 | -12.72 | -6.07 | -18.90 | 0.17 |
