# Supplementary material for: Voluntary Wheel Running Reverses Age-Induced Changes in Hippocampal Gene Expression
Source: PLoS One. 2011 Aug 8;6(8):e22654. doi: 10.1371/journal.pone.0022654 (PMC3152565; doi:10.1371/journal.pone.0022654)
Supplement: Table S2 — Age-induced changes in gene expression in the hippocampus. Columns list the percent change in gene expression in aged mice (collapsed across exercise condition) relative to adult mice ±95% confidence intervals for an individual gene. Positive values indicate the percent increase in expression and negative values indicate a decrease in expression. FDRs are expressed as percentage scores. (DOC) [file pone.0022654.s002.doc]

| **Supplementary Table S2.** | | | | | |
| --- | --- | --- | --- | --- | --- |
| Age-induced changes in gene expression in the hippocampus  ***Downregulated*** | | **95% confidence intervals** | |  |  |
| **Percent change** | **Gene name** | **Lower** | **Upper** | **FDR** | **P value** |
| -31.18 | glutathione peroxidase 8 (putative) | -21.75 | -39.47 | 0.01 | 0.000026 |
| -30.47 | G protein-coupled receptor 17 | -24.66 | -35.84 | 0.00 | 0.000000 |
| -24.12 | DNA segment, human D4S114 | -12.56 | -34.15 | 0.08 | 0.001382 |
| -23.87 | glycine receptor, alpha 2 subunit | -12.76 | -33.56 | 0.07 | 0.001091 |
| -22.51 | carbonic anyhydrase 12 | -9.96 | -33.32 | 0.14 | 0.003977 |
| -22.35 | histone cluster 1, H1c | -14.72 | -29.30 | 0.02 | 0.000060 |
| -22.17 | Fc receptor-like S, scavenger receptor | -16.81 | -27.18 | 0.00 | 0.000001 |
| -22.10 | thymosin, beta 10 | -10.09 | -32.51 | 0.13 | 0.003316 |
| -21.17 | doublecortin-like kinase 1 | -12.81 | -28.74 | 0.03 | 0.000242 |
| -19.55 | claudin 5 | -12.88 | -25.70 | 0.02 | 0.000053 |
| -19.23 | transmembrane protein 114 | -9.21 | -28.14 | 0.11 | 0.002312 |
| -18.85 | transferrin receptor | -13.99 | -23.44 | 0.00 | 0.000002 |
| -18.77 | actin, alpha 2, smooth muscle, aorta | -12.12 | -24.92 | 0.02 | 0.000076 |
| -18.29 | Ly6/Plaur domain containing 1 | -7.59 | -27.74 | 0.15 | 0.005044 |
| -18.02 | histone cluster 1, H2ad | -12.55 | -23.14 | 0.01 | 0.000013 |
| -17.85 | myocilin | -10.71 | -24.43 | 0.04 | 0.000244 |
| -17.66 | protein tyrosine phosphatase, non-receptor type 15; receptor type, O | -11.70 | -23.22 | 0.02 | 0.000043 |
| -16.90 | Rho family GTPase 2; predicted gene 4768 | -9.59 | -23.62 | 0.05 | 0.000482 |
| -16.65 | myristoylated alanine rich protein kinase C substrate | -10.01 | -22.81 | 0.03 | 0.000228 |
| -16.63 | RIKEN cDNA 2610017I09 gene | -7.73 | -24.67 | 0.11 | 0.002665 |
| -16.49 | Fc receptor-like S, scavenger receptor | -11.96 | -20.80 | 0.00 | 0.000004 |
| -16.18 | abhydrolase domain containing 12 | -10.19 | -21.78 | 0.02 | 0.000107 |
| -15.82 | histone cluster 1, H2al | -11.76 | -19.69 | 0.00 | 0.000002 |
| -15.58 | dihydropyrimidinase-like 3 | -11.67 | -19.32 | 0.00 | 0.000001 |
| -15.35 | phosphatidylinositol 3 kinase, regulatory subunit, polypeptide 3 (p55) | -9.26 | -21.03 | 0.03 | 0.000206 |
| -14.90 | histone cluster 1, H2ak | -8.91 | -20.50 | 0.03 | 0.000231 |
| -14.71 | adenomatosis polyposis coli down-regulated 1 | -9.94 | -19.22 | 0.01 | 0.000024 |
| -14.63 | histone cluster 1, H2ah | -9.22 | -19.72 | 0.02 | 0.000100 |
| -14.48 | transmembrane protein 100 | -10.48 | -18.30 | 0.00 | 0.000004 |
| -14.31 | integral membrane protein 2A | -8.05 | -20.14 | 0.05 | 0.000491 |
| -14.18 | 3-hydroxy-3-methylglutaryl-Coenzyme A synthase 2 | -7.25 | -20.60 | 0.08 | 0.001264 |
| -14.14 | histone cluster 1, H2ao | -8.03 | -19.85 | 0.05 | 0.000441 |
| -14.06 | solute carrier family 7 (cationic amino acid transporter, y+ system), member 10 | -8.08 | -19.64 | 0.04 | 0.000377 |
| -13.90 | shadow of prion protein | -6.82 | -20.45 | 0.09 | 0.001740 |
| -13.89 | matrix metallopeptidase 14 (membrane-inserted) | -8.51 | -18.96 | 0.03 | 0.000154 |
| -13.88 | histone cluster 1, H2ai | -10.20 | -17.41 | 0.00 | 0.000002 |
| -13.70 | flavin containing monooxygenase 1 | -7.61 | -19.38 | 0.05 | 0.000555 |
| -13.55 | phosphatidic acid phosphatase type 2A | -8.97 | -17.91 | 0.02 | 0.000038 |
| -13.49 | histone cluster 1, H2an | -7.38 | -19.19 | 0.06 | 0.000653 |
| -13.48 | KN motif and ankyrin repeat domains 1 | -9.57 | -17.21 | 0.01 | 0.000006 |
| -13.43 | tubulin, beta 2a, pseudogene 2; tubulin, beta 2B | -8.77 | -17.86 | 0.02 | 0.000049 |
| -13.27 | histone cluster 1, H2af | -9.02 | -17.32 | 0.01 | 0.000020 |
| -13.16 | similar to NFIL3/E4BP4 transcription factor; nuclear factor, interleukin 3, regulated | -9.19 | -16.97 | 0.01 | 0.000010 |
| -13.11 | epithelial membrane protein 2 | -6.50 | -19.24 | 0.09 | 0.001560 |
| -12.91 | MARCKS-like 1; predicted gene 9106 | -7.43 | -18.07 | 0.04 | 0.000361 |
| -12.89 | transgelin 3 | -6.08 | -19.20 | 0.11 | 0.002233 |
| -12.88 | chromobox homolog 8 (Drosophila Pc class) | -10.02 | -15.66 | 0.00 | 0.000000 |
| -12.75 | potassium channel tetramerisation domain containing 5 | -7.53 | -17.68 | 0.04 | 0.000256 |
| -12.69 | cysteine and glycine-rich protein 2 | -6.95 | -18.07 | 0.06 | 0.000627 |
| -12.59 | guanidinoacetate methyltransferase | -8.24 | -16.74 | 0.02 | 0.000045 |
| -12.54 | tenascin C | -8.31 | -16.59 | 0.01 | 0.000035 |
| -12.51 | phosphofructokinase, platelet | -6.58 | -18.07 | 0.07 | 0.000940 |
| -12.51 | histone cluster 2, H2aa2 | -5.30 | -19.16 | 0.14 | 0.004155 |
| -12.49 | SWI/SNF related, matrix associated, actin dependent regulator of chromatin, subfamily e, member 1 | -7.84 | -16.91 | 0.02 | 0.000099 |
| -12.37 | fatty acid desaturase 2 | -6.76 | -17.64 | 0.06 | 0.000643 |
| -12.19 | growth arrest and DNA-damage-inducible 45 gamma | -6.43 | -17.59 | 0.07 | 0.000905 |
| -12.00 | dephospho-CoA kinase domain containing | -4.92 | -18.56 | 0.15 | 0.004859 |
| -11.98 | RIKEN cDNA E130309F12 gene; predicted gene 12451 | -8.00 | -15.79 | 0.01 | 0.000029 |
| -11.89 | heat shock protein 1 (chaperonin 10) | -5.26 | -18.05 | 0.13 | 0.003253 |
| -11.80 | RIKEN cDNA 4922502B01 gene | -7.24 | -16.14 | 0.03 | 0.000140 |
| -11.79 | myosin, light polypeptide 9, regulatory | -8.50 | -14.96 | 0.00 | 0.000004 |
| -11.72 | histone cluster 2, H2aa1 | -6.79 | -16.39 | 0.04 | 0.000320 |
| -11.60 | RIKEN cDNA D430019H16 gene | -4.78 | -17.92 | 0.15 | 0.004675 |
| -11.51 | cyclin D1 | -6.96 | -15.84 | 0.03 | 0.000175 |
| -11.49 | predicted gene 4322; brain expressed gene 4 | -5.43 | -17.16 | 0.10 | 0.002142 |
| -11.41 | predicted gene 12141; heat shock protein 1 (chaperonin) | -6.26 | -16.28 | 0.05 | 0.000600 |
| -11.41 | bone morphogenetic protein 4 | -6.68 | -15.90 | 0.04 | 0.000277 |
| -11.33 | protein regulator of cytokinesis 1 | -8.93 | -13.67 | 0.00 | 0.000000 |
| -11.31 | microtubule-associated protein 1 light chain 3 alpha | -4.80 | -17.37 | 0.14 | 0.004034 |
| -11.21 | cytochrome P450, family 11, subfamily a, polypeptide 1 | -6.75 | -15.46 | 0.03 | 0.000185 |
| -11.09 | neurocan; similar to Neurocan | -5.69 | -16.18 | 0.07 | 0.001135 |
| -11.06 | lymphocyte antigen 6 complex, locus G6E | -6.37 | -15.52 | 0.04 | 0.000336 |
| -11.06 | glutamate receptor interacting protein 1 | -4.97 | -16.76 | 0.12 | 0.002924 |
| -11.06 | small EDRK-rich factor 1 | -4.67 | -17.02 | 0.14 | 0.004121 |
| -11.03 | PARK2 co-regulated | -7.55 | -14.39 | 0.01 | 0.000016 |
| -11.02 | cytochrome P450, family 51 | -7.10 | -14.77 | 0.02 | 0.000057 |
| -10.96 | protein arginine N-methyltransferase 2 | -5.76 | -15.86 | 0.07 | 0.000906 |
| -10.94 | 5'-nucleotidase, cytosolic III-like | -7.51 | -14.24 | 0.01 | 0.000014 |
| -10.86 | predicted gene 10395; predicted gene 9746; DNA segment, Chr 14, ERATO Doi 449, expressed | -6.99 | -14.57 | 0.02 | 0.000059 |
| -10.82 | synuclein, alpha interacting protein (synphilin) | -8.61 | -12.97 | 0.00 | 0.000000 |
| -10.78 | myeloid-associated differentiation marker | -4.75 | -16.42 | 0.13 | 0.003264 |
| -10.70 | transcription elongation factor A (SII)-like 3 | -5.79 | -15.36 | 0.06 | 0.000685 |
| -10.70 | retinoblastoma binding protein 4 | -4.91 | -16.13 | 0.11 | 0.002548 |
| -10.64 | G protein-coupled receptor 125 | -5.68 | -15.35 | 0.06 | 0.000786 |
| -10.63 | kelch-like 5 (Drosophila) | -6.65 | -14.44 | 0.02 | 0.000097 |
| -10.60 | chromatin modifying protein 4B | -4.57 | -16.26 | 0.13 | 0.003698 |
| -10.51 | collagen, type IV, alpha 2 | -5.21 | -15.51 | 0.09 | 0.001490 |
| -10.44 | PRP19/PSO4 pre-mRNA processing factor 19 homolog (S. cerevisiae) | -5.30 | -15.30 | 0.08 | 0.001213 |
| -10.40 | BCL2-associated X protein | -5.19 | -15.33 | 0.08 | 0.001415 |
| -10.40 | stathmin-like 3 | -5.30 | -15.22 | 0.08 | 0.001177 |
| -10.38 | melanoma antigen, family E, 2 | -5.72 | -14.81 | 0.05 | 0.000551 |
| -10.38 | RIKEN cDNA 1190003M12 gene | -5.20 | -15.27 | 0.08 | 0.001366 |
| -10.31 | serine (or cysteine) peptidase inhibitor, clade H, member 1 | -5.50 | -14.88 | 0.06 | 0.000775 |
| -10.24 | similar to RIKEN cDNA 2310005N03 | -4.81 | -15.37 | 0.11 | 0.002202 |
| -10.24 | phosphatidic acid phosphatase type 2 domain containing 1B | -6.34 | -13.97 | 0.02 | 0.000115 |
| -10.22 | C1q and tumor necrosis factor related protein 4 | -4.18 | -15.88 | 0.15 | 0.004750 |
| -10.19 | histone cluster 2, H2ac | -5.45 | -14.70 | 0.06 | 0.000755 |
| -10.19 | similar to heat shock protein 8; heat shock protein 8 | -4.48 | -15.57 | 0.13 | 0.003299 |
| -10.17 | SRY-box containing gene 19; SRY-box containing gene 4 | -6.10 | -14.06 | 0.03 | 0.000187 |
| -10.10 | NAD(P) dependent steroid dehydrogenase-like | -7.32 | -12.79 | 0.00 | 0.000003 |
| -10.09 | loricrin | -5.79 | -14.20 | 0.04 | 0.000346 |
| -9.99 | low density lipoprotein receptor-related protein associated protein 1 | -6.58 | -13.27 | 0.01 | 0.000035 |
| -9.99 | EGF-like domain 7 | -4.60 | -15.07 | 0.11 | 0.002464 |
| -9.98 | rhomboid, veinlet-like 1 (Drosophila) | -5.36 | -14.37 | 0.06 | 0.000712 |
| -9.93 | cDNA sequence BC030500 | -5.04 | -14.57 | 0.08 | 0.001216 |
| -9.89 | peptidylprolyl isomerase (cyclophilin)-like 6 | -6.30 | -13.35 | 0.02 | 0.000070 |
| -9.81 | protein kinase, interferon inducible double stranded RNA dependent activator | -4.34 | -14.97 | 0.12 | 0.003129 |
| -9.80 | transmembrane protein 141 | -5.36 | -14.03 | 0.05 | 0.000594 |
| -9.74 | phosphate cytidylyltransferase 2, ethanolamine | -4.43 | -14.76 | 0.12 | 0.002682 |
| -9.74 | predicted gene 5617 | -6.05 | -13.29 | 0.02 | 0.000108 |
| -9.74 | olfactomedin-like 3 | -4.04 | -15.10 | 0.14 | 0.004402 |
| -9.72 | diaphanous homolog 3 (Drosophila) | -5.50 | -13.74 | 0.04 | 0.000396 |
| -9.71 | tumor suppressor candidate 1 | -5.39 | -13.83 | 0.05 | 0.000503 |
| -9.64 | RNA binding motif protein 4 | -4.31 | -14.68 | 0.12 | 0.002948 |
| -9.63 | acyl-CoA thioesterase 7 | -5.99 | -13.12 | 0.02 | 0.000102 |
| -9.60 | transmembrane protein 14C | -4.69 | -14.26 | 0.09 | 0.001641 |
| -9.58 | thiosulfate sulfurtransferase, mitochondrial | -4.76 | -14.16 | 0.08 | 0.001426 |
| -9.57 | GRAM domain containing 3 | -6.72 | -12.33 | 0.01 | 0.000007 |
| -9.56 | GIPC PDZ domain containing family, member 1 | -4.02 | -14.77 | 0.14 | 0.004086 |
| -9.53 | Rho guanine nucleotide exchange factor (GEF7) | -6.43 | -12.54 | 0.01 | 0.000021 |
| -9.53 | minichromosome maintenance deficient 6 (MIS5 homolog, S. pombe) (S. cerevisiae) | -6.51 | -12.45 | 0.01 | 0.000015 |
| -9.53 | similar to neuroligin 3; neuroligin 3 | -4.83 | -13.98 | 0.08 | 0.001198 |
| -9.50 | abhydrolase domain containing 1 | -4.50 | -14.23 | 0.10 | 0.002027 |
| -9.48 | predicted gene 5064; cold shock domain containing E1, RNA binding | -4.76 | -13.96 | 0.08 | 0.001300 |
| -9.44 | solute carrier family 46, member 1 | -5.94 | -12.80 | 0.02 | 0.000084 |
| -9.39 | RIKEN cDNA 2310007H09 gene | -3.95 | -14.51 | 0.14 | 0.004066 |
| -9.38 | midkine | -4.81 | -13.73 | 0.07 | 0.001089 |
| -9.36 | phosphoglycerate mutase family member 5 | -4.97 | -13.55 | 0.06 | 0.000796 |
| -9.35 | sphingomyelin phosphodiesterase, acid-like 3A | -4.71 | -13.75 | 0.08 | 0.001259 |
| -9.33 | membrane-associated ring finger (C3HC4) 4 | -3.77 | -14.56 | 0.15 | 0.004943 |
| -9.33 | RIKEN cDNA 1110012D08 gene | -6.65 | -11.93 | 0.00 | 0.000005 |
| -9.32 | histone cluster 2, H3e | -5.80 | -12.70 | 0.02 | 0.000101 |
| -9.31 | nerve growth factor receptor (TNFRSF16) associated protein 1 | -5.79 | -12.70 | 0.02 | 0.000103 |
| -9.30 | energy homeostasis associated | -4.16 | -14.17 | 0.12 | 0.002933 |
| -9.28 | guanine nucleotide binding protein (G protein), beta 2 | -4.69 | -13.65 | 0.08 | 0.001235 |
| -9.26 | transmembrane protein 53 | -5.30 | -13.05 | 0.04 | 0.000341 |
| -9.25 | cyclin-dependent kinase 5 | -5.60 | -12.76 | 0.03 | 0.000159 |
| -9.23 | protein phosphatase 1, regulatory (inhibitor) subunit 11 | -5.43 | -12.88 | 0.03 | 0.000238 |
| -9.21 | serine (or cysteine) peptidase inhibitor, clade B, member 6a | -3.94 | -14.20 | 0.13 | 0.003741 |
| -9.19 | 60S ribosomal protein L13a (Transplantation antigen P198) (Tum-P198 antigen) | -4.13 | -13.99 | 0.12 | 0.002854 |
| -9.19 | neurotensin receptor 2 | -4.83 | -13.35 | 0.07 | 0.000868 |
| -9.16 | solute carrier family 35, member E3; predicted gene 7341 | -4.33 | -13.75 | 0.10 | 0.002062 |
| -9.10 | emopamil binding protein-like | -5.66 | -12.41 | 0.02 | 0.000100 |
| -9.09 | YdjC homolog (bacterial) | -4.97 | -13.02 | 0.05 | 0.000578 |
| -9.07 | family with sequence similarity 20, member A | -4.59 | -13.34 | 0.08 | 0.001222 |
| -9.06 | H2A histone family, member J | -4.19 | -13.68 | 0.11 | 0.002371 |
| -9.04 | LIM and senescent cell antigen like domains 2 | -4.87 | -13.03 | 0.06 | 0.000687 |
| -9.01 | SCAN domain-containing 1 | -4.53 | -13.29 | 0.08 | 0.001286 |
| -9.01 | calpain 5 | -4.43 | -13.37 | 0.09 | 0.001529 |
| -9.01 | TAF10 RNA polymerase II, TATA box binding protein (TBP)-associated factor | -4.82 | -13.01 | 0.06 | 0.000733 |
| -8.99 | vascular endothelial growth factor C | -5.95 | -11.93 | 0.01 | 0.000030 |
| -8.92 | mitochondrial ribosomal protein 63 | -6.34 | -11.44 | 0.01 | 0.000005 |
| -8.91 | trafficking protein particle complex 1 | -6.17 | -11.57 | 0.01 | 0.000010 |
| -8.90 | GM2 ganglioside activator protein | -4.09 | -13.47 | 0.11 | 0.002469 |
| -8.89 | B9 protein domain 1 | -4.05 | -13.50 | 0.11 | 0.002619 |
| -8.89 | PDZ binding kinase | -7.14 | -10.60 | 0.00 | 0.000000 |
| -8.88 | coiled-coil domain containing 23 | -4.88 | -12.72 | 0.05 | 0.000552 |
| -8.87 | prokineticin receptor 2 | -5.75 | -11.88 | 0.02 | 0.000046 |
| -8.86 | transmembrane protein 158 | -4.63 | -12.91 | 0.07 | 0.000914 |
| -8.86 | family with sequence similarity 149, member B | -4.38 | -13.13 | 0.08 | 0.001458 |
| -8.84 | thymine DNA glycosylase | -5.35 | -12.20 | 0.03 | 0.000156 |
| -8.80 | myosin, light polypeptide 6, alkali, smooth muscle and non-muscle | -5.05 | -12.41 | 0.04 | 0.000332 |
| -8.79 | BCS1-like (yeast) | -5.41 | -12.05 | 0.02 | 0.000121 |
| -8.79 | musculin | -5.41 | -12.04 | 0.02 | 0.000118 |
| -8.78 | predicted gene 7172; similar to tubulin, alpha 1; tubulin, alpha 1A | -3.96 | -13.36 | 0.12 | 0.002773 |
| -8.78 | centromere protein V | -4.72 | -12.67 | 0.06 | 0.000695 |
| -8.77 | predicted gene 9731; RIKEN cDNA 1810009O10 gene | -4.81 | -12.58 | 0.05 | 0.000566 |
| -8.76 | RAS-related C3 botulinum substrate 3 | -4.07 | -13.23 | 0.11 | 0.002297 |
| -8.74 | protein arginine N-methyltransferase 1 | -3.78 | -13.44 | 0.13 | 0.003508 |
| -8.69 | monooxygenase, DBH-like 1 | -3.54 | -13.56 | 0.15 | 0.004706 |
| -8.64 | neurocan; similar to Neurocan | -5.26 | -11.90 | 0.03 | 0.000141 |
| -8.62 | phosphodiesterase 6D, cGMP-specific, rod, delta | -5.84 | -11.32 | 0.01 | 0.000018 |
| -8.60 | KH domain containing, RNA binding, signal transduction associated 1 | -5.45 | -11.65 | 0.02 | 0.000073 |
| -8.57 | RIKEN cDNA E130309F12 gene; predicted gene 12451 | -4.77 | -12.23 | 0.05 | 0.000477 |
| -8.56 | neural precursor cell expressed, developmentally down-regulated gene 8 | -3.76 | -13.12 | 0.12 | 0.003184 |
| -8.55 | G protein-coupled receptor 162 | -4.66 | -12.28 | 0.05 | 0.000590 |
| -8.54 | squalene epoxidase | -3.74 | -13.11 | 0.13 | 0.003276 |
| -8.54 | collapsin response mediator protein 1 | -3.74 | -13.09 | 0.13 | 0.003247 |
| -8.52 | WW domain binding protein 1 | -3.63 | -13.16 | 0.13 | 0.003803 |
| -8.52 | peroxisomal biogenesis factor 10 | -5.66 | -11.29 | 0.01 | 0.000027 |
| -8.51 | hematological and neurological expressed sequence 1; predicted gene 3687 | -5.13 | -11.78 | 0.03 | 0.000167 |
| -8.51 | dynactin 5 | -5.58 | -11.34 | 0.01 | 0.000036 |
| -8.50 | RIKEN cDNA E130203B14 gene | -4.09 | -12.70 | 0.09 | 0.001767 |
| -8.50 | SLIT and NTRK-like family, member 1 | -5.29 | -11.60 | 0.02 | 0.000100 |
| -8.49 | lethal, Chr 7, Rinchik 6 | -3.68 | -13.06 | 0.13 | 0.003468 |
| -8.48 | Yip1 interacting factor homolog A (S. cerevisiae) | -5.45 | -11.41 | 0.02 | 0.000055 |
| -8.47 | mitochondrial ribosomal protein L14 | -3.72 | -12.98 | 0.12 | 0.003196 |
| -8.44 | coatomer protein complex subunit alpha | -4.71 | -12.03 | 0.05 | 0.000461 |
| -8.43 | dihydropyrimidinase-like 5 | -4.73 | -11.98 | 0.05 | 0.000427 |
| -8.41 | RIKEN cDNA 3110040N11 gene | -4.12 | -12.51 | 0.09 | 0.001548 |
| -8.38 | similar to Protein disulfide isomerase associated 6; protein disulfide isomerase associated 6 | -4.74 | -11.88 | 0.04 | 0.000389 |
| -8.37 | melanocyte proliferating gene 1 | -4.82 | -11.80 | 0.04 | 0.000315 |
| -8.34 | matrix metallopeptidase 15 | -3.77 | -12.68 | 0.11 | 0.002667 |
| -8.33 | similar to Hypocretin (orexin) receptor 1 | -5.02 | -11.53 | 0.03 | 0.000168 |
| -8.26 | integrator complex subunit 10; similar to integrator complex subunit 10 | -4.47 | -11.90 | 0.06 | 0.000642 |
| -8.25 | mitochondrial ribosomal protein S16; predicted gene 9173 | -5.37 | -11.05 | 0.02 | 0.000042 |
| -8.25 | DALR anticodon binding domain containing 3 | -4.24 | -12.09 | 0.07 | 0.001039 |
| -8.24 | kinesin family member 22 | -4.90 | -11.47 | 0.03 | 0.000201 |
| -8.24 | transmembrane protein 169 | -4.92 | -11.45 | 0.03 | 0.000191 |
| -8.22 | small nuclear ribonucleoprotein polypeptide A | -3.83 | -12.42 | 0.11 | 0.002227 |
| -8.22 | mevalonate kinase; similar to mevalonate kinase | -6.13 | -10.26 | 0.00 | 0.000001 |
| -8.22 | DNA segment, Chr 17, Wayne State University 104, expressed | -4.43 | -11.86 | 0.06 | 0.000665 |
| -8.21 | glycosyltransferase 8 domain containing 2 | -4.32 | -11.95 | 0.06 | 0.000842 |
| -8.20 | FYVE, RhoGEF and PH domain containing 1 | -5.03 | -11.26 | 0.02 | 0.000123 |
| -8.18 | sema domain, immunoglobulin domain, transmembrane and short cytoplasmic domain, 4G | -4.59 | -11.64 | 0.05 | 0.000426 |
| -8.17 | syntaxin 12 | -3.51 | -12.60 | 0.13 | 0.003579 |
| -8.16 | protein phosphatase 4, catalytic subunit | -4.84 | -11.36 | 0.03 | 0.000208 |
| -8.14 | nuclear receptor subfamily 2, group F, member 6 | -4.06 | -12.05 | 0.08 | 0.001355 |
| -8.12 | proteasome (prosome, macropain) 26S subunit, ATPase 3 | -3.60 | -12.43 | 0.12 | 0.003023 |
| -8.10 | ST6 (alpha-N-acetyl-neuraminyl-2,3-beta-galactosyl-1,3)-N-acetylgalactosaminide alpha-2,6-sialyltransferase 6 | -3.29 | -12.68 | 0.15 | 0.004805 |
| -8.10 | single stranded DNA binding protein 4 | -4.18 | -11.86 | 0.07 | 0.001003 |
| -8.09 | RIKEN cDNA 5430437P03 gene | -4.37 | -11.67 | 0.06 | 0.000647 |
| -8.08 | calcium/calmodulin-dependent serine protein kinase (MAGUK family) | -4.52 | -11.50 | 0.05 | 0.000435 |
| -8.07 | alkB, alkylation repair homolog 7 (E. coli) | -4.38 | -11.61 | 0.05 | 0.000608 |
| -8.04 | mitochondrial ribosomal protein L40 | -3.57 | -12.32 | 0.12 | 0.003013 |
| -8.03 | Nucleoside diphosphate kinase B (NDK B) (NDP kinase B) (P18) | -3.80 | -12.08 | 0.10 | 0.001998 |
| -8.03 | branched chain ketoacid dehydrogenase kinase | -3.48 | -12.36 | 0.13 | 0.003401 |
| -8.00 | predicted gene 5217; prune homolog (Drosophila) | -3.39 | -12.39 | 0.13 | 0.003844 |
| -7.97 | deleted in lymphocytic leukemia, 7 | -4.26 | -11.54 | 0.06 | 0.000720 |
| -7.90 | glycerophosphodiester phosphodiesterase domain containing 2 | -4.42 | -11.25 | 0.05 | 0.000433 |
| -7.90 | makorin, ring finger protein, 3 | -4.95 | -10.75 | 0.02 | 0.000084 |
| -7.87 | RIKEN cDNA 2810452K22 gene; similar to cyclin-dependent kinase 2-interacting protein | -3.40 | -12.14 | 0.13 | 0.003496 |
| -7.86 | tumor necrosis factor, alpha-induced protein 8 | -4.46 | -11.14 | 0.04 | 0.000366 |
| -7.83 | ribosomal protein S15a | -3.42 | -12.04 | 0.13 | 0.003263 |
| -7.82 | ankyrin repeat domain 50 | -4.13 | -11.37 | 0.06 | 0.000808 |
| -7.82 | carbohydrate kinase domain containing | -3.67 | -11.79 | 0.10 | 0.002084 |
| -7.80 | family with sequence similarity 132, member A | -3.63 | -11.79 | 0.11 | 0.002231 |
| -7.80 | RIKEN cDNA 2210008F06 gene | -3.93 | -11.50 | 0.08 | 0.001216 |
| -7.79 | mitochondrial ribosomal protein L12 | -4.53 | -10.95 | 0.04 | 0.000274 |
| -7.79 | heparan sulfate 6-O-sulfotransferase 2 | -3.52 | -11.87 | 0.11 | 0.002670 |
| -7.77 | p21 protein (Cdc42/Rac)-activated kinase 4 | -3.77 | -11.60 | 0.09 | 0.001650 |
| -7.74 | translocase of outer mitochondrial membrane 40 homolog-like (yeast) | -4.90 | -10.50 | 0.02 | 0.000072 |
| -7.72 | single-stranded DNA binding protein 2; predicted gene 12470 | -3.26 | -11.97 | 0.14 | 0.003903 |
| -7.71 | zinc finger protein 428 | -4.32 | -10.98 | 0.05 | 0.000424 |
| -7.71 | haloacid dehalogenase-like hydrolase domain containing 3 | -4.53 | -10.78 | 0.03 | 0.000235 |
| -7.70 | similar to Cell division protein kinase 4; cyclin-dependent kinase 4 | -4.56 | -10.73 | 0.03 | 0.000208 |
| -7.68 | UDP-glucose pyrophosphorylase 2 | -3.39 | -11.77 | 0.12 | 0.003068 |
| -7.66 | guanine nucleotide binding protein (G protein), gamma 12 | -3.72 | -11.44 | 0.09 | 0.001644 |
| -7.65 | cytidine 5'-triphosphate synthase | -4.72 | -10.50 | 0.02 | 0.000111 |
| -7.60 | transmembrane and tetratricopeptide repeat containing 4 | -3.43 | -11.60 | 0.12 | 0.002705 |
| -7.58 | spindlin family, member 2 | -4.35 | -10.70 | 0.04 | 0.000316 |
| -7.57 | COMM domain containing 1 | -3.77 | -11.21 | 0.08 | 0.001329 |
| -7.55 | leucine rich repeat containing 7 | -4.29 | -10.70 | 0.04 | 0.000358 |
| -7.55 | histone cluster 2, H3d | -4.83 | -10.19 | 0.02 | 0.000058 |
| -7.54 | RAB7, member RAS oncogene family-like 1 | -4.49 | -10.50 | 0.03 | 0.000192 |
| -7.54 | eukaryotic translation elongation factor 1 epsilon 1 | -4.09 | -10.86 | 0.05 | 0.000604 |
| -7.53 | hypothetical protein LOC100046169; RIKEN cDNA 2810416G20 gene | -4.25 | -10.71 | 0.04 | 0.000395 |
| -7.53 | AFG3(ATPase family gene 3)-like 1 (yeast) | -3.25 | -11.62 | 0.13 | 0.003469 |
| -7.53 | RIKEN cDNA 0610010K14 gene | -3.37 | -11.51 | 0.12 | 0.002823 |
| -7.53 | wingless-related MMTV integration site 7A | -4.44 | -10.51 | 0.03 | 0.000219 |
| -7.52 | PDZ domain containing 11 | -3.31 | -11.55 | 0.12 | 0.003108 |
| -7.49 | Sec61, alpha subunit 2 (S. cerevisiae) | -3.20 | -11.59 | 0.13 | 0.003675 |
| -7.48 | ribosomal protein L31 | -3.75 | -11.08 | 0.08 | 0.001288 |
| -7.47 | acyl-CoA thioesterase 8 | -4.38 | -10.46 | 0.03 | 0.000236 |
| -7.47 | TBC1 domain family, member 7 | -4.64 | -10.21 | 0.02 | 0.000099 |
| -7.46 | ankyrin repeat domain 40 | -3.02 | -11.71 | 0.15 | 0.004817 |
| -7.46 | LIM and cysteine-rich domains 1 | -3.36 | -11.38 | 0.12 | 0.002719 |
| -7.45 | RIKEN cDNA E030049G20 gene | -3.79 | -10.97 | 0.07 | 0.001120 |
| -7.45 | protein phosphatase 2, regulatory subunit B (B56), delta isoform | -3.01 | -11.68 | 0.15 | 0.004849 |
| -7.45 | macrophage migration inhibitory factor | -3.55 | -11.19 | 0.10 | 0.001883 |
| -7.44 | dpy-30 homolog (C. elegans) | -3.01 | -11.66 | 0.15 | 0.004758 |
| -7.43 | 7-dehydrocholesterol reductase | -4.73 | -10.06 | 0.02 | 0.000064 |
| -7.42 | stanniocalcin 1 | -3.25 | -11.41 | 0.13 | 0.003218 |
| -7.40 | similar to Armadillo repeat containing, X-linked 6; armadillo repeat containing, X-linked 6 | -4.30 | -10.40 | 0.04 | 0.000265 |
| -7.40 | lysophosphatidylcholine acyltransferase 3 | -3.33 | -11.30 | 0.12 | 0.002727 |
| -7.39 | RIKEN cDNA 1700016K19 gene | -3.49 | -11.13 | 0.10 | 0.001984 |
| -7.39 | angiopoietin-like 4 | -3.82 | -10.83 | 0.07 | 0.000968 |
| -7.34 | C-type lectin domain family, member l | -3.07 | -11.42 | 0.14 | 0.004047 |
| -7.33 | RIKEN cDNA 1600012H06 gene | -3.82 | -10.71 | 0.07 | 0.000890 |
| -7.31 | transmembrane protein 111 | -3.46 | -11.01 | 0.10 | 0.001975 |
| -7.30 | CD93 antigen | -4.71 | -9.82 | 0.02 | 0.000049 |
| -7.29 | ADP-ribosylation factor-like 6 interacting protein 5 | -3.29 | -11.12 | 0.11 | 0.002665 |
| -7.28 | nucleolar complex associated 4 homolog (S. cerevisiae) | -2.92 | -11.44 | 0.15 | 0.004947 |
| -7.27 | phosphatidic acid phosphatase type 2 domain containing 1A | -4.19 | -10.25 | 0.04 | 0.000298 |
| -7.27 | reticulon 1 | -3.16 | -11.20 | 0.13 | 0.003314 |
| -7.25 | eukaryotic translation initiation factor 4E member 2 | -3.12 | -11.20 | 0.13 | 0.003508 |
| -7.25 | thrombospondin 3 | -4.07 | -10.31 | 0.04 | 0.000408 |
| -7.24 | sphingomyelin phosphodiesterase 1, acid lysosomal | -3.11 | -11.19 | 0.13 | 0.003545 |
| -7.21 | myeloid/lymphoid or mixed-lineage leukemia (trithorax homolog, Drosophila); translocated to, 11 | -3.44 | -10.83 | 0.10 | 0.001846 |
| -7.17 | transmembrane protein 147 | -3.41 | -10.79 | 0.10 | 0.001900 |
| -7.15 | histone cluster 2, H3f | -4.02 | -10.19 | 0.04 | 0.000407 |
| -7.15 | BCL2-associated agonist of cell death | -3.34 | -10.81 | 0.10 | 0.002137 |
| -7.13 | transmembrane protein 183A | -3.60 | -10.54 | 0.08 | 0.001205 |
| -7.13 | ubiquitin associated domain containing 1 | -3.55 | -10.58 | 0.08 | 0.001339 |
| -7.12 | multiple EGF-like-domains 11 | -3.39 | -10.70 | 0.10 | 0.001859 |
| -7.11 | immunoglobulin superfamily, member 9 | -3.00 | -11.04 | 0.13 | 0.003864 |
| -7.10 | TNF receptor associated factor 4 | -4.50 | -9.62 | 0.02 | 0.000067 |
| -7.10 | transmembrane protein 93 | -3.61 | -10.46 | 0.07 | 0.001116 |
| -7.09 | glycogen synthase kinase 3 alpha | -3.37 | -10.66 | 0.10 | 0.001903 |
| -7.08 | mitchondrial ribosomal protein S7 | -3.37 | -10.65 | 0.10 | 0.001888 |
| -7.07 | zinc finger, C4H2 domain containing | -3.57 | -10.45 | 0.08 | 0.001188 |
| -7.06 | insulin-like growth factor binding protein-like 1 | -5.68 | -8.42 | 0.00 | 0.000000 |
| -7.06 | HSPA (heat shock 70kDa) binding protein, cytoplasmic cochaperone 1 | -2.97 | -10.97 | 0.14 | 0.003968 |
| -7.05 | NADH dehydrogenase (ubiquinone) flavoprotein 3 | -3.58 | -10.38 | 0.07 | 0.001109 |
| -7.02 | glutathione peroxidase 7 | -4.00 | -9.94 | 0.04 | 0.000336 |
| -6.99 | tubulin, beta 5 | -2.98 | -10.82 | 0.13 | 0.003637 |
| -6.96 | retinoic acid induced 12 | -4.20 | -9.63 | 0.03 | 0.000152 |
| -6.95 | ADAMTS-like 2 | -3.38 | -10.40 | 0.09 | 0.001602 |
| -6.95 | hyaluronan and proteoglycan link protein 1 | -4.05 | -9.76 | 0.04 | 0.000256 |
| -6.93 | RIKEN cDNA B230311B06 gene | -3.76 | -10.00 | 0.05 | 0.000603 |
| -6.92 | mitochondrial GTPase 1 homolog (S. cerevisiae) | -3.13 | -10.56 | 0.11 | 0.002593 |
| -6.90 | transmembrane protein 33 | -2.99 | -10.66 | 0.13 | 0.003391 |
| -6.90 | exoribonuclease 3 | -2.79 | -10.83 | 0.15 | 0.004796 |
| -6.90 | cytochrome c-1 | -3.62 | -10.06 | 0.06 | 0.000839 |
| -6.90 | proline rich 15 | -3.31 | -10.35 | 0.09 | 0.001753 |
| -6.89 | inositol polyphosphate-4-phosphatase, type I | -4.18 | -9.53 | 0.03 | 0.000143 |
| -6.89 | epsin 1 | -3.30 | -10.34 | 0.09 | 0.001771 |
| -6.89 | uroporphyrinogen III synthase | -3.62 | -10.04 | 0.06 | 0.000812 |
| -6.86 | ubiquitin-like 4 | -2.84 | -10.71 | 0.14 | 0.004223 |
| -6.84 | adaptor-related protein complex 2, sigma 1 subunit | -3.33 | -10.23 | 0.09 | 0.001587 |
| -6.84 | short chain dehydrogenase/reductase family 39U, member 1 | -3.61 | -9.95 | 0.06 | 0.000779 |
| -6.83 | REX4, RNA exonuclease 4 homolog (S. cerevisiae) | -3.06 | -10.46 | 0.12 | 0.002755 |
| -6.81 | zinc finger, CCCH-type with G patch domain; Lck interacting transmembrane adaptor 1 | -3.26 | -10.23 | 0.10 | 0.001788 |
| -6.80 | leucine rich repeat containing 51 | -2.89 | -10.55 | 0.13 | 0.003720 |
| -6.80 | ras homolog gene family, member T1 | -2.85 | -10.59 | 0.14 | 0.004031 |
| -6.79 | replication factor C (activator 1) 2 | -3.25 | -10.21 | 0.10 | 0.001798 |
| -6.78 | vesicle transport through interaction with t-SNAREs homolog 1A (yeast) | -2.73 | -10.66 | 0.15 | 0.004830 |
| -6.77 | endothelin receptor type A | -3.22 | -10.20 | 0.10 | 0.001891 |
| -6.74 | thioesterase superfamily member 4 | -2.86 | -10.46 | 0.13 | 0.003745 |
| -6.73 | RIKEN cDNA 4933403G14 gene | -2.70 | -10.59 | 0.15 | 0.004911 |
| -6.73 | crystallin, zeta | -3.46 | -9.89 | 0.07 | 0.001013 |
| -6.73 | signal recognition particle receptor, B subunit | -3.02 | -10.29 | 0.12 | 0.002711 |
| -6.72 | male-specific lethal 1 homolog (Drosophila) | -4.22 | -9.16 | 0.02 | 0.000081 |
| -6.72 | vang-like 2 (van gogh, Drosophila) | -3.81 | -9.54 | 0.04 | 0.000359 |
| -6.72 | epidermal growth factor receptor | -3.28 | -10.03 | 0.09 | 0.001524 |
| -6.69 | sema domain, transmembrane domain (TM), and cytoplasmic domain, (semaphorin) 6B | -3.06 | -10.17 | 0.11 | 0.002401 |
| -6.68 | mitochondrial ribosomal protein L43 | -2.73 | -10.47 | 0.14 | 0.004494 |
| -6.65 | predicted gene 4691;similar to Glyceraldehyde-3-phosphate dehydrogenase (GAPDH) | -3.19 | -9.99 | 0.09 | 0.001747 |
| -6.64 | chromatin modifying protein 6 | -3.74 | -9.46 | 0.04 | 0.000395 |
| -6.63 | histone deacetylase 8 | -3.85 | -9.34 | 0.04 | 0.000265 |
| -6.63 | Coenzyme A synthase | -2.90 | -10.22 | 0.12 | 0.003203 |
| -6.62 | reticulon 3 | -2.93 | -10.18 | 0.12 | 0.003006 |
| -6.60 | RIKEN cDNA 2610024G14 gene | -2.95 | -10.12 | 0.12 | 0.002794 |
| -6.60 | RIKEN cDNA 0610038F07 gene | -3.70 | -9.41 | 0.05 | 0.000416 |
| -6.56 | expressed sequence AU022252 | -3.57 | -9.47 | 0.05 | 0.000582 |
| -6.54 | dehydrogenase/reductase (SDR family) member 7B | -3.57 | -9.41 | 0.05 | 0.000552 |
| -6.53 | SRY-box containing gene 2 | -3.27 | -9.68 | 0.08 | 0.001262 |
| -6.51 | TEA domain family member 2 | -3.67 | -9.27 | 0.04 | 0.000389 |
| -6.51 | family with sequence similarity 96, member B | -2.89 | -10.00 | 0.12 | 0.002920 |
| -6.50 | CKLF-like MARVEL transmembrane domain containing 7 | -2.97 | -9.90 | 0.11 | 0.002433 |
| -6.50 | leucine zipper and CTNNBIP1 domain containing | -4.05 | -8.89 | 0.02 | 0.000092 |
| -6.50 | integrin linked kinase; predicted gene 6263 | -2.61 | -10.24 | 0.15 | 0.004944 |
| -6.49 | acetyl-Coenzyme A acetyltransferase 3 | -4.08 | -8.85 | 0.02 | 0.000079 |
| -6.46 | tumor-suppressing subchromosomal transferable fragment 4 | -3.56 | -9.27 | 0.05 | 0.000499 |
| -6.45 | CDC16 cell division cycle 16 homolog (S. cerevisiae) | -3.49 | -9.32 | 0.05 | 0.000615 |
| -6.43 | progestin and adipoQ receptor family member IX | -3.32 | -9.45 | 0.07 | 0.000973 |
| -6.43 | adaptor protein complex AP-1, gamma 2 subunit | -3.38 | -9.39 | 0.06 | 0.000820 |
| -6.43 | predicted gene 715 | -4.24 | -8.56 | 0.01 | 0.000030 |
| -6.42 | gamma-glutamyltransferase 7 | -2.59 | -10.11 | 0.15 | 0.004827 |
| -6.42 | predicted gene 5243; NOP56 ribonucleoprotein homolog (yeast) | -2.75 | -9.94 | 0.13 | 0.003532 |
| -6.41 | family with sequence similarity 128, member B | -2.65 | -10.03 | 0.14 | 0.004276 |
| -6.38 | nitrogen fixation gene 1 (S. cerevisiae); similar to Nitrogen fixation gene 1 (S. cerevisiae) | -3.43 | -9.25 | 0.06 | 0.000658 |
| -6.37 | ets variant gene 4 (E1A enhancer binding protein, E1AF) | -2.71 | -9.89 | 0.13 | 0.003671 |
| -6.37 | kelch-like 6 (Drosophila) | -4.05 | -8.62 | 0.02 | 0.000061 |
| -6.36 | predicted gene 2893; electron transferring flavoprotein, alpha polypeptide | -2.63 | -9.95 | 0.14 | 0.004226 |
| -6.35 | COMM domain containing 9 | -3.44 | -9.17 | 0.05 | 0.000598 |
| -6.34 | peptidylprolyl isomerase (cyclophilin)-like 3 | -2.75 | -9.79 | 0.13 | 0.003295 |
| -6.33 | calcyon neuron-specific vesicular protein | -2.88 | -9.66 | 0.11 | 0.002474 |
| -6.33 | ADP-ribosylation factor interacting protein 2 | -2.95 | -9.59 | 0.10 | 0.002126 |
| -6.33 | RIKEN cDNA 5430435G22 gene | -3.91 | -8.69 | 0.02 | 0.000104 |
| -6.33 | putative homeodomain transcription factor 1 | -2.75 | -9.78 | 0.13 | 0.003296 |
| -6.32 | discoidin, CUB and LCCL domain containing 1 | -3.60 | -8.97 | 0.04 | 0.000341 |
| -6.31 | transforming growth factor beta regulated gene 1 | -3.21 | -9.32 | 0.07 | 0.001087 |
| -6.28 | ash2 (absent, small, or homeotic)-like (Drosophila) | -2.64 | -9.78 | 0.14 | 0.003888 |
| -6.27 | testis specific gene A14 | -3.64 | -8.84 | 0.04 | 0.000267 |
| -6.27 | cDNA sequence BC003940 | -2.97 | -9.45 | 0.10 | 0.001895 |
| -6.27 | hexamthylene bis-acetamide inducible 2 | -2.57 | -9.83 | 0.14 | 0.004464 |
| -6.25 | zinc finger, C3HC type 1 | -3.15 | -9.25 | 0.08 | 0.001186 |
| -6.25 | LIM and SH3 protein 1 | -2.59 | -9.77 | 0.14 | 0.004206 |
| -6.23 | thymine DNA glycosylase; predicted gene 5597; predicted gene 9855; predicted gene 5806 | -2.88 | -9.47 | 0.11 | 0.002243 |
| -6.22 | transmembrane protein 70 | -2.67 | -9.64 | 0.13 | 0.003515 |
| -6.21 | hyperpolarization-activated, cyclic nucleotide-gated K+ 3 | -2.83 | -9.48 | 0.11 | 0.002485 |
| -6.21 | myelin protein zero-like 1 | -4.03 | -8.33 | 0.02 | 0.000041 |
| -6.20 | NADH dehydrogenase (ubiquinone) 1 beta subcomplex, 6 | -3.18 | -9.13 | 0.07 | 0.001007 |
| -6.20 | expressed sequence R74862 | -3.13 | -9.18 | 0.07 | 0.001161 |
| -6.15 | zinc finger protein 41 | -3.57 | -8.67 | 0.04 | 0.000267 |
| -6.15 | endothelin receptor type B | -3.45 | -8.77 | 0.04 | 0.000405 |
| -6.12 | predicted gene 3599; solute carrier family 38, member 7 | -2.94 | -9.20 | 0.09 | 0.001731 |
| -6.12 | erythrocyte protein band 4.1-like 4a | -3.55 | -8.62 | 0.04 | 0.000263 |
| -6.11 | peroxisomal biogenesis factor 16 | -3.54 | -8.61 | 0.04 | 0.000268 |
| -6.10 | gene trap ROSA b-geo 22 | -3.12 | -9.00 | 0.07 | 0.001049 |
| -6.10 | tubulin tyrosine ligase-like family, member 7 | -3.39 | -8.74 | 0.05 | 0.000447 |
| -6.10 | coiled-coil-helix-coiled-coil-helix domain containing 8 | -3.76 | -8.37 | 0.02 | 0.000104 |
| -6.10 | CLPTM1-like | -3.14 | -8.97 | 0.07 | 0.000984 |
| -6.07 | solute carrier family 25 (mitochondrial carrier, brain), member 14 | -2.52 | -9.50 | 0.14 | 0.004142 |
| -6.05 | protocadherin 12 | -4.10 | -7.96 | 0.01 | 0.000016 |
| -6.04 | small nuclear ribonucleoprotein 35 (U11/U12) | -2.74 | -9.24 | 0.11 | 0.002557 |
| -6.02 | family with sequence similarity 123, member C | -2.61 | -9.30 | 0.13 | 0.003275 |
| -6.00 | COP9 (constitutive photomorphogenic) homolog, subunit 5 (Arabidopsis thaliana) | -3.78 | -8.18 | 0.02 | 0.000075 |
| -5.97 | histone cluster 2, H2ab | -3.76 | -8.13 | 0.02 | 0.000075 |
| -5.96 | vacuolar protein sorting 53 (yeast) | -2.80 | -9.02 | 0.10 | 0.002009 |
| -5.95 | RAB, member of RAS oncogene family-like 5 | -2.44 | -9.33 | 0.14 | 0.004384 |
| -5.93 | RIKEN cDNA 1700007K13 gene | -2.46 | -9.29 | 0.14 | 0.004207 |
| -5.92 | proteasome (prosome, macropain) assembly chaperone 3 | -2.47 | -9.24 | 0.14 | 0.004038 |
| -5.91 | transmembrane and coiled-coil domains 3 | -3.01 | -8.73 | 0.07 | 0.001085 |
| -5.91 | multiple PDZ domain protein | -2.71 | -9.00 | 0.11 | 0.002353 |
| -5.88 | tetraspanin 9 | -2.76 | -8.90 | 0.10 | 0.002019 |
| -5.87 | RIKEN cDNA 1810043H04 gene | -2.60 | -9.04 | 0.12 | 0.002901 |
| -5.84 | phosducin-like | -2.83 | -8.76 | 0.09 | 0.001598 |
| -5.82 | carbohydrate (chondroitin 6/keratan) sulfotransferase 3 | -3.49 | -8.09 | 0.03 | 0.000162 |
| -5.82 | intraflagellar transport 122 homolog (Chlamydomonas) | -2.39 | -9.12 | 0.14 | 0.004352 |
| -5.82 | ATP synthase, H+ transporting, mitochondrial F0 complex, subunit s | -2.45 | -9.07 | 0.13 | 0.003848 |
| -5.80 | major facilitator superfamily domain containing 3 | -2.90 | -8.60 | 0.08 | 0.001233 |
| -5.79 | a disintegrin-like and metallopeptidase (reprolysin type) with thrombospondin type 1 motif, 18 | -3.75 | -7.78 | 0.02 | 0.000042 |
| -5.79 | family with sequence similarity 110, member A | -2.61 | -8.86 | 0.11 | 0.002592 |
| -5.78 | reticulon 4 interacting protein 1 | -2.84 | -8.64 | 0.08 | 0.001456 |
| -5.77 | DEAH (Asp-Glu-Ala-His) box polypeptide 29 | -2.44 | -8.99 | 0.13 | 0.003792 |
| -5.77 | metallo-beta-lactamase domain containing 1 | -2.72 | -8.72 | 0.10 | 0.001934 |
| -5.76 | RIKEN cDNA 5730437N04 gene | -2.34 | -9.05 | 0.14 | 0.004546 |
| -5.75 | SAR1 gene homolog B (S. cerevisiae) | -2.59 | -8.81 | 0.11 | 0.002636 |
| -5.74 | E26 avian leukemia oncogene 1, 5' domain | -2.62 | -8.76 | 0.11 | 0.002436 |
| -5.73 | transmembrane 7 superfamily member 2 | -2.65 | -8.71 | 0.11 | 0.002229 |
| -5.70 | RIKEN cDNA 1700052K11 gene | -2.67 | -8.63 | 0.10 | 0.002030 |
| -5.69 | RIKEN cDNA 5330417C22 gene | -2.95 | -8.35 | 0.07 | 0.000918 |
| -5.64 | solute carrier family 25 (mitochondrial carrier oxoglutarate carrier), member 11 | -2.36 | -8.81 | 0.14 | 0.003983 |
| -5.63 | lon peptidase 1, mitochondrial | -2.91 | -8.27 | 0.07 | 0.000941 |
| -5.62 | phosphatidylserine synthase 2 | -2.94 | -8.22 | 0.06 | 0.000823 |
| -5.59 | CKLF-like MARVEL transmembrane domain containing 8 | -2.37 | -8.71 | 0.13 | 0.003709 |
| -5.57 | L antigen family, member 3; predicted gene 6723; L antigen family, member 3 pseudogene | -2.43 | -8.61 | 0.12 | 0.003185 |
| -5.56 | integrator complex subunit 10; similar to integrator complex subunit 10 | -2.56 | -8.47 | 0.11 | 0.002298 |
| -5.55 | RIKEN cDNA 9430038I01 gene | -2.85 | -8.16 | 0.07 | 0.000976 |
| -5.54 | HEAT repeat containing 1 | -3.09 | -7.93 | 0.05 | 0.000421 |
| -5.54 | nudix (nucleoside diphosphate linked moiety X)-type motif 5 | -2.20 | -8.76 | 0.15 | 0.005064 |
| -5.53 | thiamine triphosphatase | -2.33 | -8.63 | 0.13 | 0.003819 |
| -5.51 | SUMO/sentrin specific peptidase 3 | -2.27 | -8.64 | 0.14 | 0.004290 |
| -5.51 | histone cluster 2, H2be | -2.43 | -8.48 | 0.12 | 0.002933 |
| -5.50 | transmembrane protein 107 | -2.65 | -8.27 | 0.09 | 0.001675 |
| -5.50 | golgi SNAP receptor complex member 2 | -2.22 | -8.67 | 0.15 | 0.004685 |
| -5.49 | heat shock factor binding protein 1 | -2.19 | -8.68 | 0.15 | 0.004944 |
| -5.47 | ATP synthase mitochondrial F1 complex assembly factor 2 | -2.57 | -8.28 | 0.10 | 0.001982 |
| -5.46 | RIKEN cDNA 2310008H04 gene | -3.03 | -7.83 | 0.05 | 0.000447 |
| -5.44 | stromal antigen 3 | -2.84 | -7.97 | 0.07 | 0.000858 |
| -5.42 | poly-U binding splicing factor 60 | -2.17 | -8.57 | 0.15 | 0.004871 |
| -5.38 | lysophospholipase-like 1 | -2.73 | -7.96 | 0.07 | 0.001091 |
| -5.36 | galactose-4-epimerase, UDP | -2.33 | -8.31 | 0.13 | 0.003262 |
| -5.35 | melanoma cell adhesion molecule | -2.64 | -7.99 | 0.08 | 0.001385 |
| -5.34 | non-SMC condensin I complex, subunit H | -3.26 | -7.37 | 0.02 | 0.000119 |
| -5.31 | RIKEN cDNA A930005I04 gene | -2.56 | -7.98 | 0.09 | 0.001632 |
| -5.31 | RIKEN cDNA 2310033P09 gene | -2.19 | -8.33 | 0.14 | 0.004226 |
| -5.29 | acid phosphatase 2, lysosomal | -2.58 | -7.92 | 0.09 | 0.001489 |
| -5.29 | REX2, RNA exonuclease 2 homolog (S. cerevisiae) | -2.44 | -8.06 | 0.11 | 0.002263 |
| -5.26 | transmembrane protein 63c | -3.26 | -7.22 | 0.02 | 0.000096 |
| -5.25 | mitochondrial ribosomal protein L15 | -2.74 | -7.71 | 0.07 | 0.000860 |
| -5.25 | similar to Manbal protein; mannosidase, beta A, lysosomal-like | -2.78 | -7.66 | 0.06 | 0.000733 |
| -5.24 | 3-oxoacid CoA transferase 1 | -2.17 | -8.22 | 0.14 | 0.004174 |
| -5.23 | aquaporin 9 | -2.19 | -8.18 | 0.14 | 0.003913 |
| -5.23 | proline-rich Gla (G-carboxyglutamic acid) polypeptide 2 | -2.15 | -8.22 | 0.14 | 0.004344 |
| -5.23 | similar to ADP-ribosylation factor-like protein 7; ADP-ribosylation factor-like 4C | -2.09 | -8.27 | 0.15 | 0.004911 |
| -5.21 | similar to Dopachrome tautomerase; dopachrome tautomerase | -2.34 | -7.99 | 0.11 | 0.002622 |
| -5.18 | cyclin-dependent kinase inhibitor 2C (p18, inhibits CDK4) | -2.08 | -8.18 | 0.15 | 0.004800 |
| -5.14 | protocadherin beta 10 | -2.35 | -7.86 | 0.11 | 0.002396 |
| -5.11 | KN motif and ankyrin repeat domains 3 | -2.12 | -8.00 | 0.14 | 0.004057 |
| -5.11 | neighbor of Brca1 gene 1 | -2.64 | -7.50 | 0.07 | 0.000908 |
| -5.09 | ubiquitin-like, containing PHD and RING finger domains, 1 | -3.24 | -6.91 | 0.02 | 0.000059 |
| -5.09 | asparaginase like 1 | -2.24 | -7.86 | 0.12 | 0.002969 |
| -5.07 | CD200 antigen; similar to MRC OX-2 antigen homolog | -2.02 | -8.04 | 0.15 | 0.005042 |
| -5.06 | mitogen-activated protein kinase kinase 6 | -2.29 | -7.76 | 0.11 | 0.002564 |
| -5.06 | platelet-derived growth factor receptor-like | -2.98 | -7.10 | 0.03 | 0.000208 |
| -5.02 | cAMP responsive element modulator | -2.01 | -7.93 | 0.15 | 0.004811 |
| -4.95 | RIKEN cDNA 9330171B17 gene | -2.17 | -7.65 | 0.12 | 0.003055 |
| -4.94 | arachidonate 12-lipoxygenase | -2.28 | -7.53 | 0.11 | 0.002241 |
| -4.88 | caspase 6 | -2.28 | -7.40 | 0.10 | 0.002028 |
| -4.86 | ATPase, Ca++ transporting, ubiquitous | -2.05 | -7.58 | 0.13 | 0.003751 |
| -4.85 | CKLF-like MARVEL transmembrane domain containing 4 | -2.51 | -7.12 | 0.07 | 0.000889 |
| -4.79 | predicted gene 5626; stomatin (Epb7.2)-like 2 | -2.46 | -7.06 | 0.07 | 0.000979 |
| -4.73 | cullin 4A | -2.59 | -6.83 | 0.05 | 0.000507 |
| -4.70 | retinaldehyde binding protein 1-like 1 | -2.74 | -6.63 | 0.03 | 0.000241 |
| -4.69 | DNA segment, Chr 2, Wayne State University 81, expressed | -2.02 | -7.29 | 0.13 | 0.003355 |
| -4.69 | ATP synthase, H+ transporting, mitochondrial F0 complex, subunit s | -2.37 | -6.96 | 0.07 | 0.001127 |
| -4.68 | nuclear autoantigenic sperm protein (histone-binding) | -1.85 | -7.42 | 0.15 | 0.005084 |
| -4.66 | phosphatidylinositol glycan anchor biosynthesis, class F | -2.03 | -7.21 | 0.12 | 0.003110 |
| -4.65 | EMI domain containing 1 | -2.67 | -6.59 | 0.04 | 0.000288 |
| -4.63 | lin-7 homolog A (C. elegans) | -1.85 | -7.34 | 0.15 | 0.004924 |
| -4.60 | unc-119 homolog (C. elegans) | -1.90 | -7.22 | 0.14 | 0.004143 |
| -4.55 | RIKEN cDNA 9030624J02 gene | -2.04 | -7.00 | 0.11 | 0.002641 |
| -4.54 | cysteinyl-tRNA synthetase 2 (mitochondrial)(putative) | -1.84 | -7.18 | 0.15 | 0.004622 |
| -4.47 | solute carrier family 9 (sodium/hydrogen exchanger), member 6 | -2.36 | -6.54 | 0.06 | 0.000744 |
| -4.47 | myosin X | -2.17 | -6.72 | 0.09 | 0.001545 |
| -4.41 | pentraxin related gene | -2.51 | -6.27 | 0.04 | 0.000323 |
| -4.38 | transcription elongation factor B (SIII), polypeptide 2 | -1.84 | -6.86 | 0.13 | 0.003843 |
| -4.36 | oligodendrocyte myelin glycoprotein | -2.34 | -6.34 | 0.06 | 0.000640 |
| -4.35 | cyclin D binding myb-like transcription factor 1 | -1.81 | -6.83 | 0.14 | 0.004027 |
| -4.32 | similar to Ftsj homolog; FtsJ homolog 1 (E. coli) | -2.11 | -6.48 | 0.08 | 0.001459 |
| -4.31 | PR domain containing 10 | -1.98 | -6.58 | 0.11 | 0.002253 |
| -4.28 | LYR motif containing 2 | -1.94 | -6.57 | 0.11 | 0.002501 |
| -4.26 | tubulin-specific chaperone C | -1.81 | -6.64 | 0.13 | 0.003540 |
| -4.25 | zinc finger and BTB domain containing 34 | -2.17 | -6.29 | 0.07 | 0.001019 |
| -4.23 | serine/threonine kinase 36 (fused homolog, Drosophila) | -2.21 | -6.20 | 0.06 | 0.000802 |
| -4.21 | BRCA1/BRCA2-containing complex, subunit 3 | -2.31 | -6.09 | 0.05 | 0.000509 |
| -4.19 | kelch-like 23 (Drosophila) | -1.94 | -6.39 | 0.10 | 0.002151 |
| -4.16 | phosphatidylinositol glycan anchor biosynthesis, class B | -1.94 | -6.33 | 0.10 | 0.002050 |
| -4.15 | catenin (cadherin associated protein), alpha-like 1 | -2.11 | -6.15 | 0.07 | 0.001065 |
| -4.12 | leucine rich repeat containing 56 | -1.91 | -6.28 | 0.10 | 0.002142 |
| -4.06 | CD97 antigen | -1.76 | -6.31 | 0.12 | 0.003177 |
| -4.04 | RIKEN cDNA 2310075K07 gene | -2.11 | -5.94 | 0.06 | 0.000832 |
| -4.04 | forkhead box S1 | -2.32 | -5.72 | 0.04 | 0.000277 |
| -4.03 | similar to Hypocretin (orexin) receptor 1; hypocretin (orexin) receptor 1 | -2.24 | -5.79 | 0.05 | 0.000433 |
| -4.02 | nuclear factor of activated T-cells, cytoplasmic, calcineurin-dependent 1 | -1.98 | -6.03 | 0.08 | 0.001389 |
| -3.98 | WAP four-disulfide core domain 3 | -1.85 | -6.06 | 0.10 | 0.002049 |
| -3.97 | palmdelphin; similar to palmdelphin | -1.65 | -6.24 | 0.14 | 0.004051 |
| -3.95 | solute carrier family 25 (mitochondrial carrier, citrate transporter), member 1 | -1.90 | -5.95 | 0.09 | 0.001596 |
| -3.92 | START domain containing 9 | -1.61 | -6.18 | 0.14 | 0.004198 |
| -3.91 | aldo-keto reductase family 7, member A5 (aflatoxin aldehyde reductase) | -1.73 | -6.03 | 0.12 | 0.002796 |
| -3.90 | protein phosphatase 1H (PP2C domain containing) | -1.61 | -6.13 | 0.14 | 0.004132 |
| -3.89 | Smith-Magenis syndrome chromosome region, candidate 7 homolog (human) | -1.91 | -5.83 | 0.08 | 0.001390 |
| -3.86 | nuclear receptor subfamily 0, group B, member 1 | -1.87 | -5.81 | 0.09 | 0.001550 |
| -3.86 | biogenesis of lysosome-related organelles complex-1, subunit 3 | -1.84 | -5.83 | 0.09 | 0.001696 |
| -3.83 | FXYD domain-containing ion transport regulator 2 | -1.56 | -6.05 | 0.14 | 0.004413 |
| -3.82 | proprotein convertase subtilisin/kexin type 5 | -1.69 | -5.90 | 0.12 | 0.002857 |
| -3.81 | coiled-coil domain containing 87 | -1.93 | -5.66 | 0.07 | 0.001094 |
| -3.78 | artemin | -2.46 | -5.08 | 0.01 | 0.000035 |
| -3.77 | extracellular proteinase inhibitor | -2.01 | -5.50 | 0.06 | 0.000664 |
| -3.74 | RAB13, member RAS oncogene family | -1.96 | -5.50 | 0.06 | 0.000801 |
| -3.74 | opsin 1 (cone pigments), medium-wave-sensitive (color blindness, deutan) | -1.71 | -5.73 | 0.11 | 0.002342 |
| -3.72 | RIKEN cDNA C330016O10 gene | -1.61 | -5.79 | 0.12 | 0.003177 |
| -3.71 | ATP-binding cassette, sub-family D (ALD), member 4 | -1.53 | -5.85 | 0.14 | 0.004160 |
| -3.67 | RIKEN cDNA 9130017K11 gene | -1.82 | -5.49 | 0.08 | 0.001291 |
| -3.67 | cell division cycle associated 8 | -1.46 | -5.83 | 0.15 | 0.004926 |
| -3.67 | RIKEN cDNA 4632404H12 gene | -1.61 | -5.69 | 0.12 | 0.003011 |
| -3.65 | SYS1 Golgi-localized integral membrane protein homolog (S. cerevisiae) | -1.53 | -5.72 | 0.13 | 0.003759 |
| -3.63 | RIKEN cDNA 1700054O13 gene | -1.51 | -5.71 | 0.14 | 0.004027 |
| -3.61 | potassium channel tetramerisation domain containing 20; predicted gene 8280 | -1.66 | -5.53 | 0.11 | 0.002230 |
| -3.61 | fatty acid binding protein 7, brain | -1.74 | -5.45 | 0.09 | 0.001606 |
| -3.57 | RNA binding motif protein 10; predicted gene 12799 | -1.85 | -5.26 | 0.07 | 0.000861 |
| -3.55 | cytochrome c oxidase, subunit VI a, polypeptide 1; predicted gene 7795 | -1.60 | -5.47 | 0.11 | 0.002503 |
| -3.55 | fatty acyl CoA reductase 1 | -1.55 | -5.51 | 0.12 | 0.003031 |
| -3.54 | baculoviral IAP repeat-containing 5 | -1.87 | -5.19 | 0.06 | 0.000739 |
| -3.54 | fuzzy homolog (Drosophila) | -1.46 | -5.57 | 0.14 | 0.004072 |
| -3.54 | N-acetyltransferase 11 | -1.65 | -5.38 | 0.10 | 0.001989 |
| -3.51 | UDP-Gal:betaGlcNAc beta 1,3-galactosyltransferase, polypeptide 4 | -1.50 | -5.49 | 0.13 | 0.003472 |
| -3.50 | heterogeneous nuclear ribonucleoprotein K; predicted gene 7964 | -1.57 | -5.40 | 0.11 | 0.002576 |
| -3.50 | complexin 4 | -1.39 | -5.56 | 0.15 | 0.004888 |
| -3.49 | shugoshin-like 1 (S. pombe) | -1.53 | -5.41 | 0.12 | 0.002949 |
| -3.48 | HIG1 domain family, member 1B | -1.66 | -5.26 | 0.09 | 0.001681 |
| -3.46 | transmembrane protein 143 | -1.57 | -5.31 | 0.11 | 0.002374 |
| -3.45 | topoisomerase (DNA) II alpha | -1.60 | -5.26 | 0.10 | 0.002076 |
| -3.44 | E74-like factor 5 | -1.38 | -5.45 | 0.15 | 0.004693 |
| -3.38 | WD repeat domain 8 | -1.42 | -5.30 | 0.13 | 0.003692 |
| -3.30 | Rho GTPase activating protein 17 | -1.71 | -4.88 | 0.07 | 0.000896 |
| -3.28 | sialidase 4 | -1.54 | -4.99 | 0.10 | 0.001919 |
| -3.23 | histone cluster 1, H4b | -1.36 | -5.06 | 0.13 | 0.003733 |
| -3.21 | RNA binding motif protein 4B | -1.59 | -4.81 | 0.08 | 0.001305 |
| -3.21 | antigen identified by monoclonal antibody Ki 67 | -1.65 | -4.76 | 0.07 | 0.000952 |
| -3.18 | myosin XVIIIb | -1.25 | -5.08 | 0.15 | 0.005096 |
| -3.15 | gamma-glutamyl carboxylase | -1.49 | -4.79 | 0.10 | 0.001834 |
| -3.08 | cell division cycle associated 2 | -1.51 | -4.63 | 0.08 | 0.001406 |
| -3.07 | serine (or cysteine) peptidase inhibitor, clade A, member 5 | -1.81 | -4.31 | 0.03 | 0.000191 |
| -3.06 | RIKEN cDNA E130006D01 gene | -1.30 | -4.79 | 0.13 | 0.003486 |
| -3.04 | methionine aminopeptidase 2 | -1.32 | -4.74 | 0.12 | 0.003147 |
| -3.02 | similar to mKIAA3002 protein; similar to ROSBIN; rosbin, round spermatid basic protein 1 | -1.31 | -4.69 | 0.12 | 0.003060 |
| -2.96 | NIMA (never in mitosis gene a)-related expressed kinase 4 | -1.42 | -4.47 | 0.09 | 0.001588 |
| -2.95 | NADH dehydrogenase (ubiquinone) 1, alpha/beta subcomplex, 1; predicted gene 4459 | -1.17 | -4.71 | 0.15 | 0.004964 |
| -2.92 | olfactory receptor 559 | -1.56 | -4.26 | 0.06 | 0.000626 |
| -2.88 | leishmanolysin-like (metallopeptidase M8 family) | -1.19 | -4.54 | 0.14 | 0.004040 |
| -2.85 | secretin receptor; similar to Sctr protein | -1.23 | -4.44 | 0.13 | 0.003216 |
| -2.80 | exosome component 2 | -1.26 | -4.31 | 0.11 | 0.002526 |
| -2.79 | peroxisomal biogenesis factor 13 | -1.34 | -4.23 | 0.09 | 0.001633 |
| -2.78 | echinoderm microtubule associated protein like 1 | -1.29 | -4.25 | 0.10 | 0.002040 |
| -2.76 | taste receptor, type 2, member 131 | -1.15 | -4.34 | 0.13 | 0.003837 |
| -2.76 | beta-1,4-N-acetyl-galactosaminyl transferase 1 | -1.40 | -4.09 | 0.07 | 0.001002 |
| -2.75 | silver | -1.23 | -4.26 | 0.11 | 0.002664 |
| -2.74 | sorbin and SH3 domain containing 2 | -1.20 | -4.27 | 0.12 | 0.002999 |
| -2.74 | sperm associated antigen 11B | -1.52 | -3.94 | 0.05 | 0.000419 |
| -2.72 | sodium channel, voltage-gated, type IX, alpha | -1.32 | -4.09 | 0.08 | 0.001469 |
| -2.67 | Era (G-protein)-like 1 (E. coli) | -1.29 | -4.03 | 0.09 | 0.001538 |
| -2.60 | protein prenyltransferase alpha subunit repeat containing 1 | -1.28 | -3.90 | 0.08 | 0.001297 |
| -2.54 | nitrilase family, member 2 | -1.19 | -3.88 | 0.10 | 0.001924 |
| -2.50 | C1q and tumor necrosis factor related protein 2 | -1.10 | -3.89 | 0.12 | 0.002899 |
| -2.50 | family with sequence similarity 178, member A | -1.01 | -3.97 | 0.14 | 0.004465 |
| -2.48 | olfactory receptor 806 | -1.06 | -3.88 | 0.13 | 0.003441 |
| -2.48 | zinc finger protein 383 | -1.05 | -3.88 | 0.13 | 0.003462 |
| -2.47 | regenerating islet-derived 1 | -1.16 | -3.77 | 0.10 | 0.001914 |
| -2.47 | RIO kinase 1 (yeast) | -1.02 | -3.91 | 0.14 | 0.004077 |
| -2.47 | melanoma inhibitory activity 2 | -1.17 | -3.75 | 0.09 | 0.001770 |
| -2.46 | RIKEN cDNA 4930432K09 gene | -1.12 | -3.77 | 0.11 | 0.002240 |
| -2.43 | ATP-binding cassette, sub-family G (WHITE), member 3 | -1.15 | -3.70 | 0.10 | 0.001797 |
| -2.42 | ubiquitin specific peptidase 36 | -1.02 | -3.81 | 0.13 | 0.003713 |
| -2.40 | RIKEN cDNA 2810442I21 gene | -1.32 | -3.46 | 0.05 | 0.000445 |
| -2.37 | zinc finger and BTB domain containing 46 | -1.05 | -3.68 | 0.12 | 0.002821 |
| -2.36 | methyltransferase like 13 | -1.03 | -3.67 | 0.12 | 0.002979 |
| -2.32 | RIKEN cDNA 2810474C18 gene | -0.93 | -3.68 | 0.14 | 0.004528 |
| -2.30 | RIKEN cDNA C330007P06 gene | -1.00 | -3.58 | 0.12 | 0.003077 |
| -2.29 | olfactory receptor 1170 | -0.99 | -3.57 | 0.12 | 0.003093 |
| -2.27 | potassium intermediate/small conductance calcium-activated channel, subfamily N, member 3 | -0.93 | -3.59 | 0.14 | 0.004093 |
| -2.20 | hypothetical gene supported by AK080816 | -0.87 | -3.52 | 0.15 | 0.005062 |
| -2.19 | zinc fingerprotein 618 | -1.06 | -3.31 | 0.09 | 0.001526 |
| -2.19 | GTP-binding protein 10 (putative); predicted gene 6877 | -1.10 | -3.26 | 0.07 | 0.001068 |
| -2.17 | heat shock protein, alpha-crystallin-related, B9 | -0.99 | -3.35 | 0.11 | 0.002340 |
| -2.16 | olfactory receptor 1424 | -1.03 | -3.27 | 0.09 | 0.001670 |
| -2.10 | predicted gene 2716 | -0.84 | -3.34 | 0.15 | 0.004685 |
| -2.10 | FAD-dependent oxidoreductase domain containing 2 | -1.01 | -3.16 | 0.09 | 0.001499 |
| -2.07 | tripartite motif-containing 15 | -0.82 | -3.30 | 0.15 | 0.004844 |
| -1.97 | tetratricopeptide repeat domain 6 | -0.83 | -3.09 | 0.13 | 0.003642 |
| -1.84 | predicted gene, EG236311 | -0.73 | -2.94 | 0.15 | 0.004946 |
| -1.54 | dyskeratosis congenita 1, dyskerin homolog (human) | -0.64 | -2.43 | 0.13 | 0.003875 |
| -1.53 | FYVE, RhoGEF and PH domain containing 3 | -0.66 | -2.39 | 0.12 | 0.003115 |
| -1.38 | twisted gastrulation homolog 1 (Drosophila) | -0.56 | -2.20 | 0.14 | 0.004264 |

| Age-induced changes in gene expression in the hippocampus  ***Upregulated*** | | **95% confidence intervals** | |  | |
| --- | --- | --- | --- | --- | --- |
| **Percent change** | **Gene name** | **Lower** | **Upper** | **FDR** | **P value** |
| 67.80 | histocompatibility 2, D region; histocompatibility 2, D region locus 1 | 45.71 | 93.25 | 0.00 | 0.000002 |
| 67.47 | complement component 4A | 53.62 | 82.58 | 0.00 | 0.000000 |
| 64.39 | alkaline ceramidase 2 | 49.78 | 80.41 | 0.00 | 0.000000 |
| 63.02 | plasma membrane associated protein, S3-12 | 33.50 | 99.07 | 0.03 | 0.000169 |
| 59.96 | complement component 4B | 47.89 | 73.01 | 0.00 | 0.000000 |
| 58.66 | interleukin 33 | 41.07 | 78.45 | 0.00 | 0.000001 |
| 56.49 | histocompatibility 2, Q region locus 5 | 34.04 | 82.70 | 0.01 | 0.000028 |
| 55.97 | similar to HLA-G protein | 36.79 | 77.85 | 0.00 | 0.000004 |
| 50.85 | predicted gene 7035 | 27.71 | 78.18 | 0.03 | 0.000154 |
| 48.26 | anillin, actin binding protein | 26.53 | 73.73 | 0.03 | 0.000144 |
| 46.89 | lysozyme 1 | 27.66 | 69.02 | 0.02 | 0.000051 |
| 46.61 | similar to apolipoprotein D | 17.99 | 82.17 | 0.12 | 0.003035 |
| 45.79 | beta-2 microglobulin | 24.95 | 70.09 | 0.03 | 0.000170 |
| 44.48 | predicted gene 129 | 25.11 | 66.85 | 0.02 | 0.000107 |
| 41.51 | hypoxia inducible factor 3, alpha subunit | 27.08 | 57.58 | 0.01 | 0.000008 |
| 39.91 | D site albumin promoter binding protein | 23.47 | 58.53 | 0.02 | 0.000063 |
| 39.20 | serum/glucocorticoid regulated kinase 1 | 21.11 | 60.01 | 0.03 | 0.000227 |
| 38.42 | alanine-glyoxylate aminotransferase 2-like 1 | 21.54 | 57.64 | 0.03 | 0.000135 |
| 38.12 | period homolog 2 (Drosophila) | 22.16 | 56.16 | 0.02 | 0.000079 |
| 37.66 | similar to RT1 class I histocompatibility antigen, AA alpha chain precursor | 21.98 | 55.35 | 0.02 | 0.000075 |
| 34.66 | histocompatibility 2, T region locus 23 | 15.96 | 56.36 | 0.07 | 0.001146 |
| 34.18 | predicted gene 8909 | 21.56 | 48.10 | 0.01 | 0.000020 |
| 32.88 | RIKEN cDNA 2310043N10 gene | 19.01 | 48.35 | 0.02 | 0.000097 |
| 32.63 | adiponectin receptor 2 | 22.51 | 43.59 | 0.00 | 0.000002 |
| 32.13 | histocompatibility 2, K1, K region; similar to H-2K(d) antigen | 18.43 | 47.41 | 0.02 | 0.000112 |
| 30.75 | phosphatidylserine decarboxylase, pseudogene 1 | 13.87 | 50.12 | 0.08 | 0.001422 |
| 29.56 | histocompatibility 2, Q region locus 8 | 19.81 | 40.11 | 0.01 | 0.000006 |
| 29.01 | histocompatibility 2, Q region locus 6 | 16.64 | 42.70 | 0.02 | 0.000121 |
| 28.94 | lysozyme 2 | 16.33 | 42.91 | 0.03 | 0.000153 |
| 28.54 | predicted gene 3308 | 17.53 | 40.58 | 0.02 | 0.000040 |
| 28.09 | hyaluronan and proteoglycan link protein 2 | 14.75 | 42.97 | 0.04 | 0.000380 |
| 27.70 | microtubule-associated protein 7 | 16.96 | 39.43 | 0.02 | 0.000043 |
| 27.55 | 5353; immunoglobulin heavy chain (J558 family); Ig H-chain V-JH1-region | 11.90 | 45.39 | 0.10 | 0.002010 |
| 27.20 | solute carrier family 2 (facilitated glucose transporter), member 1 | 20.03 | 34.79 | 0.00 | 0.000000 |
| 26.42 | glial fibrillary acidic protein | 15.10 | 38.85 | 0.03 | 0.000135 |
| 26.18 | heparan sulfate (glucosamine) 3-O-sulfotransferase 3A1 | 16.08 | 37.16 | 0.02 | 0.000042 |
| 25.81 | RIKEN cDNA 6430548M08 gene | 9.84 | 44.10 | 0.14 | 0.004099 |
| 25.47 | period homolog 1 (Drosophila) | 12.10 | 40.44 | 0.07 | 0.001043 |
| 25.38 | sulfotransferase family 1A, phenol-preferring, member 1 | 12.30 | 40.00 | 0.07 | 0.000884 |
| 24.77 | nuclear receptor subfamily 1, group D, member 2; predicted gene 5827 | 13.98 | 36.57 | 0.03 | 0.000168 |
| 24.68 | zinc finger, X-linked, duplicated A | 14.22 | 36.10 | 0.02 | 0.000126 |
| 24.49 | xanthine dehydrogenase | 18.77 | 30.48 | 0.00 | 0.000000 |
| 24.26 | enabled homolog (Drosophila) | 13.16 | 36.44 | 0.04 | 0.000283 |
| 23.52 | solute carrier family 38, member 2 | 12.77 | 35.29 | 0.04 | 0.000285 |
| 23.39 | glutathione S-transferase, theta 3 | 12.44 | 35.40 | 0.04 | 0.000364 |
| 23.38 | Von Willebrand factor homolog | 11.00 | 37.15 | 0.07 | 0.001168 |
| 23.26 | cDNA sequence BC030476 | 12.54 | 34.99 | 0.04 | 0.000311 |
| 22.80 | unc-80 homolog (C. elegans) | 9.82 | 37.31 | 0.11 | 0.002191 |
| 22.68 | similar to Ras and Rab interactor 2; Ras and Rab interactor 2 | 14.84 | 31.05 | 0.01 | 0.000013 |
| 22.40 | AHNAK nucleoprotein (desmoyokin) | 15.75 | 29.44 | 0.00 | 0.000002 |
| 22.09 | proteasome (prosome, macropain) subunit, beta type 8 (large multifunctional peptidase 7) | 11.65 | 33.51 | 0.04 | 0.000413 |
| 22.07 | immunoglobulin kappa chain variable 21 (V21)-1 | 8.62 | 37.18 | 0.13 | 0.003817 |
| 22.01 | Nipped-B homolog (Drosophila) | 8.65 | 37.01 | 0.13 | 0.003696 |
| 21.67 | complement component 3 | 15.29 | 28.40 | 0.00 | 0.000002 |
| 21.09 | predicted gene 14492 | 12.83 | 29.96 | 0.02 | 0.000058 |
| 20.66 | lymphocyte antigen 6 complex, locus C2; lymphocyte antigen 6 complex, locus C1 | 9.12 | 33.42 | 0.10 | 0.001930 |
| 20.57 | protocadherin beta 3 | 13.69 | 27.88 | 0.01 | 0.000009 |
| 20.52 | PDZ and LIM domain 1 (elfin) | 11.52 | 30.25 | 0.03 | 0.000200 |
| 20.35 | ectonucleotide pyrophosphatase/phosphodiesterase 4 | 12.54 | 28.71 | 0.02 | 0.000047 |
| 19.73 | peptidoglycan recognition protein 1; similar to peptidoglycan recognition protein | 11.92 | 28.08 | 0.02 | 0.000068 |
| 19.40 | ras homolog gene family, member U | 11.16 | 28.24 | 0.03 | 0.000146 |
| 19.24 | interferon-induced protein with tetratricopeptide repeats 3 | 11.70 | 27.29 | 0.02 | 0.000062 |
| 19.16 | thioredoxin interacting protein | 7.99 | 31.48 | 0.12 | 0.002793 |
| 18.88 | predicted gene 7378; rhomboid, veinlet-like 2 (Drosophila) | 9.30 | 29.30 | 0.07 | 0.000858 |
| 18.83 | dopa decarboxylase | 8.95 | 29.61 | 0.07 | 0.001162 |
| 18.76 | absent in melanoma 1-like | 8.32 | 30.20 | 0.10 | 0.001925 |
| 18.73 | folliculin interacting protein 1 | 12.23 | 25.61 | 0.01 | 0.000015 |
| 18.58 | cytochrome P450, family 27, subfamily a, polypeptide 1 | 7.74 | 30.50 | 0.12 | 0.002830 |
| 18.40 | Nipped-B homolog (Drosophila) | 9.02 | 28.58 | 0.07 | 0.000906 |
| 18.37 | interferon regulatory factor 1 | 9.29 | 28.20 | 0.06 | 0.000682 |
| 18.21 | perforin 1 (pore forming protein) | 7.83 | 29.59 | 0.11 | 0.002371 |
| 18.14 | hypothetical protein LOC633360 | 9.74 | 27.17 | 0.04 | 0.000366 |
| 17.91 | dystrobrevin, beta | 9.33 | 27.16 | 0.05 | 0.000513 |
| 17.90 | CDK5 and Abl enzyme substrate 1 | 7.36 | 29.47 | 0.12 | 0.003078 |
| 17.89 | lysozyme 2 | 9.79 | 26.60 | 0.04 | 0.000297 |
| 17.78 | dedicator of cytokinesis 4 | 8.33 | 28.05 | 0.08 | 0.001323 |
| 17.67 | transglutaminase 2, C polypeptide | 9.56 | 26.38 | 0.04 | 0.000339 |
| 17.59 | glutathione S-transferase, mu 1 | 9.54 | 26.22 | 0.04 | 0.000328 |
| 17.50 | zinc finger, CCHC domain containing 24 | 11.01 | 24.38 | 0.01 | 0.000035 |
| 17.50 | endonuclease domain containing 1 | 10.24 | 25.23 | 0.02 | 0.000120 |
| 17.40 | CDC-like kinase 1 | 8.48 | 27.07 | 0.07 | 0.000977 |
| 17.36 | complement component 1, q subcomponent, C chain | 8.75 | 26.65 | 0.06 | 0.000718 |
| 17.36 | sarcospan | 8.21 | 27.28 | 0.08 | 0.001242 |
| 17.32 | transcription factor CP2-like 1 | 11.69 | 23.24 | 0.01 | 0.000007 |
| 17.28 | transmembrane protein 200B | 7.74 | 27.67 | 0.10 | 0.001846 |
| 17.24 | TSC22 domain family, member 3 | 10.18 | 24.76 | 0.02 | 0.000106 |
| 17.22 | Kruppel-like factor 9 | 7.14 | 28.25 | 0.12 | 0.002967 |
| 17.21 | aldehyde dehydrogenase family 6, subfamily A1 | 7.00 | 28.40 | 0.13 | 0.003299 |
| 17.21 | homocysteine-inducible, endoplasmic reticulum stress-inducible, ubiquitin-like domain member 1 | 7.51 | 27.78 | 0.11 | 0.002186 |
| 17.20 | dual specificity phosphatase 5 | 9.79 | 25.11 | 0.03 | 0.000180 |
| 17.20 | basic helix-loop-helix family, member e40 | 6.53 | 28.94 | 0.15 | 0.004632 |
| 17.13 | phosphatase and actin regulator 4 | 7.26 | 27.91 | 0.11 | 0.002619 |
| 17.13 | RIKEN cDNA 6430706D22 gene | 9.68 | 25.08 | 0.03 | 0.000199 |
| 17.04 | unc-84 homolog B (C. elegans) | 7.89 | 26.97 | 0.08 | 0.001470 |
| 16.85 | calcium channel, voltage-dependent, beta 2 subunit | 6.74 | 27.92 | 0.13 | 0.003625 |
| 16.79 | ribonuclease, RNase A family 4 | 10.43 | 23.52 | 0.02 | 0.000045 |
| 16.62 | predicted gene 4671 | 7.48 | 26.53 | 0.10 | 0.001806 |
| 16.58 | carboxypeptidase M | 10.53 | 22.96 | 0.01 | 0.000029 |
| 16.56 | ATP-binding cassette, sub-family A (ABC1), member 8a | 7.47 | 26.42 | 0.10 | 0.001782 |
| 16.53 | TSC22 domain family, member 2 | 7.90 | 25.84 | 0.07 | 0.001156 |
| 16.41 | serine racemase | 6.69 | 27.02 | 0.13 | 0.003298 |
| 16.29 | cyclin T2 | 8.25 | 24.92 | 0.06 | 0.000699 |
| 16.28 | neurexin III | 10.28 | 22.59 | 0.01 | 0.000033 |
| 16.19 | S100 calcium binding protein A8 (calgranulin A) | 7.81 | 25.22 | 0.07 | 0.001081 |
| 15.71 | histocompatibility 2, class II antigen A, beta 1; response to metastatic cancers 2 | 6.47 | 25.76 | 0.12 | 0.003156 |
| 15.50 | GRIP1 associated protein 1 | 6.48 | 25.29 | 0.12 | 0.002903 |
| 15.42 | histocompatibility 2, T region locus 9; histocompatibility 2, T region locus 10; histocompatibility 2, T region locus 22 | 10.69 | 20.36 | 0.00 | 0.000004 |
| 15.20 | chromodomain helicase DNA binding protein 7 | 7.87 | 23.04 | 0.05 | 0.000580 |
| 15.08 | SMEK homolog 2, suppressor of mek1 (Dictyostelium) | 8.64 | 21.90 | 0.03 | 0.000174 |
| 15.07 | guanine nucleotide binding protein, alpha 13 | 8.00 | 22.60 | 0.05 | 0.000446 |
| 15.04 | low density lipoprotein-related protein 1B (deleted in tumors) | 6.80 | 23.92 | 0.10 | 0.001793 |
| 15.00 | similar to Stat3B; signal transducer and activator of transcription 3 | 9.45 | 20.82 | 0.01 | 0.000036 |
| 14.88 | ST6 (alpha-N-acetyl-neuraminyl-2,3-beta-galactosyl-1,3)-N-acetylgalactosaminide alpha-2,6-sialyltransferase 3 | 7.39 | 22.88 | 0.07 | 0.000862 |
| 14.83 | protocadherin beta 7 | 9.86 | 20.02 | 0.01 | 0.000011 |
| 14.79 | interferon regulatory factor 2 | 6.05 | 24.25 | 0.13 | 0.003303 |
| 14.73 | diacylglycerol kinase, gamma | 5.84 | 24.37 | 0.14 | 0.003884 |
| 14.73 | cytochrome P450, family 2, subfamily d, polypeptide 22 | 7.77 | 22.13 | 0.05 | 0.000480 |
| 14.65 | microtubule associated serine/threonine kinase family member 4 | 10.23 | 19.24 | 0.00 | 0.000003 |
| 14.63 | formyl peptide receptor 2 | 7.11 | 22.69 | 0.07 | 0.001050 |
| 14.32 | transmembrane and coiled coil domains 1 | 6.62 | 22.57 | 0.09 | 0.001554 |
| 14.23 | PH domain and leucine rich repeat protein phosphatase 1 | 9.12 | 19.59 | 0.01 | 0.000026 |
| 14.23 | zinc finger protein 445 | 6.36 | 22.67 | 0.10 | 0.001959 |
| 14.20 | nucleolar protein 3 (apoptosis repressor with CARD domain) | 10.19 | 18.36 | 0.00 | 0.000001 |
| 14.20 | glutamate receptor, ionotropic, AMPA2 (alpha 2) | 6.44 | 22.53 | 0.10 | 0.001786 |
| 14.17 | ring finger protein 213 | 7.82 | 20.90 | 0.04 | 0.000291 |
| 14.17 | c-mer proto-oncogene tyrosine kinase | 7.40 | 21.37 | 0.05 | 0.000544 |
| 13.93 | insulin-like growth factor I receptor | 7.68 | 20.55 | 0.04 | 0.000296 |
| 13.92 | CDC like kinase 4 | 8.35 | 19.79 | 0.02 | 0.000091 |
| 13.80 | coiled-coil domain containing 117 | 6.84 | 21.20 | 0.07 | 0.000892 |
| 13.71 | adaptor protein, phosphotyrosine interaction, PH domain and leucine zipper containing 2 | 7.95 | 19.78 | 0.03 | 0.000151 |
| 13.66 | family with sequence similarity 126, member B | 8.38 | 19.19 | 0.02 | 0.000062 |
| 13.63 | choline kinase alpha | 6.27 | 21.51 | 0.09 | 0.001633 |
| 13.62 | Niemann Pick type C1 | 6.43 | 21.30 | 0.08 | 0.001343 |
| 13.61 | myeloid/lymphoid or mixed-lineage leukemia 3 | 8.19 | 19.30 | 0.02 | 0.000085 |
| 13.60 | polymerase (RNA) III (DNA directed) polypeptide E | 10.33 | 16.97 | 0.00 | 0.000000 |
| 13.58 | SET domain containing 1B | 5.42 | 22.38 | 0.13 | 0.003826 |
| 13.55 | serine (or cysteine) preptidase inhibitor, clade A, member 1B | 6.81 | 20.71 | 0.06 | 0.000799 |
| 13.53 | nischarin | 6.66 | 20.84 | 0.07 | 0.000953 |
| 13.50 | RIKEN cDNA 2900027M19 gene | 6.48 | 20.99 | 0.08 | 0.001188 |
| 13.42 | lin-54 homolog (C. elegans) | 6.77 | 20.48 | 0.06 | 0.000769 |
| 13.39 | PTC7 protein phosphatase homolog (S. cerevisiae) | 5.38 | 22.01 | 0.13 | 0.003709 |
| 13.23 | unc-84 homolog B (C. elegans) | 5.70 | 21.30 | 0.11 | 0.002516 |
| 13.03 | EGF-like module containing, mucin-like, hormone receptor-like sequence 1 | 8.85 | 17.37 | 0.01 | 0.000007 |
| 13.03 | von Willebrand factor A domain containing 5A | 7.89 | 18.41 | 0.02 | 0.000078 |
| 12.92 | synemin, intermediate filament protein | 5.25 | 21.16 | 0.13 | 0.003528 |
| 12.90 | membrane bound O-acyltransferase domain containing 1 | 5.52 | 20.79 | 0.11 | 0.002635 |
| 12.81 | nuclear factor of kappa light polypeptide gene enhancer in B-cells inhibitor, alpha | 6.00 | 20.05 | 0.08 | 0.001444 |
| 12.80 | Rap guanine nucleotide exchange factor (GEF) 4 | 5.54 | 20.57 | 0.11 | 0.002484 |
| 12.67 | proprotein convertase subtilisin/kexin type 2 | 5.49 | 20.33 | 0.11 | 0.002450 |
| 12.39 | spastic paraplegia 20, spartin (Troyer syndrome) homolog (human) | 5.62 | 19.60 | 0.10 | 0.001835 |
| 12.39 | thyroid hormone receptor associated protein 3; predicted gene 5898 | 7.35 | 17.66 | 0.02 | 0.000111 |
| 12.30 | similar to mKIAA1021 protein | 5.70 | 19.32 | 0.09 | 0.001592 |
| 12.27 | transformed mouse 3T3 cell double minute 2 | 7.16 | 17.62 | 0.03 | 0.000144 |
| 12.22 | leucine zipper, putative tumor suppressor 2 | 5.70 | 19.15 | 0.09 | 0.001517 |
| 12.19 | oxysterol binding protein-like 6 | 6.64 | 18.03 | 0.04 | 0.000356 |
| 12.07 | sema domain, immunoglobulin domain, transmembrane and short cytoplasmic domain, 4B | 4.98 | 19.64 | 0.13 | 0.003282 |
| 11.96 | potassium voltage-gated channel, subfamily Q, member 2 | 6.11 | 18.14 | 0.06 | 0.000709 |
| 11.92 | kinesin family member 1B | 7.68 | 16.32 | 0.01 | 0.000025 |
| 11.90 | hedgehog acyltransferase-like | 4.55 | 19.76 | 0.15 | 0.004783 |
| 11.90 | expressed sequence AI314180 | 6.23 | 17.86 | 0.05 | 0.000550 |
| 11.87 | CCR4-NOT transcription complex, subunit 4 | 4.68 | 19.55 | 0.14 | 0.004127 |
| 11.84 | mannose-6-phosphate receptor, cation dependent | 5.16 | 18.95 | 0.11 | 0.002399 |
| 11.83 | RIKEN cDNA 4930546H06 gene | 5.95 | 18.02 | 0.06 | 0.000812 |
| 11.78 | 5-azacytidine induced gene 2 | 6.53 | 17.28 | 0.04 | 0.000290 |
| 11.77 | kelch repeat and BTB (POZ) domain containing 5 | 6.53 | 17.27 | 0.04 | 0.000289 |
| 11.68 | predicted gene 5881 | 4.88 | 18.92 | 0.12 | 0.003089 |
| 11.64 | gametogenetin binding protein 1; RIKEN cDNA 0610031G08 gene | 7.12 | 16.34 | 0.02 | 0.000069 |
| 11.62 | GRB10 interacting GYF protein 1 | 5.73 | 17.83 | 0.07 | 0.000972 |
| 11.60 | cytoplasmic polyadenylation element binding protein 1 | 4.78 | 18.88 | 0.13 | 0.003341 |
| 11.58 | ribosomal protein S6 kinase polypeptide 1 | 4.97 | 18.61 | 0.11 | 0.002644 |
| 11.54 | nuclear factor of kappa light polypeptide gene enhancer in B-cells inhibitor, zeta | 5.74 | 17.66 | 0.07 | 0.000908 |
| 11.52 | peripheral myelin protein 22 | 6.93 | 16.31 | 0.02 | 0.000092 |
| 11.52 | sorting nexin family member 30 | 4.57 | 18.93 | 0.14 | 0.004025 |
| 11.45 | dihydrodiol dehydrogenase (dimeric) | 6.80 | 16.30 | 0.02 | 0.000112 |
| 11.39 | lamin A | 5.43 | 17.68 | 0.08 | 0.001300 |
| 11.37 | BTB and CNC homology 1 | 5.04 | 18.07 | 0.10 | 0.002174 |
| 11.34 | enhancer of polycomb homolog 2 (Drosophila); similar to Enhancer of polycomb homolog 2 | 4.80 | 18.29 | 0.12 | 0.002870 |
| 11.25 | RIKEN cDNA 1500012F01 gene | 5.60 | 17.20 | 0.07 | 0.000903 |
| 11.22 | serine/threonine kinase 32C | 4.32 | 18.58 | 0.15 | 0.004685 |
| 11.21 | rho/rac guanine nucleotide exchange factor (GEF) 2 | 6.14 | 16.52 | 0.04 | 0.000340 |
| 11.21 | rhophilin, Rho GTPase binding protein 2 | 7.61 | 14.92 | 0.01 | 0.000007 |
| 11.16 | plectin 1 | 5.41 | 17.23 | 0.07 | 0.001145 |
| 11.11 | transforming, acidic coiled-coil containing protein 1 | 5.02 | 17.56 | 0.10 | 0.001932 |
| 11.10 | solute carrier family 6 (neurotransmitter transporter, taurine), member 6 | 6.76 | 15.61 | 0.02 | 0.000076 |
| 11.06 | pre B-cell leukemia transcription factor 1 | 5.97 | 16.40 | 0.04 | 0.000405 |
| 11.05 | Fas (TNF receptor superfamily member 6) | 7.51 | 14.72 | 0.01 | 0.000007 |
| 11.03 | sin3 associated polypeptide | 5.89 | 16.41 | 0.05 | 0.000459 |
| 10.96 | Fc receptor, IgG, low affinity III | 5.88 | 16.28 | 0.05 | 0.000439 |
| 10.94 | predicted gene 7527; family with sequence similarity 76, member A | 4.32 | 17.97 | 0.14 | 0.004165 |
| 10.92 | cytochrome b-245, alpha polypeptide | 7.35 | 14.61 | 0.01 | 0.000009 |
| 10.91 | transcription factor Dp 2 | 7.21 | 14.73 | 0.01 | 0.000015 |
| 10.81 | death effector domain-containing DNA binding protein 2 | 5.31 | 16.60 | 0.07 | 0.001029 |
| 10.78 | doublecortin-like kinase 1 | 4.46 | 17.48 | 0.13 | 0.003285 |
| 10.77 | prolyl endopeptidase-like | 4.56 | 17.36 | 0.12 | 0.002898 |
| 10.71 | metallothionein 1 | 4.39 | 17.40 | 0.13 | 0.003441 |
| 10.68 | jumonji domain containing 6 | 5.90 | 15.66 | 0.04 | 0.000309 |
| 10.65 | PR domain containing 2, with ZNF domain | 6.87 | 14.57 | 0.01 | 0.000026 |
| 10.64 | similar to polycomb group ring finger 5; polycomb group ring finger 5 | 4.62 | 16.99 | 0.11 | 0.002486 |
| 10.62 | SWI/SNF related, matrix associated, actin dependent regulator of chromatin, subfamily d, member 2 | 4.56 | 17.03 | 0.11 | 0.002666 |
| 10.62 | UDP glycosyltransferase 1 family polypeptide A10 | 6.10 | 15.33 | 0.03 | 0.000188 |
| 10.62 | S100 calcium binding protein A9 (calgranulin B) | 6.79 | 14.59 | 0.01 | 0.000031 |
| 10.60 | Kruppel-like factor 9 | 5.30 | 16.18 | 0.07 | 0.000893 |
| 10.59 | mesoderm induction early response 1, family member 3; similar to Mier3 protein | 4.10 | 17.49 | 0.15 | 0.004602 |
| 10.59 | Kruppel-like factor 2 (lung) | 5.12 | 16.34 | 0.08 | 0.001179 |
| 10.55 | filamin, beta | 4.23 | 17.25 | 0.13 | 0.003869 |
| 10.55 | kelch-like 21 (Drosophila) | 5.12 | 16.25 | 0.07 | 0.001141 |
| 10.52 | golgi apparatus protein 1 | 4.68 | 16.68 | 0.10 | 0.002141 |
| 10.51 | zinc finger protein 263 | 5.84 | 15.39 | 0.04 | 0.000294 |
| 10.46 | cytoplasmic polyadenylation element binding protein 3 | 4.12 | 17.18 | 0.14 | 0.004239 |
| 10.42 | nanos homolog 2 (Drosophila) | 7.23 | 13.71 | 0.00 | 0.000004 |
| 10.41 | ERBB receptor feedback inhibitor 1 | 4.63 | 16.51 | 0.10 | 0.002160 |
| 10.41 | ArfGAP with GTPase domain, ankyrin repeat and PH domain 1 | 4.51 | 16.64 | 0.11 | 0.002540 |
| 10.38 | DNA segment, Chr 10, Brigham & Women's Genetics 1379 expressed | 4.10 | 17.05 | 0.14 | 0.004208 |
| 10.32 | WNK lysine deficient protein kinase 2 | 4.68 | 16.26 | 0.10 | 0.001893 |
| 10.29 | SAPS domain family, member 3 | 6.15 | 14.59 | 0.02 | 0.000105 |
| 10.29 | Rap guanine nucleotide exchange factor (GEF) 4 | 5.31 | 15.50 | 0.06 | 0.000666 |
| 10.27 | serine/threonine/tyrosine interaction protein; predicted gene 14698 | 4.01 | 16.90 | 0.14 | 0.004402 |
| 10.19 | patatin-like phospholipase domain containing 2 | 4.16 | 16.57 | 0.13 | 0.003578 |
| 10.17 | tripartite motif-containing 35 | 6.01 | 14.49 | 0.02 | 0.000126 |
| 10.17 | period homolog 3 (Drosophila) | 5.61 | 14.92 | 0.04 | 0.000320 |
| 10.10 | transforming, acidic coiled-coil containing protein 1 | 5.28 | 15.14 | 0.05 | 0.000588 |
| 10.05 | protein kinase D3 | 3.93 | 16.54 | 0.14 | 0.004438 |
| 10.02 | family with sequence similarity 131, member B | 3.80 | 16.62 | 0.15 | 0.005072 |
| 10.02 | KRIT1, ankyrin repeat containing | 4.01 | 16.37 | 0.14 | 0.003929 |
| 10.00 | sorting nexin 1 | 3.94 | 16.40 | 0.14 | 0.004253 |
| 9.96 | peptidyl arginine deiminase, type II; similar to peptidyl arginine deiminase, type II | 5.04 | 15.10 | 0.06 | 0.000797 |
| 9.92 | potassium inwardly-rectifying channel, subfamily J, member 6 | 6.30 | 13.66 | 0.01 | 0.000036 |
| 9.91 | zinc finger, MYM-type 5 | 6.71 | 13.20 | 0.01 | 0.000008 |
| 9.86 | MAM domain containing glycosylphosphatidylinositol anchor 2 | 5.43 | 14.48 | 0.04 | 0.000332 |
| 9.86 | procollagen C-endopeptidase enhancer 2 | 3.91 | 16.14 | 0.14 | 0.004120 |
| 9.85 | solute carrier family 24 (sodium/potassium/calcium exchanger), member 2 | 4.69 | 15.28 | 0.08 | 0.001364 |
| 9.83 | RIKEN cDNA B230340J04 gene | 3.79 | 16.21 | 0.15 | 0.004693 |
| 9.82 | CCR4-NOT transcription complex, subunit 6 | 4.40 | 15.51 | 0.10 | 0.002073 |
| 9.76 | heat-responsive protein 12 | 4.01 | 15.82 | 0.13 | 0.003460 |
| 9.76 | zinc finger homeobox 2; similar to Zinc finger protein 409 | 4.22 | 15.59 | 0.11 | 0.002615 |
| 9.74 | proteasome (prosome, macropain) activator subunit 4 | 5.67 | 13.95 | 0.03 | 0.000157 |
| 9.72 | adenylate cyclase 9 | 4.36 | 15.34 | 0.10 | 0.002051 |
| 9.71 | A kinase (PRKA) anchor protein 8-like | 5.34 | 14.26 | 0.04 | 0.000335 |
| 9.56 | WD repeat and FYVE domain containing 3 | 5.14 | 14.16 | 0.05 | 0.000437 |
| 9.56 | MYST histone acetyltransferase monocytic leukemia 4 | 4.03 | 15.38 | 0.12 | 0.003033 |
| 9.55 | glypican 5 | 4.82 | 14.49 | 0.06 | 0.000830 |
| 9.51 | diacylglycerol lipase, beta | 3.87 | 15.45 | 0.13 | 0.003633 |
| 9.47 | NHL repeat containing 2 | 3.60 | 15.68 | 0.15 | 0.005077 |
| 9.46 | dual specificity phosphatase 11 (RNA/RNP complex 1-interacting) | 3.83 | 15.40 | 0.13 | 0.003740 |
| 9.42 | LIM domain binding 1 | 4.18 | 14.92 | 0.11 | 0.002223 |
| 9.40 | pyruvate dehydrogenase kinase, isoenzyme 4 | 4.44 | 14.61 | 0.08 | 0.001460 |
| 9.40 | polymerase (DNA directed), kappa | 4.21 | 14.85 | 0.10 | 0.002110 |
| 9.38 | neural cell adhesion molecule 1 | 4.42 | 14.57 | 0.08 | 0.001464 |
| 9.37 | nitric oxide synthase trafficker | 3.96 | 15.06 | 0.12 | 0.003001 |
| 9.33 | 2'-5' oligoadenylate synthetase-like 2 | 4.42 | 14.47 | 0.08 | 0.001422 |
| 9.32 | anterior pharynx defective 1b homolog (C. elegans) | 3.79 | 15.14 | 0.13 | 0.003655 |
| 9.28 | protocadherin beta 19 | 6.06 | 12.60 | 0.01 | 0.000021 |
| 9.26 | ATPase, class V, type 10A | 6.24 | 12.37 | 0.01 | 0.000010 |
| 9.25 | family with sequence similarity 116, member A | 4.76 | 13.94 | 0.06 | 0.000704 |
| 9.21 | kinase suppressor of ras 1 | 4.53 | 14.10 | 0.07 | 0.001047 |
| 9.21 | interferon gamma receptor 2 | 3.74 | 14.96 | 0.13 | 0.003696 |
| 9.20 | glioma tumor suppressor candidate region gene 2 | 4.91 | 13.68 | 0.05 | 0.000485 |
| 9.15 | TBC1 domain family, member 22a | 3.89 | 14.67 | 0.12 | 0.002889 |
| 9.13 | nucleotide-binding oligomerization domain containing 1 | 4.90 | 13.53 | 0.05 | 0.000449 |
| 9.11 | phosphodiesterase 4B, cAMP specific | 3.69 | 14.82 | 0.13 | 0.003782 |
| 9.09 | BAT2 domain containing 1; predicted gene 4972 | 3.52 | 14.96 | 0.15 | 0.004661 |
| 9.01 | embryonic ectoderm development | 3.54 | 14.77 | 0.14 | 0.004374 |
| 8.98 | family with sequence similarity 178, member A | 4.48 | 13.68 | 0.07 | 0.000935 |
| 8.93 | cysteine-serine-rich nuclear protein 1 | 3.85 | 14.26 | 0.11 | 0.002667 |
| 8.91 | zinc finger, FYVE domain containing 21 | 4.34 | 13.68 | 0.07 | 0.001141 |
| 8.90 | cell division cycle 37 homolog (S. cerevisiae)-like 1 | 3.97 | 14.07 | 0.11 | 0.002188 |
| 8.88 | La ribonucleoprotein domain family, member 4; predicted gene 14373; predicted gene 8177 | 5.28 | 12.62 | 0.02 | 0.000121 |
| 8.88 | similar to Anxa3; annexin A3 | 3.50 | 14.54 | 0.14 | 0.004327 |
| 8.86 | splicing factor, arginine/serine-rich 11 | 4.56 | 13.34 | 0.06 | 0.000708 |
| 8.85 | similar to myeloid cell leukemia sequence 1; myeloid cell leukemia sequence 1 | 4.14 | 13.78 | 0.09 | 0.001583 |
| 8.82 | TAF9B RNA polymerase II, TATA box binding protein (TBP)-associated factor | 4.09 | 13.76 | 0.09 | 0.001669 |
| 8.81 | mannose-6-phosphate receptor binding protein 1 | 4.43 | 13.37 | 0.07 | 0.000876 |
| 8.79 | polyhomeotic-like 3 (Drosophila) | 5.32 | 12.37 | 0.02 | 0.000090 |
| 8.75 | synaptotagmin binding, cytoplasmic RNA interacting protein | 3.65 | 14.10 | 0.13 | 0.003260 |
| 8.74 | eukaryotic translation initiation factor 2a | 4.63 | 13.02 | 0.05 | 0.000526 |
| 8.74 | neuron-glia-CAM-related cell adhesion molecule | 3.41 | 14.34 | 0.14 | 0.004543 |
| 8.73 | P450 (cytochrome) oxidoreductase | 4.49 | 13.14 | 0.06 | 0.000709 |
| 8.72 | folate hydrolase | 4.02 | 13.63 | 0.09 | 0.001739 |
| 8.72 | CD52 antigen | 4.39 | 13.22 | 0.07 | 0.000856 |
| 8.70 | folliculin | 4.42 | 13.16 | 0.06 | 0.000797 |
| 8.67 | peptidyl arginine deiminase, type II | 4.03 | 13.51 | 0.09 | 0.001649 |
| 8.66 | sortilin 1 | 3.49 | 14.10 | 0.14 | 0.003920 |
| 8.66 | similar to zinc finger and BTB domain containing 40; zinc finger and BTB domain containing 40 | 4.71 | 12.76 | 0.04 | 0.000397 |
| 8.63 | Rho guanine nucleotide exchange factor (GEF) 1 | 6.05 | 11.28 | 0.00 | 0.000003 |
| 8.58 | UPF1 regulator of nonsense transcripts homolog (yeast) | 4.26 | 13.09 | 0.07 | 0.000991 |
| 8.57 | oncostatin M receptor | 5.69 | 11.53 | 0.01 | 0.000015 |
| 8.53 | SFT2 domain containing 2 | 5.00 | 12.19 | 0.03 | 0.000151 |
| 8.51 | Ral GEF with PH domain and SH3 binding motif 1 | 3.96 | 13.26 | 0.09 | 0.001636 |
| 8.51 | DDRGK domain containing 1 | 4.14 | 13.05 | 0.07 | 0.001151 |
| 8.50 | CDC14 cell division cycle 14 homolog B (S. cerevisiae) | 4.52 | 12.63 | 0.05 | 0.000503 |
| 8.50 | pleckstrin homology domain-containing, family A (phosphoinositide binding specific) member 3 | 3.39 | 13.86 | 0.14 | 0.004111 |
| 8.48 | elongation factor RNA polymerase II | 5.35 | 11.71 | 0.02 | 0.000044 |
| 8.47 | phosphatase and tensin homolog | 3.67 | 13.49 | 0.11 | 0.002613 |
| 8.44 | RIKEN cDNA 4833426J09 gene | 3.56 | 13.55 | 0.12 | 0.003058 |
| 8.41 | ankyrin repeat and KH domain containing 1; eukaryotic translation initiation factor 4E binding protein 3 | 5.23 | 11.68 | 0.02 | 0.000056 |
| 8.40 | protein tyrosine phosphatase-like (proline instead of catalytic arginine), member b | 4.70 | 12.24 | 0.04 | 0.000287 |
| 8.40 | predicted gene 3555; predicted gene 7451; serine/threonine kinase 38 like | 4.45 | 12.50 | 0.05 | 0.000531 |
| 8.38 | pleckstrin homology domain containing, family M, member 3 | 3.53 | 13.46 | 0.12 | 0.003104 |
| 8.38 | itchy, E3 ubiquitin protein ligase | 3.53 | 13.46 | 0.12 | 0.003106 |
| 8.37 | sialic acid binding Ig-like lectin H | 3.74 | 13.20 | 0.10 | 0.002160 |
| 8.29 | pleckstrin homology domain containing, family F (with FYVE domain) member 1 | 3.43 | 13.37 | 0.13 | 0.003391 |
| 8.29 | cytochrome P450, family 4, subfamily f, polypeptide 13 | 3.41 | 13.39 | 0.13 | 0.003518 |
| 8.27 | protocadherin beta 14 | 5.90 | 10.69 | 0.00 | 0.000002 |
| 8.24 | ATP-binding cassette, sub-family G (WHITE), member 1 | 4.61 | 12.00 | 0.04 | 0.000286 |
| 8.23 | glutathione S-transferase, alpha 3 | 4.65 | 11.93 | 0.04 | 0.000250 |
| 8.23 | HECT, C2 and WW domain containing E3 ubiquitin protein ligase 1 | 3.44 | 13.24 | 0.13 | 0.003235 |
| 8.21 | Bardet-Biedl syndrome 5 (human) | 4.16 | 12.41 | 0.06 | 0.000813 |
| 8.20 | NMDA receptor regulated 1-like | 4.14 | 12.41 | 0.06 | 0.000845 |
| 8.20 | gametogenetin binding protein 2 | 4.65 | 11.86 | 0.03 | 0.000235 |
| 8.14 | CLIP associating protein 1 | 4.37 | 12.06 | 0.05 | 0.000471 |
| 8.14 | oviductal glycoprotein 1 | 4.14 | 12.30 | 0.06 | 0.000803 |
| 8.14 | zinc finger protein 420 | 3.38 | 13.11 | 0.13 | 0.003337 |
| 8.12 | acidic (leucine-rich) nuclear phosphoprotein 32 family, member E | 3.56 | 12.87 | 0.11 | 0.002449 |
| 8.12 | phosphatidylinositol 4-kinase, catalytic, alpha polypeptide | 3.13 | 13.34 | 0.15 | 0.004805 |
| 8.10 | matrix-remodelling associated 7 | 3.23 | 13.20 | 0.14 | 0.004131 |
| 8.10 | cysteinyl-tRNA synthetase 2 (mitochondrial)(putative) | 4.51 | 11.80 | 0.04 | 0.000298 |
| 8.09 | Rap guanine nucleotide exchange factor (GEF) 3 | 4.52 | 11.79 | 0.04 | 0.000292 |
| 8.06 | tyrosine kinase, non-receptor, 2 | 3.87 | 12.41 | 0.08 | 0.001296 |
| 8.04 | kinesin family member 17 | 5.59 | 10.55 | 0.00 | 0.000005 |
| 8.03 | UDP-GalNAc:betaGlcNAc beta 1,3-galactosaminyltransferase, polypeptide 2 | 3.39 | 12.87 | 0.12 | 0.003058 |
| 8.03 | filamin C, gamma | 4.04 | 12.17 | 0.07 | 0.000887 |
| 8.01 | trinucleotide repeat containing 6C | 3.44 | 12.79 | 0.12 | 0.002782 |
| 8.01 | Fc receptor, IgG, low affinity IIb | 4.77 | 11.34 | 0.02 | 0.000120 |
| 7.97 | centrosomal protein 120 | 4.16 | 11.92 | 0.05 | 0.000620 |
| 7.96 | RIKEN cDNA I830012O16 gene | 4.94 | 11.06 | 0.02 | 0.000060 |
| 7.96 | Sec24 related gene family, member B (S. cerevisiae) | 4.80 | 11.21 | 0.02 | 0.000098 |
| 7.95 | RIKEN cDNA B230206H07 gene | 3.29 | 12.82 | 0.13 | 0.003421 |
| 7.92 | plakophilin 4 | 3.81 | 12.19 | 0.08 | 0.001294 |
| 7.85 | ELKS/RAB6-interacting/CAST family member 2 | 3.79 | 12.07 | 0.08 | 0.001257 |
| 7.81 | WAS/WASL interacting protein family, member 2 | 3.00 | 12.84 | 0.15 | 0.004910 |
| 7.76 | ubiquitin specific peptidase 1; predicted gene 5841 | 3.10 | 12.63 | 0.14 | 0.004127 |
| 7.75 | RUN and FYVE domain containing 3 | 3.29 | 12.40 | 0.12 | 0.002982 |
| 7.72 | solute carrier family 27 (fatty acid transporter), member 2 | 3.08 | 12.57 | 0.14 | 0.004115 |
| 7.70 | regulatory solute carrier protein, family 1, member 1; DNA-damage inducible protein 2 | 3.36 | 12.23 | 0.11 | 0.002550 |
| 7.66 | RAB5B, member RAS oncogene family | 3.51 | 11.98 | 0.10 | 0.001854 |
| 7.64 | mannosidase 2, alpha B1 | 4.33 | 11.06 | 0.04 | 0.000248 |
| 7.64 | integrator complex subunit 6 | 2.99 | 12.49 | 0.14 | 0.004549 |
| 7.62 | deltex 2 homolog (Drosophila) | 3.27 | 12.15 | 0.12 | 0.002799 |
| 7.61 | dynamin 1 | 4.14 | 11.21 | 0.04 | 0.000410 |
| 7.59 | sterile alpha motif domain containing 9-like | 3.15 | 12.23 | 0.13 | 0.003389 |
| 7.58 | expressed sequence AI314180 | 3.12 | 12.24 | 0.13 | 0.003562 |
| 7.55 | RAD50 homolog (S. cerevisiae) | 4.65 | 10.53 | 0.02 | 0.000070 |
| 7.54 | similar to protocadherin alpha 8 | 4.63 | 10.53 | 0.02 | 0.000073 |
| 7.53 | selenium binding protein 1; hypothetical protein LOC100044204 | 3.04 | 12.22 | 0.14 | 0.003896 |
| 7.52 | integrin beta 5 | 2.91 | 12.34 | 0.15 | 0.004823 |
| 7.51 | dynactin 4 | 3.21 | 11.99 | 0.12 | 0.002862 |
| 7.50 | elastin microfibril interfacer 2 | 4.44 | 10.64 | 0.03 | 0.000132 |
| 7.49 | choline kinase beta | 4.36 | 10.72 | 0.03 | 0.000172 |
| 7.45 | protocadherin beta 2 | 5.05 | 9.91 | 0.01 | 0.000009 |
| 7.44 | tuberous sclerosis 1 | 3.28 | 11.77 | 0.11 | 0.002384 |
| 7.44 | zinc finger protein 574 | 3.11 | 11.94 | 0.13 | 0.003250 |
| 7.43 | sirtuin 7 (silent mating type information regulation 2, homolog) 7 (S. cerevisiae) | 3.51 | 11.49 | 0.09 | 0.001502 |
| 7.39 | retinoic acid induced 1 | 3.50 | 11.44 | 0.08 | 0.001474 |
| 7.34 | ATPase, aminophospholipid transporter (APLT), class I, type 8A, member 1 | 3.90 | 10.91 | 0.05 | 0.000536 |
| 7.29 | hydroxysteroid (17-beta) dehydrogenase 11 | 3.03 | 11.73 | 0.13 | 0.003371 |
| 7.24 | predicted gene 4589 | 4.40 | 10.16 | 0.02 | 0.000088 |
| 7.24 | DPH2 homolog (S. cerevisiae) | 3.02 | 11.64 | 0.13 | 0.003343 |
| 7.23 | DnaJ (Hsp40) homolog, subfamily C, member 1 | 2.93 | 11.71 | 0.13 | 0.003876 |
| 7.21 | Fc receptor, IgG, low affinity IV | 3.73 | 10.82 | 0.06 | 0.000704 |
| 7.21 | Rab40c, member RAS oncogene family | 3.48 | 11.06 | 0.08 | 0.001262 |
| 7.14 | excision repair cross-complementing rodent repair deficiency, complementation group 5 | 3.40 | 11.02 | 0.08 | 0.001408 |
| 7.13 | ash1 (absent, small, or homeotic)-like (Drosophila) | 4.03 | 10.33 | 0.04 | 0.000257 |
| 7.13 | Ngfi-A binding protein 1 | 3.33 | 11.06 | 0.09 | 0.001622 |
| 7.10 | synaptic nuclear envelope 1 | 3.54 | 10.77 | 0.07 | 0.000971 |
| 7.09 | chromodomain helicase DNA binding protein 1 | 3.30 | 11.03 | 0.09 | 0.001700 |
| 7.07 | syntrophin, acidic 1 | 3.32 | 10.97 | 0.09 | 0.001589 |
| 7.07 | solute carrier family 38, member 9 | 2.85 | 11.46 | 0.14 | 0.003948 |
| 7.07 | tetratricopeptide repeat domain 14 | 2.96 | 11.34 | 0.13 | 0.003266 |
| 7.07 | peroxisomal delta3, delta2-enoyl-Coenzyme A isomerase | 3.82 | 10.42 | 0.05 | 0.000443 |
| 7.06 | RAS protein activator like 2 | 2.96 | 11.33 | 0.13 | 0.003230 |
| 7.05 | transformation/transcription domain-associated protein | 3.09 | 11.15 | 0.11 | 0.002473 |
| 7.01 | CUG triplet repeat, RNA binding protein 2 | 3.20 | 10.96 | 0.10 | 0.001913 |
| 6.98 | opioid binding protein/cell adhesion molecule-like | 3.19 | 10.91 | 0.10 | 0.001906 |
| 6.96 | WNK lysine deficient protein kinase 1 | 3.62 | 10.41 | 0.06 | 0.000662 |
| 6.96 | ribosomal RNA processing 1 homolog B (S. cerevisiae) | 3.22 | 10.83 | 0.09 | 0.001752 |
| 6.94 | hypothetical protein LOC632209; RIKEN cDNA C030030A07 gene | 3.57 | 10.42 | 0.06 | 0.000735 |
| 6.92 | ribosomal protein L23 | 3.11 | 10.88 | 0.10 | 0.002158 |
| 6.89 | syndecan binding protein (syntenin) 2 | 3.44 | 10.45 | 0.07 | 0.000954 |
| 6.89 | synapsin III | 3.72 | 10.15 | 0.05 | 0.000443 |
| 6.86 | amyloid beta (A4) precursor protein | 4.12 | 9.66 | 0.02 | 0.000106 |
| 6.85 | regulator of G-protein signalling 9 binding protein | 2.94 | 10.92 | 0.12 | 0.002868 |
| 6.84 | cation channel, sperm associated 2 | 3.83 | 9.94 | 0.04 | 0.000294 |
| 6.83 | thymidine kinase 2, mitochondrial | 3.03 | 10.78 | 0.11 | 0.002357 |
| 6.81 | cytochrome P450, family 3, subfamily a, polypeptide 13 | 3.12 | 10.63 | 0.10 | 0.001895 |
| 6.78 | glucosidase, alpha; neutral C | 3.95 | 9.70 | 0.03 | 0.000175 |
| 6.78 | fragile histidine triad gene | 3.83 | 9.82 | 0.04 | 0.000265 |
| 6.77 | DNA segment, Chr 19, Wayne State University 162, expressed | 2.59 | 11.13 | 0.15 | 0.005095 |
| 6.75 | coiled-coil domain containing 6 | 3.15 | 10.48 | 0.09 | 0.001647 |
| 6.73 | centrosomal protein 97 | 3.50 | 10.05 | 0.06 | 0.000656 |
| 6.73 | proteasome (prosome, macropain) 26S subunit, non-ATPase, 1 | 2.82 | 10.79 | 0.13 | 0.003279 |
| 6.66 | cadherin 19, type 2 | 4.70 | 8.67 | 0.00 | 0.000003 |
| 6.64 | WNK lysine deficient protein kinase 1 | 3.30 | 10.09 | 0.07 | 0.001003 |
| 6.63 | UDP-N-acteylglucosamine pyrophosphorylase 1-like 1 | 2.73 | 10.67 | 0.13 | 0.003545 |
| 6.61 | AF4/FMR2 family, member 1 | 3.19 | 10.16 | 0.08 | 0.001308 |
| 6.61 | zinc finger protein 354A | 2.75 | 10.63 | 0.13 | 0.003425 |
| 6.57 | zinc finger, CCHC domain containing 7 | 2.81 | 10.47 | 0.12 | 0.002930 |
| 6.56 | clathrin, heavy polypeptide (Hc) | 2.61 | 10.66 | 0.14 | 0.004266 |
| 6.54 | similar to TSP50; testes-specific protease 50 | 4.26 | 8.87 | 0.01 | 0.000024 |
| 6.52 | UV radiation resistance associated gene | 3.45 | 9.69 | 0.05 | 0.000558 |
| 6.49 | polymerase (DNA directed), delta 1, catalytic subunit | 3.14 | 9.94 | 0.08 | 0.001257 |
| 6.49 | protocadherin beta 16 | 2.78 | 10.32 | 0.12 | 0.002858 |
| 6.48 | arrestin domain containing 2 | 3.77 | 9.25 | 0.03 | 0.000173 |
| 6.45 | prickle homolog 3 (Drosophila) | 3.26 | 9.74 | 0.07 | 0.000876 |
| 6.40 | phosphodiesterase 4D interacting protein (myomegalin) | 2.77 | 10.17 | 0.12 | 0.002739 |
| 6.40 | integrin alpha FG-GAP repeat containing 1 | 3.16 | 9.74 | 0.07 | 0.001064 |
| 6.33 | predicted gene 7265 | 2.45 | 10.36 | 0.15 | 0.004829 |
| 6.33 | coiled coil domain containing 88A | 2.98 | 9.79 | 0.09 | 0.001562 |
| 6.30 | nuclear factor of kappa light polypeptide gene enhancer in B-cells 1, p105 | 3.38 | 9.31 | 0.05 | 0.000488 |
| 6.23 | similar to Epidermal growth factor-containing fibulin-like extracellular matrix protein 1 | 3.52 | 9.02 | 0.04 | 0.000269 |
| 6.19 | protein kinase C substrate 80K-H | 3.17 | 9.31 | 0.06 | 0.000784 |
| 6.18 | death associated protein kinase 1 | 2.80 | 9.68 | 0.10 | 0.002072 |
| 6.15 | splicing factor, arginine/serine-rich 2, interacting protein | 3.40 | 8.97 | 0.04 | 0.000348 |
| 6.13 | DCP1 decapping enzyme homolog A (S. cerevisiae) | 4.02 | 8.30 | 0.01 | 0.000022 |
| 6.13 | solute carrier family 11 (proton-coupled divalent metal ion transporters), member 1 | 2.75 | 9.63 | 0.11 | 0.002205 |
| 6.13 | apoptosis antagonizing transcription factor | 2.41 | 9.98 | 0.14 | 0.004524 |
| 6.11 | phospholipase A2, group XVI | 3.95 | 8.31 | 0.01 | 0.000028 |
| 6.07 | endoplasmic reticulum (ER) to nucleus signalling 1 | 3.18 | 9.05 | 0.06 | 0.000634 |
| 6.07 | neuralized homolog 1A (Drosophila); similar to neuralized 1 | 3.15 | 9.08 | 0.06 | 0.000691 |
| 6.07 | ribonucleoprotein, PTB-binding 2 | 2.68 | 9.58 | 0.11 | 0.002459 |
| 6.04 | cyclin Y; similar to cyclin fold protein 1 | 3.55 | 8.58 | 0.03 | 0.000151 |
| 6.04 | mechanistic target of rapamycin (serine/threonine kinase) | 2.42 | 9.78 | 0.14 | 0.004110 |
| 6.01 | family with sequence similarity 176, member A | 2.39 | 9.75 | 0.14 | 0.004251 |
| 5.99 | cell division cycle 2-like 5 (cholinesterase-related cell division controller) | 3.40 | 8.65 | 0.04 | 0.000252 |
| 5.92 | ArfGAP with RhoGAP domain, ankyrin repeat and PH domain 3 | 3.38 | 8.53 | 0.03 | 0.000233 |
| 5.91 | predicted gene 5747; RIKEN cDNA 1810026J23 gene | 2.91 | 8.99 | 0.07 | 0.001110 |
| 5.90 | OTU domain containing 7B | 2.39 | 9.53 | 0.14 | 0.003947 |
| 5.89 | ankyrin repeat and SOCS box-containing 8 | 2.82 | 9.05 | 0.08 | 0.001397 |
| 5.89 | aftiphilin | 3.69 | 8.13 | 0.02 | 0.000054 |
| 5.88 | amiloride-sensitive cation channel 1, neuronal (degenerin) | 2.37 | 9.52 | 0.14 | 0.004051 |
| 5.85 | methyl CpG binding protein 2 | 2.49 | 9.32 | 0.12 | 0.003028 |
| 5.85 | phospholipase D family, member 4 | 2.46 | 9.36 | 0.13 | 0.003241 |
| 5.83 | tryptophanyl tRNA synthetase 2 (mitochondrial) | 2.62 | 9.15 | 0.10 | 0.002169 |
| 5.82 | ankyrin 2, brain | 3.01 | 8.69 | 0.06 | 0.000702 |
| 5.77 | mitochondrial translational release factor 1-like | 3.02 | 8.59 | 0.06 | 0.000635 |
| 5.74 | annexin A4 | 3.27 | 8.27 | 0.03 | 0.000241 |
| 5.72 | predicted gene 6158; CCR4-NOT transcription complex, subunit 1 | 2.21 | 9.35 | 0.15 | 0.004972 |
| 5.71 | phosphopantothenoylcysteine decarboxylase | 3.37 | 8.11 | 0.03 | 0.000150 |
| 5.70 | chemokine (C-C motif) ligand 4 | 2.35 | 9.16 | 0.13 | 0.003638 |
| 5.69 | RAR-related orphan receptor gamma | 3.28 | 8.16 | 0.03 | 0.000209 |
| 5.69 | REV1 homolog (S. cerevisiae) | 2.47 | 9.01 | 0.12 | 0.002712 |
| 5.68 | CD209b antigen | 3.77 | 7.61 | 0.01 | 0.000016 |
| 5.67 | HMG box domain containing 3 | 3.27 | 8.13 | 0.03 | 0.000204 |
| 5.64 | lipin 1 | 2.39 | 8.98 | 0.12 | 0.003074 |
| 5.60 | ArfGAP with dual PH domains 2 | 2.45 | 8.85 | 0.11 | 0.002576 |
| 5.60 | RIKEN cDNA 9630025I21 gene | 2.69 | 8.59 | 0.08 | 0.001357 |
| 5.49 | MLX interacting protein-like | 2.62 | 8.45 | 0.08 | 0.001442 |
| 5.49 | vacuolar protein sorting 13A (yeast) | 2.62 | 8.44 | 0.08 | 0.001435 |
| 5.49 | asparaginase homolog (S. cerevisiae) | 2.18 | 8.90 | 0.14 | 0.004321 |
| 5.48 | fibroblast growth factor 7 | 2.41 | 8.65 | 0.11 | 0.002539 |
| 5.48 | potassium inwardly-rectifying channel, subfamily K, member 6 | 3.35 | 7.65 | 0.02 | 0.000084 |
| 5.48 | predicted gene 9895 | 3.22 | 7.79 | 0.03 | 0.000156 |
| 5.48 | tetratricopeptide repeat domain 3 | 2.64 | 8.39 | 0.08 | 0.001339 |
| 5.47 | A kinase (PRKA) anchor protein 8 | 2.50 | 8.53 | 0.10 | 0.001953 |
| 5.42 | importin 7 | 3.12 | 7.77 | 0.03 | 0.000209 |
| 5.41 | sal-like 4 (Drosophila) | 2.97 | 7.90 | 0.04 | 0.000383 |
| 5.40 | integrin alpha V | 3.22 | 7.63 | 0.02 | 0.000127 |
| 5.40 | intermediate filament family orphan 2 | 2.26 | 8.64 | 0.13 | 0.003370 |
| 5.40 | predicted gene 11428 | 2.14 | 8.75 | 0.14 | 0.004393 |
| 5.40 | similar to CDNA sequence AK129341; cDNA sequence AK129341 | 2.58 | 8.29 | 0.08 | 0.001422 |
| 5.34 | uroplakin 1B | 2.36 | 8.41 | 0.11 | 0.002476 |
| 5.33 | testis expressed gene 11 | 3.40 | 7.29 | 0.02 | 0.000038 |
| 5.33 | cleavage and polyadenylation factor subunit homolog (S. cerevisiae) | 2.05 | 8.72 | 0.15 | 0.005079 |
| 5.33 | GTPase activating RANGAP domain-like 1 | 2.11 | 8.64 | 0.14 | 0.004379 |
| 5.32 | splicing factor, arginine/serine-rich 12 | 3.04 | 7.65 | 0.03 | 0.000235 |
| 5.28 | neogenin | 2.83 | 7.80 | 0.05 | 0.000513 |
| 5.27 | guanine nucleotide binding protein, alpha q polypeptide | 2.48 | 8.14 | 0.09 | 0.001615 |
| 5.27 | family with sequence similarity 82, member B | 2.84 | 7.75 | 0.05 | 0.000466 |
| 5.26 | hepatitis A virus cellular receptor 2 | 2.72 | 7.87 | 0.06 | 0.000741 |
| 5.25 | radical S-adenosyl methionine domain containing 2 | 2.48 | 8.09 | 0.09 | 0.001564 |
| 5.23 | interleukin enhancer binding factor 2 | 2.68 | 7.85 | 0.06 | 0.000809 |
| 5.20 | telomeric repeat binding factor 1 | 2.04 | 8.47 | 0.15 | 0.004677 |
| 5.18 | unc-5 homolog C (C. elegans) | 2.52 | 7.91 | 0.08 | 0.001248 |
| 5.18 | fat mass and obesity associated | 2.09 | 8.36 | 0.14 | 0.004048 |
| 5.15 | coiled-coil domain containing 88B | 2.75 | 7.60 | 0.05 | 0.000523 |
| 5.14 | phenylalanyl-tRNA synthetase, alpha subunit | 2.13 | 8.24 | 0.13 | 0.003507 |
| 5.14 | integrator complex subunit 3 | 2.97 | 7.35 | 0.03 | 0.000199 |
| 5.11 | ligand dependent nuclear receptor corepressor | 2.51 | 7.77 | 0.07 | 0.001138 |
| 5.11 | cofilin 1, non-muscle; similar to Cofilin-1 (Cofilin, non-muscle isoform); predicted gene 6180 | 2.34 | 7.96 | 0.10 | 0.001956 |
| 5.09 | helicase with zinc finger domain | 2.10 | 8.17 | 0.13 | 0.003616 |
| 5.09 | pleckstrin homology domain containing, family A member 5 | 2.47 | 7.78 | 0.08 | 0.001266 |
| 5.06 | ADP-ribosylation factor-like 11 | 2.20 | 8.01 | 0.12 | 0.002726 |
| 5.06 | LIM domain containing preferred translocation partner in lipoma | 2.21 | 8.00 | 0.11 | 0.002635 |
| 5.06 | zinc finger and BTB domain containing 39 | 2.00 | 8.22 | 0.14 | 0.004492 |
| 5.06 | golgi associated, gamma adaptin ear containing, ARF binding protein 2 | 2.11 | 8.09 | 0.13 | 0.003434 |
| 5.06 | hypothetical protein LOC100044280; zinc finger protein 758 | 2.01 | 8.20 | 0.14 | 0.004410 |
| 5.02 | CD14 antigen | 2.53 | 7.57 | 0.07 | 0.000916 |
| 5.00 | RIKEN cDNA E130112N10 gene | 2.78 | 7.26 | 0.04 | 0.000326 |
| 4.98 | glycosyltransferase-like domain containing 1 | 2.10 | 7.95 | 0.13 | 0.003283 |
| 4.96 | nuclear factor, erythroid derived 2, like 2 | 1.91 | 8.10 | 0.15 | 0.005045 |
| 4.93 | membrane associated guanylate kinase, WW and PDZ domain containing 2 | 2.75 | 7.16 | 0.04 | 0.000318 |
| 4.91 | nucleoporin like 1 | 1.99 | 7.90 | 0.14 | 0.003924 |
| 4.91 | sorbin and SH3 domain containing 1 | 1.93 | 7.97 | 0.14 | 0.004569 |
| 4.85 | TBC1 domain family, member 5 | 2.17 | 7.60 | 0.11 | 0.002297 |
| 4.84 | serine/threonine kinase 38 | 2.84 | 6.89 | 0.03 | 0.000165 |
| 4.84 | rotatin | 2.57 | 7.17 | 0.05 | 0.000568 |
| 4.84 | RIKEN cDNA 1110057K04 gene | 1.89 | 7.88 | 0.15 | 0.004794 |
| 4.81 | MAP/microtubule affinity-regulating kinase 3 | 2.13 | 7.56 | 0.11 | 0.002440 |
| 4.81 | Kruppel-like factor 4 (gut) | 2.63 | 7.03 | 0.04 | 0.000407 |
| 4.77 | cullin 5 | 2.19 | 7.40 | 0.10 | 0.001890 |
| 4.72 | nuclear respiratory factor 1 | 2.85 | 6.63 | 0.02 | 0.000108 |
| 4.71 | serglycin | 2.01 | 7.49 | 0.12 | 0.003070 |
| 4.71 | niacin receptor 1 | 2.38 | 7.09 | 0.07 | 0.000902 |
| 4.70 | phospholipase A2, activating protein | 1.81 | 7.67 | 0.15 | 0.005090 |
| 4.69 | protocadherin beta 12 | 2.46 | 6.97 | 0.06 | 0.000641 |
| 4.62 | cytokine receptor-like factor 3 | 2.30 | 6.99 | 0.07 | 0.001028 |
| 4.62 | centrosomal protein 120 | 2.03 | 7.27 | 0.11 | 0.002575 |
| 4.59 | engulfment and cell motility 1, ced-12 homolog (C. elegans) | 2.05 | 7.19 | 0.11 | 0.002293 |
| 4.58 | periplakin | 2.56 | 6.64 | 0.04 | 0.000311 |
| 4.57 | molybdenum cofactor synthesis 1 | 2.05 | 7.16 | 0.11 | 0.002287 |
| 4.53 | RIKEN cDNA 3110021N24 gene | 2.19 | 6.92 | 0.08 | 0.001304 |
| 4.45 | transducin-like enhancer of split 4, homolog of Drosophila E(spl) | 1.92 | 7.05 | 0.12 | 0.002868 |
| 4.43 | hypothetical A830091E24 | 2.19 | 6.71 | 0.07 | 0.001111 |
| 4.42 | WW domain binding protein 2 | 2.24 | 6.65 | 0.07 | 0.000887 |
| 4.36 | peptide YY | 1.94 | 6.83 | 0.11 | 0.002387 |
| 4.29 | fermitin family homolog 1 (Drosophila) | 1.67 | 6.98 | 0.15 | 0.004866 |
| 4.22 | pancreatic lipase | 1.85 | 6.65 | 0.11 | 0.002597 |
| 4.21 | neuroligin 1 | 1.97 | 6.51 | 0.09 | 0.001718 |
| 4.21 | protocadherin beta 9 | 2.61 | 5.84 | 0.02 | 0.000071 |
| 4.16 | syntaxin binding protein 3A | 1.67 | 6.71 | 0.14 | 0.004227 |
| 4.08 | arylsulfatase J | 2.13 | 6.07 | 0.06 | 0.000676 |
| 4.00 | Snf2-related CREBBP activator protein | 1.62 | 6.43 | 0.14 | 0.004073 |
| 4.00 | RIKEN cDNA 4922501C03 gene | 1.73 | 6.31 | 0.12 | 0.002865 |
| 3.99 | helicase (DNA) B | 1.98 | 6.04 | 0.07 | 0.001087 |
| 3.90 | peptidoglycan recognition protein 1; similar to peptidoglycan recognition protein | 1.55 | 6.31 | 0.14 | 0.004423 |
| 3.90 | formin 2 | 1.95 | 5.88 | 0.07 | 0.001026 |
| 3.88 | pleckstrin homology domain interacting protein | 2.06 | 5.73 | 0.05 | 0.000562 |
| 3.87 | CD84 antigen | 1.55 | 6.24 | 0.14 | 0.004274 |
| 3.86 | dynamin 3, opposite strand | 1.52 | 6.26 | 0.15 | 0.004655 |
| 3.86 | hydroxysteroid (17-beta) dehydrogenase 1 | 1.57 | 6.21 | 0.14 | 0.003990 |
| 3.80 | RIKEN cDNA 1700029G01 gene | 1.61 | 6.04 | 0.13 | 0.003240 |
| 3.77 | EGF-like repeats and discoidin I-like domains 3 | 1.57 | 6.03 | 0.13 | 0.003562 |
| 3.73 | proteasome (prosome, macropain) inhibitor subunit 1 | 2.22 | 5.26 | 0.03 | 0.000137 |
| 3.68 | olfactory receptor 380; olfactory receptor 75, pseudogene 1 | 1.46 | 5.95 | 0.14 | 0.004474 |
| 3.63 | MAS-related GPR, member G | 1.85 | 5.44 | 0.07 | 0.000863 |
| 3.62 | gene trap locus F3b | 2.23 | 5.02 | 0.02 | 0.000077 |
| 3.60 | tensin like C1 domain-containing phosphatase | 1.67 | 5.56 | 0.10 | 0.001840 |
| 3.59 | CD209f antigen | 1.59 | 5.64 | 0.11 | 0.002492 |
| 3.57 | RIKEN cDNA E030030I06 gene | 1.45 | 5.73 | 0.14 | 0.004001 |
| 3.56 | phosphatidylserine decarboxylase, pseudogene 3 | 1.43 | 5.74 | 0.14 | 0.004188 |
| 3.56 | calcium binding protein 4 | 1.93 | 5.22 | 0.05 | 0.000461 |
| 3.51 | lymphatic vessel endothelial hyaluronan receptor 1 | 1.48 | 5.59 | 0.13 | 0.003339 |
| 3.47 | WNT1 inducible signaling pathway protein 1 | 1.67 | 5.30 | 0.08 | 0.001394 |
| 3.46 | histocompatibility (minor) HA-1 | 1.49 | 5.46 | 0.12 | 0.002884 |
| 3.46 | arachidonate 8-lipoxygenase | 1.54 | 5.42 | 0.11 | 0.002432 |
| 3.40 | nuclear antigen Sp100 | 2.07 | 4.75 | 0.02 | 0.000096 |
| 3.38 | budding uninhibited by benzimidazoles 1 homolog (S. cerevisiae) | 1.61 | 5.17 | 0.09 | 0.001492 |
| 3.35 | predicted gene 4831 | 1.47 | 5.26 | 0.11 | 0.002660 |
| 3.34 | tumor necrosis factor, alpha-induced protein 3 | 1.32 | 5.41 | 0.15 | 0.004628 |
| 3.27 | moesin | 1.42 | 5.15 | 0.12 | 0.002809 |
| 3.22 | WD repeat domain 67 | 1.34 | 5.13 | 0.13 | 0.003531 |
| 3.20 | catenin (cadherin associated protein), alpha 2 | 1.27 | 5.18 | 0.14 | 0.004566 |
| 3.20 | SET and MYND domain containing 3 | 1.39 | 5.05 | 0.12 | 0.002851 |
| 3.19 | olfactory receptor 908 | 1.39 | 5.01 | 0.12 | 0.002729 |
| 3.18 | Janus kinase 2 | 2.01 | 4.36 | 0.02 | 0.000050 |
| 3.07 | 2'-5' oligoadenylate synthetase 2 | 1.21 | 4.96 | 0.15 | 0.004655 |
| 3.06 | leucine-rich repeats and immunoglobulin-like domains 3 | 1.31 | 4.84 | 0.12 | 0.003015 |
| 3.03 | immunoglobulin kappa chain variable 19 (V19)-15; similar to immunoglobulin kappa-chain | 1.35 | 4.73 | 0.11 | 0.002441 |
| 3.02 | mucosa associated lymphoid tissue lymphoma translocation gene 1 | 1.44 | 4.64 | 0.09 | 0.001555 |
| 2.98 | UbiA prenyltransferase domain containing 1 | 1.18 | 4.81 | 0.14 | 0.004564 |
| 2.96 | predicted gene 5631 | 1.56 | 4.39 | 0.06 | 0.000637 |
| 2.96 | tumor necrosis factor receptor superfamily, member 1b | 1.23 | 4.71 | 0.13 | 0.003537 |
| 2.96 | leptin receptor | 1.22 | 4.72 | 0.13 | 0.003750 |
| 2.94 | growth hormone secretagogue receptor | 1.21 | 4.69 | 0.13 | 0.003718 |
| 2.93 | predicted gene 10880 | 1.31 | 4.57 | 0.11 | 0.002370 |
| 2.90 | baculoviral IAP repeat-containing 6 | 1.64 | 4.17 | 0.04 | 0.000281 |
| 2.85 | Protein C21orf63 homolog precursor | 1.17 | 4.56 | 0.14 | 0.003907 |
| 2.83 | mixed lineage kinase domain-like | 2.05 | 3.62 | 0.00 | 0.000002 |
| 2.81 | PHD finger protein 12 | 1.20 | 4.45 | 0.12 | 0.003156 |
| 2.81 | protein tyrosine phosphatase, non-receptor type 14 | 1.11 | 4.53 | 0.14 | 0.004558 |
| 2.80 | tumor necrosis factor, alpha-induced protein 2 | 1.20 | 4.42 | 0.12 | 0.003026 |
| 2.77 | olfactory receptor 1066 | 1.20 | 4.36 | 0.12 | 0.002824 |
| 2.74 | sushi, von Willebrand factor type A, EGF and pentraxin domain containing 1 | 1.41 | 4.08 | 0.06 | 0.000808 |
| 2.71 | cathepsin O | 1.37 | 4.07 | 0.07 | 0.000936 |
| 2.63 | ADAMTS-like 3 | 1.13 | 4.15 | 0.12 | 0.002995 |
| 2.59 | serine (or cysteine) peptidase inhibitor, clade A, member 1C | 1.12 | 4.09 | 0.12 | 0.002989 |
| 2.54 | RIKEN cDNA 1600015I10 gene | 1.07 | 4.03 | 0.13 | 0.003335 |
| 2.52 | neurobeachin like 1 | 1.40 | 3.66 | 0.04 | 0.000365 |
| 2.51 | G protein-coupled receptor 132 | 1.50 | 3.54 | 0.03 | 0.000139 |
| 2.47 | major facilitator superfamily domain containing 7A | 1.29 | 3.66 | 0.06 | 0.000682 |
| 2.41 | C3 and PZP-like, alpha-2-macroglobulin domain containing 8 | 1.06 | 3.77 | 0.11 | 0.002581 |
| 2.36 | brain and reproductive organ-expressed protein | 1.14 | 3.58 | 0.08 | 0.001340 |
| 2.35 | RIKEN cDNA 2610507B11 gene | 0.94 | 3.77 | 0.14 | 0.004332 |
| 2.32 | RIKEN cDNA 1700085C21 gene | 0.95 | 3.71 | 0.13 | 0.003870 |
| 2.32 | RIKEN cDNA 5830409B07 gene | 1.13 | 3.53 | 0.08 | 0.001340 |
| 2.29 | dipeptidase 2 | 0.96 | 3.64 | 0.13 | 0.003467 |
| 2.28 | zinc finger protein 128 | 1.21 | 3.36 | 0.05 | 0.000589 |
| 2.27 | fragile X mental retardation syndrome 1 homolog | 0.89 | 3.66 | 0.15 | 0.004777 |
| 2.26 | ankyrin repeat domain 53 | 0.91 | 3.62 | 0.14 | 0.004195 |
| 2.22 | cDNA sequence BC049349 | 0.88 | 3.59 | 0.15 | 0.004615 |
| 2.12 | genetic suppressor element 1 | 0.87 | 3.37 | 0.13 | 0.003758 |
| 2.03 | WD repeat domain 60 | 0.84 | 3.24 | 0.13 | 0.003765 |
| 1.94 | solute carrier family 1 (glial high affinity glutamate transporter), member 2 | 0.86 | 3.02 | 0.11 | 0.002469 |
| 1.91 | hypothetical protein LOC100046852 | 0.80 | 3.04 | 0.13 | 0.003607 |
| 1.78 | predicted gene 4801 | 0.76 | 2.81 | 0.12 | 0.003101 |
| 1.66 | serine (or cysteine) peptidase inhibitor, clade B, member 9f | 0.76 | 2.57 | 0.10 | 0.002084 |
| 1.58 | inner membrane protein, mitochondrial | 0.71 | 2.45 | 0.11 | 0.002253 |
