# Supplementary material for: Voluntary Wheel Running Reverses Age-Induced Changes in Hippocampal Gene Expression
Source: PLoS One. 2011 Aug 8;6(8):e22654. doi: 10.1371/journal.pone.0022654 (PMC3152565; doi:10.1371/journal.pone.0022654)
Supplement: Table S3 — Exercise-induced changes in gene expression in the hippocampus. Columns list the percent change in gene expression in runners (collapsed across age) compared to sedentary mice ±95% confidence intervals for an individual gene. Positive values indicate the percent increase in expression and negative values indicate a decrease in expression. FDRs are expressed as percentage scores. (DOC) [file pone.0022654.s003.doc]

| **Supplementary Table S3.** | |  | |  |  | |  |
| --- | --- | --- | --- | --- | --- | --- | --- |
| Exercise-induced changes in gene expression in the hippocampus  ***Downregulated*** | | **95% confidence intervals** | | |  | | |
| **Percent change** | **Gene name** | **Lower** | **Upper** | | **FDR** | **P value** | |
| -29.67 | dual specificity phosphatase 1 | -15.59 | -41.40 | | 0.16 | 0.001489 | |
| -26.53 | transmembrane protein 114 | -17.42 | -34.64 | | 0.05 | 0.000077 | |
| -21.71 | insulin-like growth factor binding protein 5 | -16.05 | -26.99 | | 0.02 | 0.000003 | |
| -21.67 | WD repeat domain 6 | -11.17 | -30.93 | | 0.15 | 0.001409 | |
| -20.83 | pregnancy upregulated non-ubiquitously expressed CaM kinase | -11.97 | -28.80 | | 0.11 | 0.000467 | |
| -20.26 | predicted gene 15453; RNA binding motif protein 3 | -9.63 | -29.63 | | 0.18 | 0.002476 | |
| -19.50 | solute carrier family 38, member 2 | -11.83 | -26.50 | | 0.08 | 0.000220 | |
| -18.59 | protocadherin 21 | -11.53 | -25.09 | | 0.07 | 0.000152 | |
| -18.27 | amylase 2a2, pancreatic | -9.56 | -26.15 | | 0.14 | 0.001143 | |
| -18.25 | cold inducible RNA binding protein | -10.51 | -25.32 | | 0.10 | 0.000422 | |
| -17.92 | cytoglobin | -11.15 | -24.17 | | 0.07 | 0.000139 | |
| -17.25 | actin, alpha 2, smooth muscle, aorta | -10.42 | -23.56 | | 0.08 | 0.000216 | |
| -17.22 | src homology 2 domain-containing transforming protein D | -8.47 | -25.14 | | 0.17 | 0.001838 | |
| -17.02 | Von Willebrand factor homolog | -7.76 | -25.35 | | 0.19 | 0.003013 | |
| -16.37 | CDC-like kinase 1 | -9.49 | -22.73 | | 0.10 | 0.000367 | |
| -15.84 | activin receptor IIB | -10.59 | -20.78 | | 0.04 | 0.000033 | |
| -15.29 | B-cell translocation gene 1, anti-proliferative | -8.70 | -21.40 | | 0.11 | 0.000441 | |
| -15.03 | zinc finger protein 36, C3H type-like 1 | -8.08 | -21.46 | | 0.13 | 0.000818 | |
| -14.05 | Cbp/p300-interacting transactivator, with Glu/Asp-rich carboxy-terminal domain, 2 | -7.19 | -20.41 | | 0.15 | 0.001250 | |
| -14.05 | regulator of calcineurin 3 | -5.82 | -21.56 | | 0.22 | 0.004746 | |
| -13.89 | apoptosis-inducing factor, mitochondrion-associated 3 | -6.25 | -20.90 | | 0.19 | 0.003060 | |
| -13.72 | DNA segment, Chr 16, ERATO Doi 472, expressed | -7.75 | -19.31 | | 0.11 | 0.000462 | |
| -13.42 | small nuclear ribonucleoprotein B | -8.56 | -18.03 | | 0.05 | 0.000077 | |
| -13.36 | SRY-box containing gene 9 | -7.28 | -19.05 | | 0.12 | 0.000684 | |
| -13.25 | solute carrier family 6 (neurotransmitter transporter), member 15 | -5.85 | -20.07 | | 0.20 | 0.003383 | |
| -13.11 | CDC like kinase 4 | -8.63 | -17.36 | | 0.05 | 0.000041 | |
| -13.10 | enoyl Coenzyme A hydratase domain containing 2 | -6.86 | -18.93 | | 0.13 | 0.000991 | |
| -13.06 | lamin A | -8.13 | -17.73 | | 0.06 | 0.000116 | |
| -12.97 | heat shock protein, alpha-crystallin-related, B6 | -6.94 | -18.61 | | 0.13 | 0.000804 | |
| -12.96 | predicted gene 347 | -5.41 | -19.91 | | 0.22 | 0.004506 | |
| -12.89 | Complement component 4B | -5.77 | -19.48 | | 0.19 | 0.003111 | |
| -12.83 | glypican 3 | -6.44 | -18.79 | | 0.15 | 0.001425 | |
| -12.72 | zinc finger, FYVE domain containing 21 | -8.87 | -16.41 | | 0.03 | 0.000010 | |
| -12.72 | RIKEN cDNA 1500012F01 gene | -6.07 | -18.90 | | 0.17 | 0.002066 | |
| -12.71 | dihydrouridine synthase 4-like (S. cerevisiae) | -5.57 | -19.31 | | 0.20 | 0.003481 | |
| -12.32 | uridine phosphorylase 1 | -7.25 | -17.12 | | 0.09 | 0.000267 | |
| -12.31 | phosphoinositide-3-kinase interacting protein 1 | -8.05 | -16.37 | | 0.05 | 0.000045 | |
| -12.23 | FERM, RhoGEF (Arhgef) and pleckstrin domain protein 1 (chondrocyte-derived) | -5.89 | -18.14 | | 0.17 | 0.001916 | |
| -12.20 | suppression of tumorigenicity 5 | -5.65 | -18.29 | | 0.18 | 0.002475 | |
| -12.14 | sperm associated antigen 5 | -5.52 | -18.30 | | 0.18 | 0.002787 | |
| -11.93 | axin2 | -6.28 | -17.23 | | 0.13 | 0.000919 | |
| -11.89 | pyruvate dehydrogenase kinase, isoenzyme 4 | -7.69 | -15.90 | | 0.05 | 0.000056 | |
| -11.71 | sema domain, immunoglobulin domain, transmembrane and short cytoplasmic domain, 4B | -5.75 | -17.30 | | 0.16 | 0.001652 | |
| -11.56 | patched homolog 1 | -5.64 | -17.11 | | 0.16 | 0.001709 | |
| -11.53 | zyxin | -6.31 | -16.46 | | 0.12 | 0.000619 | |
| -11.43 | SET and MYND domain containing 3 | -6.85 | -15.78 | | 0.08 | 0.000197 | |
| -11.41 | dihydrolipoamide S-succinyltransferase (E2 component of 2-oxo-glutarate complex) | -5.48 | -16.97 | | 0.17 | 0.001911 | |
| -11.38 | leucine-rich repeats and immunoglobulin-like domains 3 | -6.16 | -16.31 | | 0.12 | 0.000692 | |
| -11.15 | coiled-coil and C2 domain containing 1A | -6.13 | -15.90 | | 0.11 | 0.000578 | |
| -11.11 | discs, large homolog 5 (Drosophila) | -4.73 | -17.05 | | 0.21 | 0.003941 | |
| -11.04 | SWI/SNF related, matrix associated, actin dependent regulator of chromatin, subfamily a, 2 | -5.87 | -15.93 | | 0.13 | 0.000820 | |
| -11.00 | trinucleotide repeat containing 6a | -5.20 | -16.45 | | 0.17 | 0.002127 | |
| -10.95 | chromodomain helicase DNA binding protein 7 | -4.84 | -16.67 | | 0.19 | 0.003235 | |
| -10.92 | abhydrolase domain containing 14b | -5.04 | -16.44 | | 0.18 | 0.002497 | |
| -10.91 | AHNAK nucleoprotein (desmoyokin) | -5.75 | -15.78 | | 0.13 | 0.000876 | |
| -10.87 | matrix metallopeptidase 14 (membrane-inserted) | -5.26 | -16.15 | | 0.16 | 0.001791 | |
| -10.82 | similar to mKIAA1021 protein | -5.20 | -16.11 | | 0.17 | 0.001894 | |
| -10.81 | biglycan | -6.22 | -15.17 | | 0.10 | 0.000335 | |
| -10.79 | family with sequence similarity 123, member A | -7.20 | -14.25 | | 0.04 | 0.000028 | |
| -10.79 | growth arrest specific 6 | -5.38 | -15.90 | | 0.15 | 0.001439 | |
| -10.70 | sideroflexin 1 | -4.95 | -16.09 | | 0.18 | 0.002424 | |
| -10.67 | ribosomal protein S3 | -4.51 | -16.42 | | 0.21 | 0.004054 | |
| -10.63 | ribosomal protein S15A | -6.32 | -14.74 | | 0.08 | 0.000216 | |
| -10.53 | predicted gene 7776; proteasome (prosome, macropain) 28 subunit, alpha | -4.51 | -16.16 | | 0.20 | 0.003795 | |
| -10.52 | filamin, beta | -5.05 | -15.68 | | 0.17 | 0.001894 | |
| -10.35 | solute carrier family 6 (neurotransmitter transporter, taurine), member 6 | -6.68 | -13.87 | | 0.05 | 0.000055 | |
| -10.27 | potassium voltage-gated channel, subfamily Q, member 2 | -5.32 | -14.96 | | 0.13 | 0.001024 | |
| -10.26 | insulin-like growth factor I receptor | -5.04 | -15.19 | | 0.16 | 0.001584 | |
| -10.26 | SRY-box containing gene 21 | -5.25 | -15.01 | | 0.14 | 0.001143 | |
| -10.22 | hypothetical protein LOC100045106; V-set and transmembrane domain containing 2B | -4.63 | -15.48 | | 0.18 | 0.002770 | |
| -10.08 | transducer of ErbB-2.1 | -4.09 | -15.70 | | 0.23 | 0.004907 | |
| -9.98 | zinc finger protein 282 | -6.92 | -12.94 | | 0.03 | 0.000010 | |
| -9.94 | GNAS (guanine nucleotide binding protein, alpha stimulating) complex locus | -5.07 | -14.56 | | 0.14 | 0.001161 | |
| -9.93 | polymerase (RNA) I polypeptide A | -4.54 | -15.02 | | 0.18 | 0.002590 | |
| -9.92 | cyclin D2 | -5.50 | -14.12 | | 0.11 | 0.000508 | |
| -9.88 | solute carrier family 48 (heme transporter), member 1 | -4.60 | -14.87 | | 0.18 | 0.002316 | |
| -9.74 | dihydropyrimidinase-like 4 | -4.87 | -14.36 | | 0.15 | 0.001370 | |
| -9.62 | pleckstrin homology domain containing, family M (with RUN domain) member 2 | -5.11 | -13.92 | | 0.13 | 0.000794 | |
| -9.53 | TBC1 domain family, member 9B | -5.25 | -13.63 | | 0.11 | 0.000550 | |
| -9.45 | neuron navigator 1 | -4.50 | -14.15 | | 0.17 | 0.001950 | |
| -9.44 | BCL2 binding component 3 | -4.10 | -14.49 | | 0.20 | 0.003482 | |
| -9.43 | TRIO and F-actin binding protein | -4.27 | -14.32 | | 0.18 | 0.002734 | |
| -9.38 | LIM domain only 2 | -4.91 | -13.63 | | 0.13 | 0.000900 | |
| -9.33 | protein phosphatase 1M | -5.48 | -13.03 | | 0.08 | 0.000248 | |
| -9.33 | RIKEN cDNA 2210013O21 gene | -5.07 | -13.39 | | 0.12 | 0.000619 | |
| -9.25 | KDM1 lysine (K)-specific demethylase 6B | -5.03 | -13.29 | | 0.12 | 0.000627 | |
| -9.17 | kelch domain containing 9 | -5.12 | -13.04 | | 0.11 | 0.000461 | |
| -9.11 | brain protein 16 | -4.97 | -13.07 | | 0.12 | 0.000595 | |
| -8.98 | calpain 5 | -4.40 | -13.34 | | 0.16 | 0.001574 | |
| -8.84 | RIKEN cDNA 1110007L15 gene; predicted gene 3606 | -5.70 | -11.87 | | 0.05 | 0.000053 | |
| -8.83 | solute carrier family 5 (sodium iodide symporter), member 5 | -3.91 | -13.49 | | 0.19 | 0.003079 | |
| -8.76 | calmodulin binding transcription activator 1 | -3.82 | -13.44 | | 0.20 | 0.003335 | |
| -8.73 | mortality factor 4 like 1 | -3.57 | -13.61 | | 0.22 | 0.004638 | |
| -8.67 | DDRGK domain containing 1 | -3.82 | -13.27 | | 0.19 | 0.003143 | |
| -8.60 | kelch-like 21 (Drosophila) | -3.85 | -13.12 | | 0.19 | 0.002871 | |
| -8.55 | TBC1 domain family, member 2 | -3.75 | -13.10 | | 0.19 | 0.003224 | |
| -8.50 | hexamethylene bis-acetamide inducible 1 | -4.57 | -12.26 | | 0.12 | 0.000688 | |
| -8.44 | solute carrier family 25, member 28 | -5.12 | -11.64 | | 0.07 | 0.000149 | |
| -8.41 | enhancer of polycomb homolog 1 (Drosophila) | -5.75 | -10.99 | | 0.03 | 0.000014 | |
| -8.40 | programmed cell death 4 | -3.38 | -13.15 | | 0.23 | 0.004974 | |
| -8.37 | polypyrimidine tract binding protein 1; predicted gene 4900 | -4.04 | -12.50 | | 0.16 | 0.001746 | |
| -8.35 | GM2 ganglioside activator protein | -3.48 | -12.97 | | 0.21 | 0.004202 | |
| -8.31 | coiled-coil domain containing 74A | -4.37 | -12.09 | | 0.13 | 0.000842 | |
| -8.31 | ankyrin repeat and SOCS box-containing 16 | -4.41 | -12.05 | | 0.13 | 0.000781 | |
| -8.31 | period homolog 3 (Drosophila) | -4.33 | -12.11 | | 0.13 | 0.000910 | |
| -8.30 | thyrotroph embryonic factor | -3.95 | -12.46 | | 0.17 | 0.001936 | |
| -8.26 | translocase of outer mitochondrial membrane 6 homolog (yeast) | -3.82 | -12.50 | | 0.18 | 0.002344 | |
| -8.25 | ribosomal protein L22 | -4.61 | -11.75 | | 0.11 | 0.000450 | |
| -8.22 | predicted gene 11889; LUC7-like 2 (S. cerevisiae) | -4.07 | -12.19 | | 0.15 | 0.001439 | |
| -8.20 | zinc finger protein 787 | -4.38 | -11.87 | | 0.13 | 0.000733 | |
| -8.19 | RIKEN cDNA B930041F14 gene | -4.05 | -12.15 | | 0.15 | 0.001439 | |
| -8.19 | complement factor properdin | -3.77 | -12.40 | | 0.18 | 0.002377 | |
| -8.12 | dynamin 1 | -5.03 | -11.12 | | 0.06 | 0.000107 | |
| -8.09 | eukaryotic translation initiation factor 3, subunit F | -3.80 | -12.20 | | 0.17 | 0.002105 | |
| -8.07 | tetratricopeptide repeat domain 14 | -3.80 | -12.15 | | 0.17 | 0.002065 | |
| -8.05 | TBC1 domain family, member 2B | -3.39 | -12.49 | | 0.21 | 0.003975 | |
| -8.04 | pleiomorphic adenoma gene-like 2 | -4.28 | -11.65 | | 0.13 | 0.000744 | |
| -7.96 | cysteine-rich hydrophobic domain 2 | -3.41 | -12.29 | | 0.20 | 0.003642 | |
| -7.95 | cDNA sequence BC031353 | -3.21 | -12.46 | | 0.22 | 0.004866 | |
| -7.94 | CTD (carboxy-terminal domain, RNA polymerase II, polypeptide A) small phosphatase-like | -3.55 | -12.13 | | 0.19 | 0.002856 | |
| -7.92 | mitogen-activated protein kinase kinase kinase kinase 2 | -3.53 | -12.11 | | 0.19 | 0.002926 | |
| -7.91 | Rap guanine nucleotide exchange factor (GEF) 3 | -4.74 | -10.98 | | 0.07 | 0.000177 | |
| -7.90 | oviductal glycoprotein 1 | -4.36 | -11.31 | | 0.11 | 0.000511 | |
| -7.90 | ribosomal protein L24 | -3.21 | -12.36 | | 0.22 | 0.004708 | |
| -7.86 | progressive ankylosis | -4.31 | -11.27 | | 0.11 | 0.000549 | |
| -7.83 | matrix-remodelling associated 7 | -3.47 | -12.01 | | 0.19 | 0.003042 | |
| -7.81 | RIKEN cDNA 2310003H01 gene | -4.32 | -11.18 | | 0.11 | 0.000504 | |
| -7.77 | aryl hydrocarbon receptor nuclear translocator | -4.93 | -10.53 | | 0.05 | 0.000069 | |
| -7.77 | male-specific lethal 2 homolog (Drosophila) | -3.36 | -11.98 | | 0.20 | 0.003440 | |
| -7.75 | protein tyrosine phosphatase, receptor type, f polypeptide , interacting protein, alpha 1 | -3.51 | -11.81 | | 0.18 | 0.002646 | |
| -7.74 | ankyrin repeat domain 39 | -4.73 | -10.66 | | 0.07 | 0.000132 | |
| -7.73 | solute carrier family 22 (organic cation transporter), member 4 | -4.05 | -11.27 | | 0.13 | 0.000869 | |
| -7.67 | bicaudal C homolog 1 (Drosophila) | -3.33 | -11.82 | | 0.20 | 0.003398 | |
| -7.63 | SYF2 homolog, RNA splicing factor (S. cerevisiae) | -4.67 | -10.49 | | 0.07 | 0.000125 | |
| -7.59 | glutathione S-transferase, theta 1 | -3.68 | -11.35 | | 0.16 | 0.001649 | |
| -7.52 | S-phase kinase-associated protein 2 (p45) | -3.37 | -11.50 | | 0.18 | 0.002798 | |
| -7.50 | interferon regulatory factor 2 binding protein 1 | -3.13 | -11.68 | | 0.21 | 0.004151 | |
| -7.48 | predicted gene 4945; predicted gene 9250 | -3.78 | -11.03 | | 0.14 | 0.001178 | |
| -7.48 | retinoblastoma binding protein 4 | -4.20 | -10.65 | | 0.10 | 0.000417 | |
| -7.47 | RIKEN cDNA B230380D07 gene | -3.07 | -11.66 | | 0.22 | 0.004437 | |
| -7.44 | similar to Inhbb protein | -4.50 | -10.29 | | 0.07 | 0.000152 | |
| -7.44 | mutated in colorectal cancers | -3.38 | -11.32 | | 0.18 | 0.002569 | |
| -7.33 | v-rel reticuloendotheliosis viral oncogene homolog A (avian) | -3.40 | -11.10 | | 0.18 | 0.002245 | |
| -7.28 | cDNA sequence BC029214 | -3.21 | -11.18 | | 0.19 | 0.003065 | |
| -7.26 | nucleosome assembly protein 1-like 1 | -3.68 | -10.71 | | 0.14 | 0.001157 | |
| -7.25 | predicted gene 4754; ribosomal protein L7 | -3.21 | -11.12 | | 0.19 | 0.002987 | |
| -7.24 | fat mass and obesity associated | -2.91 | -11.38 | | 0.23 | 0.004940 | |
| -7.24 | nanos homolog 2 (Drosophila) | -4.47 | -9.92 | | 0.06 | 0.000105 | |
| -7.23 | CDC14 cell division cycle 14 homolog B (S. cerevisiae) | -3.70 | -10.63 | | 0.14 | 0.001057 | |
| -7.17 | RAB5B, member RAS oncogene family | -3.42 | -10.78 | | 0.17 | 0.001858 | |
| -7.14 | glutathione peroxidase 2 | -3.11 | -10.99 | | 0.19 | 0.003262 | |
| -7.13 | transcription elongation factor A (SII), 2 | -3.58 | -10.54 | | 0.15 | 0.001229 | |
| -7.05 | small nuclear ribonucleoprotein 48 (U11/U12) | -3.06 | -10.87 | | 0.20 | 0.003340 | |
| -6.99 | gametogenetin binding protein 1; RIKEN cDNA 0610031G08 gene | -3.07 | -10.75 | | 0.19 | 0.003132 | |
| -6.97 | sorting nexin 10 | -3.07 | -10.72 | | 0.19 | 0.003077 | |
| -6.95 | phosphohistidine phosphatase 1 | -2.79 | -10.93 | | 0.23 | 0.004960 | |
| -6.90 | neuralized homolog 1A (Drosophila); similar to neuralized 1 | -4.26 | -9.46 | | 0.06 | 0.000108 | |
| -6.88 | MYC binding protein 2 | -2.88 | -10.71 | | 0.21 | 0.004020 | |
| -6.88 | optineurin | -2.77 | -10.81 | | 0.22 | 0.004868 | |
| -6.86 | hypothetical protein LOC100044213; CWF19-like 2, cell cycle control (S. pombe) | -2.80 | -10.74 | | 0.22 | 0.004536 | |
| -6.78 | molybdenum cofactor synthesis 1 | -4.48 | -9.03 | | 0.04 | 0.000030 | |
| -6.78 | hairy and enhancer of split 5 (Drosophila) | -3.13 | -10.29 | | 0.18 | 0.002278 | |
| -6.75 | similar to ribosomal protein S11 | -2.85 | -10.50 | | 0.21 | 0.003871 | |
| -6.74 | unconventional SNARE in the ER 1 homolog (S. cerevisiae) | -3.47 | -9.89 | | 0.13 | 0.000979 | |
| -6.72 | La ribonucleoprotein domain family, member 6 | -3.28 | -10.04 | | 0.16 | 0.001539 | |
| -6.72 | polymerase (RNA) II (DNA directed) polypeptide D | -2.88 | -10.41 | | 0.20 | 0.003561 | |
| -6.71 | uroporphyrinogen decarboxylase | -2.91 | -10.36 | | 0.20 | 0.003341 | |
| -6.68 | Eph receptor B4 | -3.00 | -10.23 | | 0.18 | 0.002755 | |
| -6.65 | folylpolyglutamyl synthetase | -3.69 | -9.53 | | 0.11 | 0.000468 | |
| -6.65 | PRP19/PSO4 pre-mRNA processing factor 19 homolog (S. cerevisiae) | -3.36 | -9.83 | | 0.14 | 0.001178 | |
| -6.58 | brain-specific angiogenesis inhibitor 1-associated protein 2 | -3.54 | -9.52 | | 0.12 | 0.000643 | |
| -6.56 | regulator of G-protein signalling 9 binding protein | -3.01 | -9.99 | | 0.18 | 0.002389 | |
| -6.52 | aminolevulinate, delta-, dehydratase | -2.72 | -10.16 | | 0.21 | 0.004045 | |
| -6.51 | lipin 1 | -3.01 | -9.88 | | 0.18 | 0.002253 | |
| -6.48 | predicted gene 5750; ribosomal protein L7A | -3.12 | -9.72 | | 0.16 | 0.001688 | |
| -6.36 | polyamine oxidase (exo-N4-amino) | -2.80 | -9.79 | | 0.19 | 0.003067 | |
| -6.32 | forkhead box C1 | -3.47 | -9.08 | | 0.11 | 0.000514 | |
| -6.17 | glypican 5 | -4.12 | -8.17 | | 0.04 | 0.000022 | |
| -6.17 | SAPS domain family, member 3 | -2.50 | -9.69 | | 0.22 | 0.004630 | |
| -6.13 | BAH domain and coiled-coil containing 1 | -3.44 | -8.75 | | 0.10 | 0.000402 | |
| -6.09 | PHD finger protein 3 | -2.77 | -9.30 | | 0.18 | 0.002478 | |
| -6.07 | solute carrier family 19 (sodium/hydrogen exchanger), member 1 | -2.65 | -9.36 | | 0.19 | 0.003173 | |
| -6.06 | prosaposin | -2.54 | -9.45 | | 0.21 | 0.003967 | |
| -6.05 | ectonucleoside triphosphate diphosphohydrolase 2 | -2.67 | -9.31 | | 0.19 | 0.003014 | |
| -5.99 | nuclear VCP-like | -3.29 | -8.61 | | 0.11 | 0.000505 | |
| -5.94 | sortilin-related VPS10 domain containing receptor 2 | -2.60 | -9.18 | | 0.19 | 0.003146 | |
| -5.94 | zinc finger protein 790 | -2.62 | -9.14 | | 0.19 | 0.002994 | |
| -5.93 | polymerase (DNA directed), delta 2, regulatory subunit | -3.48 | -8.32 | | 0.08 | 0.000223 | |
| -5.93 | MTOR associated protein, LST8 homolog (S. cerevisiae) | -3.26 | -8.52 | | 0.11 | 0.000502 | |
| -5.92 | PR domain containing 2, with ZNF domain | -2.56 | -9.16 | | 0.20 | 0.003317 | |
| -5.91 | ribosomal protein L23 | -2.39 | -9.30 | | 0.22 | 0.004661 | |
| -5.87 | predicted gene 5879; similar to 60S ribosomal protein L3 (J1 protein) | -2.52 | -9.12 | | 0.20 | 0.003503 | |
| -5.87 | polymerase (RNA) III (DNA directed) polypeptide E | -3.06 | -8.59 | | 0.13 | 0.000854 | |
| -5.83 | 60S ribosomal protein L3 (J1 protein) | -2.42 | -9.13 | | 0.21 | 0.004172 | |
| -5.82 | elongation factor RNA polymerase II | -3.00 | -8.57 | | 0.13 | 0.000979 | |
| -5.76 | KH domain containing, RNA binding, signal transduction associated 1 | -2.49 | -8.92 | | 0.20 | 0.003320 | |
| -5.75 | FERM domain containing 4B | -2.43 | -8.95 | | 0.20 | 0.003743 | |
| -5.73 | succinate-Coenzyme A ligase, GDP-forming, beta subunit | -2.50 | -8.86 | | 0.19 | 0.003177 | |
| -5.72 | solute carrier family 6 (neurotransmitter transporter), member 17 | -3.26 | -8.13 | | 0.10 | 0.000330 | |
| -5.72 | amiloride-sensitive cation channel 1, neuronal (degenerin) | -2.49 | -8.85 | | 0.19 | 0.003241 | |
| -5.72 | formin-like 3 | -2.38 | -8.94 | | 0.21 | 0.004087 | |
| -5.69 | BCL2-like 2 | -2.58 | -8.71 | | 0.18 | 0.002555 | |
| -5.67 | interleukin enhancer binding factor 3 | -2.31 | -8.91 | | 0.22 | 0.004542 | |
| -5.67 | SREBF chaperone | -2.34 | -8.88 | | 0.21 | 0.004223 | |
| -5.64 | MAD2L1 binding protein | -2.61 | -8.58 | | 0.18 | 0.002219 | |
| -5.59 | protein phosphatase 1A, magnesium dependent, alpha isoform | -2.28 | -8.79 | | 0.22 | 0.004472 | |
| -5.58 | nuclear factor of kappa light polypeptide gene enhancer in B-cells 1, p105 | -2.90 | -8.20 | | 0.13 | 0.000897 | |
| -5.58 | methyl CpG binding protein 2 | -2.47 | -8.60 | | 0.19 | 0.002930 | |
| -5.44 | peroxisomal delta3, delta2-enoyl-Coenzyme A isomerase | -2.45 | -8.34 | | 0.18 | 0.002623 | |
| -5.44 | lectin, galactose binding, soluble 4 | -2.66 | -8.14 | | 0.16 | 0.001496 | |
| -5.39 | peptidyl arginine deiminase, type II; similar to peptidyl arginine deiminase, type II | -2.30 | -8.38 | | 0.20 | 0.003573 | |
| -5.35 | heterogeneous nuclear ribonucleoprotein A0 | -2.51 | -8.11 | | 0.17 | 0.001991 | |
| -5.30 | ATPase, class V, type 10A | -2.61 | -7.93 | | 0.15 | 0.001423 | |
| -5.28 | predicted gene 5747; RIKEN cDNA 1810026J23 gene | -2.49 | -7.98 | | 0.17 | 0.001907 | |
| -5.26 | valyl-tRNA synthetase 2, mitochondrial (putative); similar to valyl-tRNA synthetase 2-like | -2.47 | -7.97 | | 0.17 | 0.001962 | |
| -5.19 | 3-hydroxybutyrate dehydrogenase, type 1 | -2.18 | -8.10 | | 0.20 | 0.003819 | |
| -5.11 | family with sequence similarity 46, member A | -2.22 | -7.91 | | 0.19 | 0.003196 | |
| -5.10 | phospholipid scramblase 4 | -2.09 | -8.03 | | 0.22 | 0.004390 | |
| -5.06 | adrenergic receptor kinase, beta 2 | -2.11 | -7.91 | | 0.21 | 0.003974 | |
| -5.04 | ankyrin repeat domain 10 | -2.15 | -7.84 | | 0.20 | 0.003553 | |
| -5.02 | TRAF type zinc finger domain containing 1 | -2.48 | -7.50 | | 0.15 | 0.001362 | |
| -5.02 | N-deacetylase/N-sulfotransferase (heparan glucosaminyl) 2 | -2.19 | -7.77 | | 0.19 | 0.003116 | |
| -4.98 | SCO cytochrome oxidase deficient homolog 1 (yeast) | -2.10 | -7.77 | | 0.20 | 0.003789 | |
| -4.97 | lipoic acid synthetase | -2.09 | -7.76 | | 0.20 | 0.003803 | |
| -4.94 | Sec24 related gene family, member B (S. cerevisiae) | -2.06 | -7.73 | | 0.21 | 0.003975 | |
| -4.85 | choline kinase beta | -1.96 | -7.64 | | 0.22 | 0.004596 | |
| -4.84 | DNA methyltransferase 3B | -2.16 | -7.45 | | 0.18 | 0.002706 | |
| -4.83 | Cobl-like 1 | -2.14 | -7.45 | | 0.19 | 0.002886 | |
| -4.81 | similar to Sideroflexin-2; sideroflexin 2 | -2.42 | -7.14 | | 0.14 | 0.001161 | |
| -4.69 | eukaryotic translation initiation factor 2C, 3 | -2.31 | -7.01 | | 0.15 | 0.001358 | |
| -4.63 | cytidine 5'-triphosphate synthase 2 | -1.93 | -7.25 | | 0.21 | 0.003966 | |
| -4.61 | formin homology 2 domain containing 1 | -1.91 | -7.24 | | 0.21 | 0.004129 | |
| -4.60 | rod outer segment membrane protein 1 | -1.84 | -7.28 | | 0.22 | 0.004802 | |
| -4.59 | homeodomain interacting protein kinase 1; similar to protein kinase Myak-S | -1.83 | -7.26 | | 0.23 | 0.004915 | |
| -4.54 | thrombospondin, type I, domain 1 | -1.95 | -7.06 | | 0.20 | 0.003406 | |
| -4.53 | SERTA domain containing 3 | -2.10 | -6.91 | | 0.17 | 0.002141 | |
| -4.53 | excision repair cross-complementing rodent repair deficiency, group 2 | -2.28 | -6.72 | | 0.14 | 0.001130 | |
| -4.48 | RIKEN cDNA 1110032A03 gene; hypothetical protein LOC100048251 | -2.29 | -6.62 | | 0.13 | 0.001020 | |
| -4.45 | MAP/microtubule affinity-regulating kinase 3 | -1.92 | -6.91 | | 0.20 | 0.003338 | |
| -4.39 | cytochrome P450, family 4, subfamily f, polypeptide 16 | -1.82 | -6.90 | | 0.21 | 0.004109 | |
| -4.32 | nuclear factor of activated T-cells, cytoplasmic, calcineurin-dependent 3 | -2.26 | -6.34 | | 0.13 | 0.000818 | |
| -4.32 | RIKEN cDNA E430029J22 gene; similar to Interferon-activatable protein 203 (Ifi-203) | -1.73 | -6.83 | | 0.22 | 0.004799 | |
| -4.24 | nitric oxide synthase 3, endothelial cell | -1.99 | -6.44 | | 0.17 | 0.001967 | |
| -4.09 | integrin beta 3 | -1.69 | -6.43 | | 0.21 | 0.004152 | |
| -4.06 | UDP-N-acetyl-alpha-D-galactosamine:polypeptide N-acetylgalactosaminyltransferase 1 | -1.82 | -6.26 | | 0.18 | 0.002628 | |
| -4.05 | NIMA (never in mitosis gene a)-related expressed kinase 6 | -2.24 | -5.82 | | 0.11 | 0.000452 | |
| -4.04 | PDX1 C-terminal inhibiting factor 1 | -1.99 | -6.04 | | 0.15 | 0.001337 | |
| -3.98 | UDP-GalNAc:betaGlcNAc beta 1,3-galactosaminyltransferase, polypeptide 2 | -2.17 | -5.76 | | 0.11 | 0.000531 | |
| -3.97 | ribosome binding protein 1 | -2.01 | -5.89 | | 0.14 | 0.001078 | |
| -3.96 | family with sequence similarity 82, member B | -1.68 | -6.19 | | 0.20 | 0.003555 | |
| -3.90 | transmembrane protein 201 | -1.70 | -6.04 | | 0.19 | 0.003082 | |
| -3.88 | protein disulfide isomerase associated 5 | -1.54 | -6.15 | | 0.23 | 0.004927 | |
| -3.87 | thymidylate synthase | -1.82 | -5.88 | | 0.17 | 0.001904 | |
| -3.81 | filamin, beta | -1.93 | -5.65 | | 0.14 | 0.001062 | |
| -3.75 | lymphoid enhancer binding factor 1 | -1.53 | -5.93 | | 0.22 | 0.004439 | |
| -3.74 | tumor necrosis factor, alpha-induced protein 3 | -1.81 | -5.64 | | 0.16 | 0.001577 | |
| -3.74 | transient receptor potential cation channel, subfamily C, member 1 | -1.67 | -5.77 | | 0.18 | 0.002722 | |
| -3.74 | meiotic nuclear divisions 1 homolog (S. cerevisiae) | -1.60 | -5.82 | | 0.20 | 0.003359 | |
| -3.69 | interleukin 1 family, member 10 | -1.93 | -5.41 | | 0.13 | 0.000779 | |
| -3.67 | clathrin, light polypeptide (Lca) | -1.72 | -5.59 | | 0.17 | 0.001965 | |
| -3.61 | cDNA sequence BC031353 | -2.11 | -5.09 | | 0.08 | 0.000222 | |
| -3.53 | cingulin-like 1 | -1.90 | -5.13 | | 0.12 | 0.000606 | |
| -3.52 | mab-21-like 2 (C. elegans) | -1.60 | -5.40 | | 0.18 | 0.002352 | |
| -3.52 | translocase of inner mitochondrial membrane 9 homolog (yeast) | -1.74 | -5.27 | | 0.15 | 0.001327 | |
| -3.51 | cathepsin 7 | -2.34 | -4.65 | | 0.04 | 0.000019 | |
| -3.49 | osteoglycin | -1.62 | -5.32 | | 0.17 | 0.002083 | |
| -3.47 | phospholipase A2, group IID | -1.62 | -5.29 | | 0.17 | 0.001991 | |
| -3.44 | smoothelin | -1.73 | -5.12 | | 0.14 | 0.001109 | |
| -3.37 | coiled-coil domain containing 39 | -1.73 | -4.98 | | 0.13 | 0.000932 | |
| -3.36 | toll-interleukin 1 receptor (TIR) domain-containing adaptor protein | -1.37 | -5.30 | | 0.22 | 0.004364 | |
| -3.25 | retinoic acid early transcript delta | -1.31 | -5.16 | | 0.22 | 0.004607 | |
| -3.25 | SWA-70 protein | -1.29 | -5.17 | | 0.23 | 0.004927 | |
| -3.17 | expressed sequence AI182371 | -1.72 | -4.60 | | 0.11 | 0.000554 | |
| -3.14 | predicted gene, EG665536; interferon induced transmembrane protein 7 | -1.27 | -4.98 | | 0.22 | 0.004478 | |
| -3.11 | signal recognition particle 54b; particle 54a; particle 54C | -1.82 | -4.39 | | 0.08 | 0.000214 | |
| -3.09 | DNA polymerase N | -1.74 | -4.43 | | 0.10 | 0.000366 | |
| -3.09 | beta-transducin repeat containing protein | -1.40 | -4.75 | | 0.18 | 0.002425 | |
| -3.08 | RIKEN cDNA D430041D05 gene | -1.28 | -4.85 | | 0.21 | 0.004008 | |
| -3.08 | mucosa associated lymphoid tissue lymphoma translocation gene 1 | -1.55 | -4.59 | | 0.14 | 0.001116 | |
| -3.07 | trafficking protein, kinesin binding 1 | -1.46 | -4.64 | | 0.16 | 0.001687 | |
| -3.06 | solute carrier family 6 (neurotransmitter transporter, GABA), member 1 | -1.31 | -4.77 | | 0.20 | 0.003334 | |
| -3.05 | nebulin-related anchoring protein | -1.23 | -4.83 | | 0.22 | 0.004568 | |
| -3.04 | predicted gene 8892; structural maintenace of chromosomes 3 | -1.32 | -4.73 | | 0.19 | 0.003163 | |
| -3.01 | olfactory receptor 1346 | -1.42 | -4.58 | | 0.17 | 0.001877 | |
| -2.98 | defensin beta 26 | -1.51 | -4.43 | | 0.14 | 0.001048 | |
| -2.95 | POU domain, class 1, transcription factor 1 (Pit1, growth hormone factor 1) | -1.32 | -4.55 | | 0.18 | 0.002601 | |
| -2.94 | vezatin, adherens junctions transmembrane protein | -1.25 | -4.60 | | 0.20 | 0.003504 | |
| -2.91 | activating transcription factor 2 | -1.18 | -4.61 | | 0.22 | 0.004441 | |
| -2.89 | prolactin family 2, subfamily b, member 1 | -1.43 | -4.33 | | 0.15 | 0.001292 | |
| -2.88 | neurofibromatosis 1 | -1.24 | -4.49 | | 0.19 | 0.003235 | |
| -2.84 | membrane-spanning 4-domains, subfamily A, member 4B | -1.36 | -4.30 | | 0.16 | 0.001686 | |
| -2.82 | predicted gene, EG665934 | -1.21 | -4.41 | | 0.20 | 0.003310 | |
| -2.75 | repetin | -1.26 | -4.22 | | 0.18 | 0.002286 | |
| -2.64 | predicted gene 5509; zinc finger, DHHC domain containing 6 | -1.33 | -3.92 | | 0.14 | 0.001064 | |
| -2.60 | eukaryotic translation initiation factor 3, subunit B | -1.13 | -4.05 | | 0.19 | 0.003039 | |
| -2.60 | carnitine palmitoyltransferase 1a, liver | -1.16 | -4.03 | | 0.18 | 0.002730 | |
| -2.60 | olfactory receptor 600 | -1.07 | -4.11 | | 0.21 | 0.004161 | |
| -2.60 | echinoderm microtubule associated protein like 1 | -1.09 | -4.08 | | 0.20 | 0.003717 | |
| -2.58 | engrailed 1 | -1.26 | -3.89 | | 0.15 | 0.001400 | |
| -2.57 | predicted gene 9027; predicted gene 9620; family with sequence similarity 60, member A | -1.12 | -4.00 | | 0.19 | 0.003001 | |
| -2.50 | BarH-like 2 (Drosophila) | -1.00 | -3.97 | | 0.22 | 0.004621 | |
| -2.49 | RIKEN cDNA 1810032O08 gene | -1.19 | -3.78 | | 0.16 | 0.001705 | |
| -2.49 | HEG homolog 1 (zebrafish) | -1.04 | -3.91 | | 0.20 | 0.003755 | |
| -2.36 | vaccinia related kinase 1 | -0.94 | -3.76 | | 0.22 | 0.004768 | |
| -2.06 | B-cell leukemia/lymphoma 2 related protein A1c | -0.91 | -3.19 | | 0.18 | 0.002676 | |
| -2.04 | RIKEN cDNA 9030624J02 gene | -0.93 | -3.14 | | 0.18 | 0.002294 | |
| -2.03 | cysteine-rich perinuclear theca 3 | -0.83 | -3.22 | | 0.21 | 0.004315 | |
| -1.98 | gene model 1505, (NCBI) | -0.88 | -3.06 | | 0.18 | 0.002644 | |
| -1.85 | predicted gene 364 | -0.76 | -2.93 | | 0.21 | 0.004130 | |
| -1.79 | dedicator of cytokinesis 6 | -0.88 | -2.68 | | 0.15 | 0.001264 | |
| -1.67 | hydroxy-delta-5-steroid dehydrogenase, 3 beta- and steroid delta-isomerase 2 | -0.68 | -2.64 | | 0.21 | 0.004119 | |
| -1.52 | olfactory receptor 1466 | -0.65 | -2.39 | | 0.20 | 0.003441 | |

| Exercise-induced changes in gene expression in the hippocampus  ***Upregulated*** | | **95% confidence intervals** | |  |  |
| --- | --- | --- | --- | --- | --- |
| **Percent change** | **Gene name** | **Lower** | **Upper** | **FDR** | **P value** |
| 48.60 | Wolfram syndrome 1 homolog (human) | 31.52 | 67.90 | 0.03 | 0.000007 |
| 44.71 | kallikrein related-peptidase 8 | 27.77 | 63.90 | 0.04 | 0.000021 |
| 38.54 | wingless-related MMTV integration site 2 | 25.74 | 52.64 | 0.03 | 0.000005 |
| 34.67 | predicted gene 2668 | 23.84 | 46.45 | 0.02 | 0.000002 |
| 34.45 | serine peptidase inhibitor, Kazal type 8 | 16.67 | 54.94 | 0.13 | 0.000761 |
| 31.24 | pleckstrin homology domain-containing, family A member 2 | 12.48 | 53.13 | 0.19 | 0.003026 |
| 31.04 | insulin-like growth factor binding protein 6 | 13.95 | 50.70 | 0.15 | 0.001456 |
| 30.44 | brain derived neurotrophic factor | 19.81 | 42.02 | 0.03 | 0.000011 |
| 28.17 | left right determination factor 1 | 13.48 | 44.76 | 0.13 | 0.000934 |
| 27.34 | doublecortin-like kinase 1 | 15.12 | 40.85 | 0.08 | 0.000207 |
| 27.32 | corticotropin releasing hormone binding protein | 14.52 | 41.56 | 0.10 | 0.000339 |
| 24.43 | RIKEN cDNA 2610017I09 gene | 12.34 | 37.82 | 0.12 | 0.000613 |
| 24.06 | neuroblastoma, suppression of tumorigenicity 1 | 15.30 | 33.48 | 0.04 | 0.000023 |
| 24.01 | abhydrolase domain containing 12 | 15.70 | 32.91 | 0.03 | 0.000012 |
| 23.93 | solute carrier family 38, member 5 | 10.30 | 39.24 | 0.17 | 0.002169 |
| 23.88 | family with sequence similarity 19, member A1 | 14.20 | 34.39 | 0.05 | 0.000079 |
| 23.08 | aryl hydrocarbon receptor nuclear translocator-like | 14.21 | 32.65 | 0.05 | 0.000044 |
| 21.63 | heat shock 105kDa/110kDa protein 1 | 15.07 | 28.56 | 0.02 | 0.000002 |
| 21.56 | polo-like kinase 2 (Drosophila) | 12.90 | 30.89 | 0.05 | 0.000076 |
| 21.36 | potassium voltage-gated channel, shaker-related subfamily, beta member 1 | 11.76 | 31.79 | 0.08 | 0.000253 |
| 21.26 | RAS protein-specific guanine nucleotide-releasing factor 1 | 11.47 | 31.92 | 0.10 | 0.000325 |
| 21.04 | VGF nerve growth factor inducible | 8.95 | 34.48 | 0.18 | 0.002435 |
| 20.88 | synaptic nuclear envelope 1 | 8.60 | 34.54 | 0.19 | 0.002922 |
| 20.74 | diacylglycerol kinase, beta | 9.01 | 33.73 | 0.17 | 0.002140 |
| 20.54 | double C2, beta | 9.40 | 32.81 | 0.16 | 0.001510 |
| 20.24 | G protein-coupled receptor 22 | 10.84 | 30.45 | 0.10 | 0.000362 |
| 20.23 | HECT domain containing 2 | 8.60 | 33.09 | 0.18 | 0.002460 |
| 19.72 | neuropeptide Y | 7.40 | 33.45 | 0.22 | 0.004722 |
| 19.29 | DNA segment, Chr 4, Brigham & Women's Genetics 0951 expressed | 8.69 | 30.93 | 0.16 | 0.001725 |
| 19.24 | tubulin, beta 2a, pseudogene 2; tubulin, beta 2B | 13.09 | 25.72 | 0.03 | 0.000005 |
| 19.12 | tescalcin; similar to Tescalcin | 11.58 | 27.17 | 0.05 | 0.000066 |
| 19.02 | ATPase, Ca++ transporting, plasma membrane 1 | 9.52 | 29.35 | 0.13 | 0.000741 |
| 18.89 | lecithin cholesterol acyltransferase | 9.84 | 28.68 | 0.11 | 0.000501 |
| 18.70 | RIKEN cDNA D430041B17 gene | 9.22 | 29.00 | 0.13 | 0.000858 |
| 18.24 | histone cluster 1, H2bf | 8.99 | 28.28 | 0.13 | 0.000865 |
| 18.23 | arginine vasopressin | 10.06 | 27.00 | 0.09 | 0.000263 |
| 17.93 | dystrobrevin alpha | 10.90 | 25.41 | 0.05 | 0.000064 |
| 17.90 | RAS guanyl releasing protein 1 | 6.89 | 30.05 | 0.21 | 0.004301 |
| 17.86 | histone cluster 1, H2bc | 8.14 | 28.45 | 0.16 | 0.001623 |
| 17.69 | histone cluster 1, H2bj | 10.23 | 25.66 | 0.07 | 0.000143 |
| 17.49 | chitinase domain containing 1 | 6.56 | 29.53 | 0.22 | 0.004845 |
| 17.44 | BCL2-related ovarian killer protein | 6.84 | 29.09 | 0.21 | 0.003964 |
| 17.15 | histone cluster 1, H2be | 10.33 | 24.40 | 0.05 | 0.000077 |
| 17.01 | predicted gene 7997; lactate dehydrogenase A; predicted gene 5452 | 6.79 | 28.21 | 0.20 | 0.003636 |
| 16.77 | cyclin D1 | 11.02 | 22.83 | 0.03 | 0.000014 |
| 16.64 | family with sequence similarity 148, member A | 6.53 | 27.71 | 0.21 | 0.003995 |
| 16.44 | inositol 1,4,5-trisphosphate 3-kinase A | 7.46 | 26.17 | 0.16 | 0.001707 |
| 16.32 | exocyst complex component 6B | 10.30 | 22.66 | 0.04 | 0.000034 |
| 16.13 | immunity-related GTPase family M member 1 | 8.08 | 24.77 | 0.13 | 0.000776 |
| 16.09 | diacylglycerol kinase zeta | 7.37 | 25.52 | 0.16 | 0.001607 |
| 15.97 | histone cluster 1, H2bh | 7.37 | 25.26 | 0.16 | 0.001537 |
| 15.88 | neuropeptide B | 9.20 | 22.96 | 0.07 | 0.000145 |
| 15.85 | NEL-like 2 (chicken) | 7.39 | 24.99 | 0.15 | 0.001425 |
| 15.58 | sortilin-related VPS10 domain containing receptor 3 | 9.41 | 22.09 | 0.05 | 0.000077 |
| 15.50 | monooxygenase, DBH-like 1 | 9.29 | 22.06 | 0.06 | 0.000088 |
| 15.42 | deleted in bladder cancer 1 (human) | 7.57 | 23.84 | 0.13 | 0.000943 |
| 15.32 | zinc finger, RAN-binding domain containing 3 | 10.47 | 20.38 | 0.03 | 0.000005 |
| 15.32 | solute carrier family 35, member F3 | 6.99 | 24.29 | 0.16 | 0.001679 |
| 15.18 | glutamate receptor, ionotropic, AMPA3 (alpha 3) | 7.83 | 23.04 | 0.12 | 0.000602 |
| 14.95 | sema domain, immunoglobulin domain, short basic domain, secreted, (semaphorin) 3E | 6.31 | 24.30 | 0.18 | 0.002770 |
| 14.95 | shadow of prion protein | 6.21 | 24.41 | 0.19 | 0.003033 |
| 14.82 | potassium channel, subfamily K, member 1 | 8.48 | 21.54 | 0.07 | 0.000180 |
| 14.81 | histone cluster 1, H2bn | 6.37 | 23.91 | 0.18 | 0.002481 |
| 14.67 | copine VIII | 6.12 | 23.92 | 0.19 | 0.002978 |
| 14.58 | transmembrane protein 68 | 6.26 | 23.55 | 0.18 | 0.002522 |
| 14.49 | ets variant gene 5 | 6.19 | 23.45 | 0.18 | 0.002617 |
| 14.45 | similar to Interferon-activatable protein 204 (Ifi-204) | 9.03 | 20.14 | 0.05 | 0.000043 |
| 14.42 | RIKEN cDNA 6530418L21 gene | 6.58 | 22.84 | 0.16 | 0.001702 |
| 14.38 | nucleolar protein 4 | 6.83 | 22.47 | 0.15 | 0.001269 |
| 14.17 | cDNA sequence BC024659 | 7.15 | 21.64 | 0.13 | 0.000754 |
| 14.16 | predicted gene 8990; neuroepithelial cell transforming gene 1 | 5.87 | 23.11 | 0.19 | 0.003118 |
| 14.06 | histone cluster 1, H2bm | 6.34 | 22.33 | 0.17 | 0.001850 |
| 14.04 | cytoplasmic dynein light chain 1 | 8.86 | 19.47 | 0.05 | 0.000036 |
| 14.02 | histone cluster 1, H4a | 5.44 | 23.29 | 0.22 | 0.004350 |
| 13.99 | protein phosphatase 4, regulatory subunit 4 | 5.29 | 23.42 | 0.23 | 0.004899 |
| 13.97 | histone cluster 1, H2bk | 7.10 | 21.28 | 0.12 | 0.000710 |
| 13.84 | SH3 domain binding glutamic acid-rich protein-like 3 | 6.94 | 21.19 | 0.13 | 0.000813 |
| 13.47 | subacute ozone induced inflammation; RIKEN cDNA 2610204M08 gene | 5.81 | 21.68 | 0.18 | 0.002485 |
| 13.44 | cysteine dioxygenase 1, cytosolic | 5.43 | 22.07 | 0.20 | 0.003609 |
| 13.35 | calcium/calmodulin-dependent protein kinase kinase 2, beta | 5.74 | 21.49 | 0.18 | 0.002538 |
| 13.33 | McKusick-Kaufman syndrome protein | 7.04 | 19.98 | 0.11 | 0.000489 |
| 13.31 | expressed sequence AI593442 | 6.62 | 20.42 | 0.13 | 0.000884 |
| 13.25 | neurofilament, medium polypeptide | 5.36 | 21.73 | 0.20 | 0.003578 |
| 13.05 | predicted gene 705 | 6.42 | 20.10 | 0.13 | 0.000974 |
| 12.92 | solute carrier family 8 (sodium/calcium exchanger), member 1 | 7.40 | 18.74 | 0.07 | 0.000187 |
| 12.80 | RIKEN cDNA 4933433P14 gene | 9.07 | 16.66 | 0.02 | 0.000002 |
| 12.78 | RIMS binding protein 2 | 8.31 | 17.43 | 0.04 | 0.000020 |
| 12.75 | RIKEN cDNA 1810046J19 gene | 6.04 | 19.87 | 0.15 | 0.001321 |
| 12.66 | histone cluster 1, H4f | 6.91 | 18.72 | 0.10 | 0.000346 |
| 12.65 | mediator complex subunit 23 | 5.33 | 20.49 | 0.19 | 0.002914 |
| 12.65 | four and a half LIM domains 2 | 6.25 | 19.43 | 0.13 | 0.000945 |
| 12.64 | myeloma overexpressed 2 | 7.36 | 18.17 | 0.07 | 0.000148 |
| 12.49 | endosulfine alpha isoform 1 | 5.28 | 20.20 | 0.19 | 0.002865 |
| 12.48 | kelch repeat and BTB (POZ) domain containing 5 | 7.17 | 18.05 | 0.07 | 0.000178 |
| 12.41 | lymphocyte antigen 6 complex, locus G6E | 6.75 | 18.37 | 0.10 | 0.000362 |
| 12.35 | solute carrier family 1 (glial high affinity glutamate transporter), member 2 | 4.82 | 20.43 | 0.22 | 0.004328 |
| 12.16 | solute carrier family 23 (nucleobase transporters), member 3 | 6.86 | 17.72 | 0.08 | 0.000230 |
| 12.06 | FK506 binding protein 1a | 4.82 | 19.80 | 0.21 | 0.003881 |
| 12.03 | fucosyltransferase 8 | 6.81 | 17.51 | 0.08 | 0.000220 |
| 11.98 | poly (ADP-ribose) polymerase family, member 1 | 6.90 | 17.30 | 0.07 | 0.000174 |
| 11.89 | tripartite motif-containing 2 | 5.26 | 18.94 | 0.18 | 0.002180 |
| 11.83 | protein kinase C, beta | 4.80 | 19.33 | 0.20 | 0.003604 |
| 11.77 | microtubule-associated protein 4 | 6.02 | 17.84 | 0.12 | 0.000700 |
| 11.75 | melanocortin 4 receptor | 4.82 | 19.14 | 0.20 | 0.003394 |
| 11.68 | synaptotagmin XII | 6.64 | 16.96 | 0.08 | 0.000212 |
| 11.67 | OCIA domain containing 2 | 5.15 | 18.60 | 0.18 | 0.002237 |
| 11.67 | ubiquitin-conjugating enzyme E2A, RAD6 homolog (S. cerevisiae) | 7.04 | 16.49 | 0.06 | 0.000087 |
| 11.65 | zinc finger, DHHC domain containing 14 | 4.63 | 19.14 | 0.21 | 0.003997 |
| 11.65 | docking protein 3 | 5.85 | 17.76 | 0.13 | 0.000832 |
| 11.63 | lactation elevated 1 | 5.56 | 18.04 | 0.15 | 0.001263 |
| 11.60 | neurexin III | 5.76 | 17.77 | 0.13 | 0.000921 |
| 11.55 | cysteine and histidine-rich domain (CHORD)-containing, zinc-binding protein 1 | 6.22 | 17.14 | 0.10 | 0.000414 |
| 11.49 | cDNA sequence BC002230 | 5.02 | 18.35 | 0.18 | 0.002387 |
| 11.47 | tyrosine 3-monooxygenase/tryptophan 5-monooxygenase activation protein | 4.95 | 18.39 | 0.18 | 0.002574 |
| 11.43 | dual specificity phosphatase 19 | 6.32 | 16.79 | 0.09 | 0.000303 |
| 11.42 | UDP-N-acetyl-alpha-D-galactosamine:polypeptide N-acetylgalactosaminyltransferase-like 4 | 5.44 | 17.74 | 0.15 | 0.001309 |
| 11.38 | transmembrane protein 158 | 6.43 | 16.55 | 0.08 | 0.000228 |
| 11.38 | RIKEN cDNA 2210408F21 gene | 4.98 | 18.16 | 0.18 | 0.002355 |
| 11.36 | zeta-chain (TCR) associated protein kinase | 5.70 | 17.31 | 0.13 | 0.000838 |
| 11.35 | IQ motif and Sec7 domain 2 | 5.77 | 17.22 | 0.13 | 0.000751 |
| 11.34 | lysophospholipase 2 | 6.85 | 16.01 | 0.06 | 0.000085 |
| 11.32 | protein disulfide isomerase associated 6 | 7.07 | 15.75 | 0.05 | 0.000049 |
| 11.28 | general transcription factor IIIC, polypeptide 2, beta; transgene, kidney disease mutant | 5.78 | 17.06 | 0.12 | 0.000692 |
| 11.19 | membrane-associated ring finger (C3HC4) 4 | 4.77 | 18.01 | 0.18 | 0.002763 |
| 11.14 | predicted gene 11868 | 5.68 | 16.87 | 0.13 | 0.000725 |
| 11.13 | predicted gene 13102; preferentially expressed antigen in melanoma like 4 | 7.09 | 15.33 | 0.04 | 0.000033 |
| 11.11 | protein arginine N-methyltransferase 8 | 5.15 | 17.41 | 0.16 | 0.001618 |
| 11.04 | nudix (nucleoside diphosphate linked moiety X)-type motif 5 | 5.02 | 17.41 | 0.17 | 0.001860 |
| 11.01 | RIKEN cDNA 4930429B21 gene | 6.04 | 16.21 | 0.10 | 0.000337 |
| 11.00 | predicted gene 5064; cold shock domain containing E1, RNA binding | 5.51 | 16.78 | 0.13 | 0.000872 |
| 10.99 | U6 small nuclear RNA | 5.18 | 17.12 | 0.15 | 0.001435 |
| 10.97 | cytoplasmic dynein light chain 1 | 6.03 | 16.13 | 0.10 | 0.000329 |
| 10.90 | RIKEN cDNA 1810064F22 gene | 5.03 | 17.09 | 0.16 | 0.001662 |
| 10.89 | kyphoscoliosis peptidase | 5.05 | 17.05 | 0.16 | 0.001603 |
| 10.69 | Eph receptor A6 | 5.63 | 15.99 | 0.11 | 0.000538 |
| 10.62 | ELOVL family member 6, elongation of long chain fatty acids (yeast) | 5.25 | 16.25 | 0.13 | 0.000972 |
| 10.57 | glutamate receptor, ionotropic, NMDA1 (zeta 1) | 4.74 | 16.72 | 0.17 | 0.002047 |
| 10.56 | factor inhibiting activating transcription factor 4 (ATF4)-mediated transcription | 6.57 | 14.70 | 0.05 | 0.000053 |
| 10.48 | RAB8B, member RAS oncogene family | 5.29 | 15.93 | 0.13 | 0.000811 |
| 10.48 | mitochondrial ribosomal protein L36 | 5.07 | 16.17 | 0.14 | 0.001176 |
| 10.47 | RIKEN cDNA 9430023L20 gene | 3.99 | 17.37 | 0.23 | 0.004949 |
| 10.44 | ribosomal RNA processing 12 homolog (S. cerevisiae) | 4.96 | 16.21 | 0.15 | 0.001361 |
| 10.44 | sestrin 1 | 4.44 | 16.79 | 0.19 | 0.002853 |
| 10.42 | RIKEN cDNA 1110057K04 gene | 6.68 | 14.29 | 0.04 | 0.000030 |
| 10.41 | cytochrome b5 reductase 1 | 5.43 | 15.63 | 0.12 | 0.000598 |
| 10.38 | ubiquitin carboxyl-terminal esterase L3 (ubiquitin thiolesterase) | 4.59 | 16.48 | 0.18 | 0.002235 |
| 10.33 | Fc receptor-like S, scavenger receptor | 4.65 | 16.32 | 0.17 | 0.002002 |
| 10.33 | protein-L-isoaspartate (D-aspartate) O-methyltransferase domain containing 1 | 6.63 | 14.15 | 0.04 | 0.000029 |
| 10.29 | cadherin 10 | 5.92 | 14.85 | 0.07 | 0.000187 |
| 10.29 | otospiralin | 4.33 | 16.59 | 0.19 | 0.003008 |
| 10.24 | esterase D/formylglutathione hydrolase | 6.25 | 14.39 | 0.05 | 0.000076 |
| 10.24 | mediator complex subunit 14 | 6.85 | 13.74 | 0.03 | 0.000011 |
| 10.19 | similar to sortilin-related receptor, LDLR class A repeats-containing | 4.41 | 16.29 | 0.18 | 0.002572 |
| 10.16 | tribbles homolog 2 (Drosophila) | 4.81 | 15.78 | 0.15 | 0.001397 |
| 10.14 | RIKEN cDNA 4933403G14 gene | 5.58 | 14.89 | 0.10 | 0.000332 |
| 10.13 | syntaxin 6 | 4.20 | 16.40 | 0.20 | 0.003271 |
| 10.03 | neuropeptide Y receptor Y1 | 4.16 | 16.23 | 0.20 | 0.003298 |
| 9.99 | mitogen-activated protein kinase kinase kinase kinase 3 | 5.05 | 15.17 | 0.13 | 0.000818 |
| 9.98 | neuropeptide Y receptor Y5 | 4.37 | 15.90 | 0.18 | 0.002411 |
| 9.98 | RAS, dexamethasone-induced 1 | 4.60 | 15.64 | 0.16 | 0.001712 |
| 9.98 | ecto-NOX disulfide-thiol exchanger 1 | 4.76 | 15.45 | 0.15 | 0.001331 |
| 9.88 | transmembrane protein 183A | 6.65 | 13.20 | 0.03 | 0.000010 |
| 9.86 | transmembrane protein 70 | 5.85 | 14.03 | 0.07 | 0.000121 |
| 9.82 | proprotein convertase subtilisin/kexin type 2 | 4.68 | 15.22 | 0.15 | 0.001345 |
| 9.76 | doublecortin-like kinase 1 | 3.75 | 16.12 | 0.22 | 0.004819 |
| 9.69 | cell adhesion molecule 2 | 5.12 | 14.45 | 0.11 | 0.000525 |
| 9.63 | ARP3 actin-related protein 3 homolog (yeast) | 4.27 | 15.26 | 0.18 | 0.002227 |
| 9.52 | protein phosphatase 1E (PP2C domain containing) | 3.78 | 15.57 | 0.21 | 0.004107 |
| 9.44 | phosphatidic acid phosphatase type 2 domain containing 2 | 3.79 | 15.41 | 0.21 | 0.003932 |
| 9.43 | EPM2A (laforin) interacting protein 1 | 6.34 | 12.62 | 0.03 | 0.000011 |
| 9.43 | abhydrolase domain containing 1 | 3.68 | 15.49 | 0.22 | 0.004456 |
| 9.42 | YdjC homolog (bacterial) | 4.66 | 14.39 | 0.13 | 0.000997 |
| 9.38 | PRP31 pre-mRNA processing factor 31 homolog (yeast) | 5.08 | 13.85 | 0.10 | 0.000404 |
| 9.37 | DIP2 disco-interacting protein 2 homolog C (Drosophila) | 4.14 | 14.87 | 0.18 | 0.002289 |
| 9.35 | cysteine-serine-rich nuclear protein 3 | 4.91 | 13.99 | 0.11 | 0.000573 |
| 9.27 | sorting nexin 25 | 5.72 | 12.93 | 0.05 | 0.000063 |
| 9.25 | RIKEN cDNA 5730507A09 gene | 4.05 | 14.71 | 0.18 | 0.002420 |
| 9.17 | procollagen-proline, 2-oxoglutarate 4-dioxygenase , alpha 1 polypeptide | 5.68 | 12.77 | 0.05 | 0.000059 |
| 9.17 | RuvB-like protein 1 | 3.93 | 14.67 | 0.18 | 0.002775 |
| 9.17 | sno, strawberry notch homolog 1 (Drosophila) | 4.77 | 13.74 | 0.12 | 0.000620 |
| 9.13 | vacuolar protein sorting 29 (S. pombe) | 3.54 | 15.03 | 0.22 | 0.004670 |
| 9.12 | Rho guanine nucleotide exchange factor (GEF7) | 5.47 | 12.90 | 0.06 | 0.000105 |
| 9.11 | epidermal growth factor receptor pathway substrate 15 | 4.57 | 13.85 | 0.13 | 0.000892 |
| 9.08 | sarcalumenin | 4.10 | 14.30 | 0.17 | 0.001993 |
| 9.06 | transmembrane protein 50B | 3.99 | 14.37 | 0.18 | 0.002341 |
| 9.03 | discs, large (Drosophila) homolog-associated protein 1 | 4.12 | 14.16 | 0.17 | 0.001856 |
| 9.03 | methyltransferase like 3 | 5.02 | 13.19 | 0.09 | 0.000302 |
| 9.02 | gephyrin | 5.30 | 12.88 | 0.07 | 0.000143 |
| 8.97 | histone cluster 1, H4i | 3.62 | 14.59 | 0.21 | 0.003837 |
| 8.94 | syntaxin 12 | 3.68 | 14.46 | 0.20 | 0.003469 |
| 8.94 | mannan-binding lectin serine peptidase 2 | 3.96 | 14.15 | 0.18 | 0.002256 |
| 8.89 | solute carrier family 35, member C2 | 4.03 | 13.98 | 0.17 | 0.001956 |
| 8.87 | Ras-related GTP binding D | 4.24 | 13.72 | 0.15 | 0.001348 |
| 8.73 | archaelysin family metallopeptidase 2 | 4.09 | 13.57 | 0.16 | 0.001545 |
| 8.71 | vitamin K epoxide reductase complex, subunit 1-like 1 | 5.10 | 12.44 | 0.07 | 0.000152 |
| 8.70 | ubiquitin-conjugating enzyme E2B, RAD6 homology (S. cerevisiae) | 4.26 | 13.33 | 0.14 | 0.001101 |
| 8.69 | leucine rich repeat containing 7 | 4.95 | 12.56 | 0.08 | 0.000221 |
| 8.66 | Notch gene homolog 4 (Drosophila) | 4.12 | 13.38 | 0.15 | 0.001371 |
| 8.64 | predicted gene 5617 | 4.34 | 13.12 | 0.13 | 0.000892 |
| 8.58 | RIKEN cDNA 2210021J22 gene | 3.82 | 13.56 | 0.18 | 0.002219 |
| 8.56 | tumor suppressor candidate 2 | 3.77 | 13.58 | 0.18 | 0.002394 |
| 8.51 | chemokine (C-C motif) ligand 25 | 4.07 | 13.14 | 0.15 | 0.001340 |
| 8.48 | adducin 1 (alpha) | 4.29 | 12.85 | 0.13 | 0.000841 |
| 8.46 | CCR4-NOT transcription complex, subunit 4 | 3.42 | 13.74 | 0.20 | 0.003823 |
| 8.44 | hypothetical protein LOC100045501 | 5.50 | 11.46 | 0.04 | 0.000022 |
| 8.41 | TAF10 RNA polymerase II, TATA box binding protein (TBP)-associated factor | 3.61 | 13.43 | 0.18 | 0.002771 |
| 8.41 | epoxide hydrolase 2, cytoplasmic | 4.44 | 12.52 | 0.11 | 0.000542 |
| 8.40 | eukaryotic translation elongation factor 1 delta (guanine nucleotide exchange protein) | 4.94 | 11.97 | 0.07 | 0.000141 |
| 8.37 | ras homolog gene family, member T1 | 3.93 | 12.99 | 0.16 | 0.001529 |
| 8.34 | CAAX box 1 homolog C (human) | 3.36 | 13.55 | 0.21 | 0.003889 |
| 8.32 | CSA-conditional, T cell activation-dependent protein | 3.65 | 13.21 | 0.18 | 0.002450 |
| 8.23 | RIKEN cDNA 4930455C21 gene | 3.83 | 12.81 | 0.16 | 0.001641 |
| 8.19 | mitochondrial ribosomal protein 63 | 5.19 | 11.27 | 0.05 | 0.000040 |
| 8.15 | HIV-1 tat interactive protein 2, homolog (human) | 4.19 | 12.25 | 0.12 | 0.000712 |
| 8.14 | predicted gene 6123; pyruvate dehydrogenase (lipoamide) beta | 3.16 | 13.35 | 0.22 | 0.004653 |
| 8.12 | taspase, threonine aspartase 1 | 4.54 | 11.83 | 0.09 | 0.000288 |
| 8.09 | RIKEN cDNA 2210008F06 gene | 3.74 | 12.62 | 0.16 | 0.001721 |
| 8.05 | dehydrogenase/reductase (SDR family) member 7B | 4.72 | 11.48 | 0.07 | 0.000152 |
| 8.03 | armadillo repeat containing, X-linked 3 | 3.24 | 13.05 | 0.21 | 0.003917 |
| 8.03 | autophagy-related 12 (yeast) | 4.09 | 12.12 | 0.13 | 0.000795 |
| 8.01 | isoamyl acetate-hydrolyzing esterase 1 homolog (S. cerevisiae) | 3.47 | 12.75 | 0.18 | 0.002626 |
| 8.01 | predicted gene 9731; RIKEN cDNA 1810009O10 gene | 3.48 | 12.73 | 0.18 | 0.002589 |
| 7.90 | zinc finger protein 804A | 3.04 | 12.99 | 0.23 | 0.004909 |
| 7.89 | karyopherin (importin) alpha 1 | 4.91 | 10.95 | 0.05 | 0.000057 |
| 7.86 | guanine nucleotide binding protein (G protein), beta 5 | 3.48 | 12.43 | 0.18 | 0.002340 |
| 7.85 | COP9 (constitutive photomorphogenic) homolog, subunit 8 (Arabidopsis thaliana) | 3.63 | 12.24 | 0.16 | 0.001741 |
| 7.83 | zinc finger protein 277 | 3.02 | 12.86 | 0.22 | 0.004837 |
| 7.80 | dynactin 5 | 4.43 | 11.28 | 0.08 | 0.000238 |
| 7.79 | olfactomedin 4 | 4.23 | 11.47 | 0.10 | 0.000413 |
| 7.76 | inositol polyphosphate-5-phosphatase F | 3.66 | 12.02 | 0.16 | 0.001516 |
| 7.73 | cyclin C | 4.25 | 11.32 | 0.10 | 0.000355 |
| 7.72 | RIKEN cDNA 2210016F16 gene | 4.55 | 10.99 | 0.07 | 0.000141 |
| 7.71 | oligodendrocyte myelin glycoprotein | 5.46 | 10.02 | 0.02 | 0.000003 |
| 7.71 | kallikrein related-peptidase 7 (chymotryptic, stratum corneum) | 3.51 | 12.08 | 0.17 | 0.001928 |
| 7.67 | RIKEN cDNA 1110008F13 gene | 3.13 | 12.42 | 0.20 | 0.003686 |
| 7.67 | RIKEN cDNA 2310033P09 gene | 3.90 | 11.58 | 0.13 | 0.000811 |
| 7.64 | uridine-cytidine kinase 2 | 3.46 | 11.99 | 0.17 | 0.002021 |
| 7.64 | tetratricopeptide repeat domain 30B | 3.56 | 11.88 | 0.16 | 0.001658 |
| 7.62 | claspin homolog (Xenopus laevis) | 3.64 | 11.76 | 0.15 | 0.001373 |
| 7.61 | cytochrome b5 outer mitochondrial membrane precursor; cytochrome b5 type B | 3.57 | 11.80 | 0.16 | 0.001571 |
| 7.60 | testis specific gene A14 | 4.63 | 10.66 | 0.06 | 0.000083 |
| 7.60 | inositol polyphosphate-4-phosphatase, type I | 4.53 | 10.77 | 0.07 | 0.000120 |
| 7.58 | ADP-ribosylation factor-like 8B | 3.35 | 11.99 | 0.18 | 0.002371 |
| 7.57 | tudor and KH domain containing protein | 4.00 | 11.26 | 0.11 | 0.000554 |
| 7.51 | RIKEN cDNA 6030419C18 gene; hypothetical protein LOC100048292 | 3.51 | 11.67 | 0.16 | 0.001625 |
| 7.50 | phosphofructokinase, platelet | 3.27 | 11.91 | 0.18 | 0.002572 |
| 7.47 | gamma-aminobutyric acid (GABA) A receptor, subunit gamma 2 | 4.05 | 11.00 | 0.10 | 0.000418 |
| 7.45 | component of oligomeric golgi complex 4 | 3.78 | 11.26 | 0.13 | 0.000834 |
| 7.45 | similar to DnaJ (Hsp40) homolog, subfamily A, member 4 | 3.26 | 11.81 | 0.18 | 0.002516 |
| 7.43 | poly (ADP-ribose) polymerase family, member 6 | 3.22 | 11.82 | 0.18 | 0.002680 |
| 7.43 | transmembrane serine protease 6 | 4.13 | 10.83 | 0.10 | 0.000312 |
| 7.42 | cDNA sequence BC003965 | 2.92 | 12.12 | 0.22 | 0.004424 |
| 7.42 | ring finger protein 24 | 3.13 | 11.88 | 0.19 | 0.003098 |
| 7.41 | RIKEN cDNA 3110070M22 gene | 3.95 | 10.97 | 0.11 | 0.000501 |
| 7.34 | protein-L-isoaspartate (D-aspartate) O-methyltransferase domain containing 1 | 3.72 | 11.10 | 0.13 | 0.000845 |
| 7.32 | RIKEN cDNA 6720456B07 gene | 3.61 | 11.17 | 0.14 | 0.001074 |
| 7.32 | Bardet-Biedl syndrome 10 (human) | 4.38 | 10.35 | 0.06 | 0.000114 |
| 7.32 | REX2, RNA exonuclease 2 homolog (S. cerevisiae) | 4.18 | 10.55 | 0.08 | 0.000222 |
| 7.28 | solute carrier family 25 (mitochondrial carrier, brain), member 14 | 3.34 | 11.37 | 0.17 | 0.001842 |
| 7.28 | similar to Serine/threonine-protein kinase QSK; cDNA sequence BC033915 | 3.02 | 11.71 | 0.20 | 0.003420 |
| 7.23 | THAP domain containing 7 | 3.39 | 11.20 | 0.16 | 0.001573 |
| 7.22 | malectin | 4.01 | 10.53 | 0.10 | 0.000322 |
| 7.16 | similar to Rab6 protein; predicted gene 13082; RAB6, member RAS oncogene family | 3.37 | 11.09 | 0.16 | 0.001553 |
| 7.15 | similar to carbonic reductase 4 | 2.93 | 11.55 | 0.20 | 0.003649 |
| 7.12 | tocopherol (alpha) transfer protein-like | 4.44 | 9.86 | 0.05 | 0.000055 |
| 7.11 | histone cluster 1, H3e | 3.09 | 11.29 | 0.18 | 0.002625 |
| 7.10 | ubiquitin-conjugating enzyme E2N | 3.06 | 11.29 | 0.18 | 0.002739 |
| 7.08 | DEAH (Asp-Glu-Ala-His) box polypeptide 8 | 4.31 | 9.93 | 0.06 | 0.000088 |
| 7.07 | origin recognition complex, subunit 4-like (S. cerevisiae) | 4.16 | 10.07 | 0.07 | 0.000146 |
| 7.06 | leukocyte receptor cluster (LRC) member 9 | 3.82 | 10.41 | 0.11 | 0.000438 |
| 7.06 | TMEM9 domain family, member B | 3.22 | 11.04 | 0.17 | 0.001936 |
| 7.02 | WD repeat and SOCS box-containing 2 | 2.76 | 11.47 | 0.22 | 0.004511 |
| 7.00 | RIKEN cDNA 1700052N19 gene | 3.01 | 11.15 | 0.19 | 0.002836 |
| 6.99 | RIKEN cDNA B830017H08 gene | 3.06 | 11.06 | 0.18 | 0.002506 |
| 6.98 | 5-azacytidine induced gene 2 | 3.29 | 10.81 | 0.16 | 0.001550 |
| 6.98 | fer (fms/fps related) protein kinase, testis specific 2 | 3.53 | 10.55 | 0.13 | 0.000869 |
| 6.93 | SEC63-like (S. cerevisiae) | 3.80 | 10.16 | 0.10 | 0.000380 |
| 6.92 | shisa homolog 5 (Xenopus laevis) | 2.92 | 11.09 | 0.19 | 0.003158 |
| 6.92 | RIKEN cDNA 1700019N12 gene | 3.48 | 10.47 | 0.13 | 0.000904 |
| 6.90 | UDP-N-acetyl-alpha-D-galactosamine:polypeptide N-acetylgalactosaminyltransferase 9 | 2.89 | 11.08 | 0.20 | 0.003301 |
| 6.90 | IMP2 inner mitochondrial membrane peptidase-like (S. cerevisiae) | 3.16 | 10.78 | 0.17 | 0.001874 |
| 6.88 | glucosidase, beta, acid | 3.00 | 10.92 | 0.18 | 0.002622 |
| 6.88 | RIKEN cDNA 1700021K02 gene | 2.84 | 11.07 | 0.20 | 0.003500 |
| 6.86 | retinol dehydrogenase 14 (all-trans and 9-cis) | 3.76 | 10.06 | 0.10 | 0.000379 |
| 6.86 | expressed sequence AU022252 | 2.81 | 11.07 | 0.20 | 0.003683 |
| 6.85 | signal peptidase complex subunit 3 homolog (S. cerevisiae) | 3.48 | 10.33 | 0.13 | 0.000836 |
| 6.84 | signal recognition particle receptor ('docking protein') | 3.01 | 10.82 | 0.18 | 0.002450 |
| 6.79 | latrophilin 3 | 3.53 | 10.16 | 0.12 | 0.000670 |
| 6.77 | proline synthetase co-transcribed | 2.95 | 10.73 | 0.18 | 0.002611 |
| 6.73 | actin related protein 2/3 complex, subunit 5-like | 2.71 | 10.91 | 0.21 | 0.004007 |
| 6.72 | adaptor-related protein complex 2, beta 1 subunit | 3.01 | 10.56 | 0.18 | 0.002184 |
| 6.66 | phosphodiesterase 4D interacting protein (myomegalin) | 2.99 | 10.45 | 0.17 | 0.002148 |
| 6.64 | selenocysteine lyase | 3.05 | 10.36 | 0.17 | 0.001872 |
| 6.63 | crystallin, lambda 1 | 3.08 | 10.30 | 0.16 | 0.001720 |
| 6.62 | RIKEN cDNA 2810004N23 gene | 2.61 | 10.80 | 0.22 | 0.004461 |
| 6.59 | claudin 10 | 2.87 | 10.43 | 0.18 | 0.002593 |
| 6.53 | unc-80 homolog (C. elegans) | 2.93 | 10.27 | 0.18 | 0.002209 |
| 6.52 | stannin | 3.16 | 9.98 | 0.15 | 0.001232 |
| 6.50 | similar to Manbal protein; mannosidase, beta A, lysosomal-like | 3.78 | 9.29 | 0.07 | 0.000177 |
| 6.44 | UDP-GlcNAc:betaGal beta-1,3-N-acetylglucosaminyltransferase 9 | 3.40 | 9.58 | 0.11 | 0.000579 |
| 6.42 | zinc finger protein 580 | 2.51 | 10.48 | 0.22 | 0.004637 |
| 6.41 | KN motif and ankyrin repeat domains 3 | 2.81 | 10.13 | 0.18 | 0.002515 |
| 6.37 | rho/rac guanine nucleotide exchange factor (GEF) 2 | 3.10 | 9.74 | 0.15 | 0.001203 |
| 6.36 | mitochondrial ribosomal protein S25 | 3.05 | 9.78 | 0.15 | 0.001363 |
| 6.36 | aldehyde dehydrogenase 3 family, member B1 | 3.20 | 9.62 | 0.13 | 0.000909 |
| 6.36 | potassium inwardly-rectifying channel, subfamily J, member 3 | 2.63 | 10.22 | 0.20 | 0.003513 |
| 6.31 | metallo-beta-lactamase domain containing 1 | 2.98 | 9.75 | 0.16 | 0.001531 |
| 6.30 | cAMP responsive element binding protein 1 | 3.34 | 9.35 | 0.11 | 0.000558 |
| 6.30 | BCL2-associated athanogene 4 | 3.49 | 9.17 | 0.10 | 0.000330 |
| 6.29 | similar to Hbs1l protein; predicted gene 9923; Hbs1-like (S. cerevisiae) | 3.02 | 9.67 | 0.15 | 0.001354 |
| 6.29 | DNA segment, Chr 6, Wayne State University 163, expressed | 3.29 | 9.38 | 0.12 | 0.000627 |
| 6.27 | CLPTM1-like | 3.00 | 9.66 | 0.15 | 0.001411 |
| 6.27 | catenin, beta like 1 | 3.02 | 9.63 | 0.15 | 0.001334 |
| 6.27 | dystrophia myotonica-containing WD repeat motif | 2.44 | 10.23 | 0.22 | 0.004690 |
| 6.26 | COX19 cytochrome c oxidase assembly homolog (S. cerevisiae) | 3.04 | 9.58 | 0.15 | 0.001229 |
| 6.22 | ubiquitin specific peptidase 33 | 3.06 | 9.48 | 0.14 | 0.001104 |
| 6.22 | family with sequence similarity 173, member B | 2.73 | 9.82 | 0.18 | 0.002496 |
| 6.20 | fer (fms/fps related) protein kinase, testis specific 2 | 2.47 | 10.06 | 0.21 | 0.004258 |
| 6.19 | LTV1 homolog (S. cerevisiae) | 3.12 | 9.34 | 0.13 | 0.000884 |
| 6.18 | similar to cyclin N-terminal domain containing 1; cyclin N-terminal domain containing 1 | 2.42 | 10.08 | 0.22 | 0.004638 |
| 6.18 | F-box and WD-40 domain protein 2 | 2.60 | 9.87 | 0.19 | 0.003199 |
| 6.17 | mitochondrial ribosomal protein S16; predicted gene 9173 | 2.93 | 9.52 | 0.16 | 0.001491 |
| 6.16 | glutathione S-transferase, mu 4 | 2.53 | 9.93 | 0.20 | 0.003675 |
| 6.16 | akirin 1 | 3.31 | 9.10 | 0.11 | 0.000481 |
| 6.15 | misshapen-like kinase 1 (zebrafish) | 3.05 | 9.35 | 0.14 | 0.001036 |
| 6.10 | histone cluster 1, H4k | 2.70 | 9.62 | 0.18 | 0.002413 |
| 6.06 | structural maintenance of chromosomes 5 | 2.83 | 9.39 | 0.16 | 0.001672 |
| 6.03 | similar to CG7338-PA; TSR1, 20S rRNA accumulation, homolog (yeast) | 2.38 | 9.82 | 0.22 | 0.004499 |
| 6.02 | carbonic anhydrase 15 | 2.81 | 9.33 | 0.16 | 0.001690 |
| 6.02 | transforming growth factor beta regulated gene 1 | 3.49 | 8.61 | 0.07 | 0.000186 |
| 6.01 | expressed sequence AU014645; nuclear cap binding protein subunit 1 | 2.91 | 9.19 | 0.15 | 0.001269 |
| 6.00 | calpain 2 | 2.85 | 9.25 | 0.16 | 0.001490 |
| 6.00 | suppressor of cytokine signaling 5 | 2.76 | 9.33 | 0.17 | 0.001846 |
| 5.96 | similar to CDNA sequence AK129341; cDNA sequence AK129341 | 3.11 | 8.90 | 0.12 | 0.000657 |
| 5.94 | small nuclear ribonucleoprotein polypeptide A | 2.43 | 9.57 | 0.20 | 0.003738 |
| 5.90 | histone cluster 1, H4m | 2.58 | 9.33 | 0.18 | 0.002573 |
| 5.90 | membrane protein, palmitoylated | 2.33 | 9.59 | 0.22 | 0.004420 |
| 5.90 | FXYD domain-containing ion transport regulator 2 | 3.44 | 8.42 | 0.07 | 0.000177 |
| 5.90 | coiled-coil domain containing 37 | 2.50 | 9.42 | 0.19 | 0.003133 |
| 5.88 | RIKEN cDNA 9530096D07 gene | 3.12 | 8.71 | 0.11 | 0.000547 |
| 5.87 | general transcription factor II E, polypeptide 2 (beta subunit) | 2.45 | 9.40 | 0.20 | 0.003348 |
| 5.87 | kelch domain containing 1 | 2.32 | 9.53 | 0.22 | 0.004415 |
| 5.82 | similar to cyclin-dependent kinase 2-interacting protein | 3.24 | 8.48 | 0.10 | 0.000328 |
| 5.80 | MORN repeat containing 2 | 2.63 | 9.07 | 0.17 | 0.002053 |
| 5.80 | poly (ADP-ribose) polymerase family, member 8 | 2.55 | 9.15 | 0.18 | 0.002507 |
| 5.79 | nucleoporin like 1 | 2.83 | 8.83 | 0.14 | 0.001176 |
| 5.73 | RIKEN cDNA C330018D20 gene | 3.55 | 7.96 | 0.05 | 0.000067 |
| 5.73 | RIKEN cDNA B230311B06 gene | 2.21 | 9.36 | 0.23 | 0.004915 |
| 5.72 | translocase of outer mitochondrial membrane 40 homolog-like (yeast) | 2.54 | 9.01 | 0.18 | 0.002394 |
| 5.70 | acid phosphatase 6, lysophosphatidic | 2.28 | 9.24 | 0.21 | 0.004208 |
| 5.70 | RIKEN cDNA 4833408A19 gene | 3.48 | 7.97 | 0.06 | 0.000087 |
| 5.68 | KN motif and ankyrin repeat domains 3 | 2.44 | 9.02 | 0.19 | 0.002873 |
| 5.67 | neighbor of Brca1 gene 1 | 3.00 | 8.41 | 0.11 | 0.000576 |
| 5.67 | insulin-like growth factor binding protein-like 1 | 4.12 | 7.24 | 0.02 | 0.000001 |
| 5.65 | inhibitor of kappaB kinase gamma | 2.60 | 8.80 | 0.17 | 0.001894 |
| 5.64 | C-type lectin domain family 11, member a | 2.46 | 8.93 | 0.18 | 0.002669 |
| 5.63 | predicted gene 7684; membrane-associated ring finger (C3HC4) 5 | 2.62 | 8.73 | 0.16 | 0.001744 |
| 5.62 | DnaJ-like protein | 2.38 | 8.98 | 0.19 | 0.003168 |
| 5.62 | lymphoid-restricted membrane protein | 2.81 | 8.50 | 0.13 | 0.000972 |
| 5.61 | transmembrane 9 superfamily member 2 | 2.92 | 8.37 | 0.12 | 0.000678 |
| 5.61 | MAK10 homolog, amino-acid N-acetyltransferase subunit, (S. cerevisiae) | 2.28 | 9.04 | 0.21 | 0.003851 |
| 5.59 | protocadherin beta 2 | 3.13 | 8.10 | 0.09 | 0.000293 |
| 5.58 | RIKEN cDNA M5C1000I18 gene | 2.64 | 8.61 | 0.16 | 0.001552 |
| 5.52 | histone cluster 2, H2be | 2.18 | 8.97 | 0.22 | 0.004446 |
| 5.48 | predicted gene 8717; adaptor protein complex AP-2, mu1 | 2.39 | 8.66 | 0.18 | 0.002632 |
| 5.46 | transmembrane protein 63c | 3.26 | 7.71 | 0.07 | 0.000124 |
| 5.45 | peroxisomal biogenesis factor 3 | 2.41 | 8.57 | 0.18 | 0.002391 |
| 5.44 | alanyl-tRNA synthetase domain containing 1; RIKEN cDNA 1700113I22 gene | 3.24 | 7.69 | 0.07 | 0.000130 |
| 5.42 | predicted gene 7743; calmodulin 3; calmodulin 2; calmodulin 1; predicted gene 7308 | 2.41 | 8.51 | 0.18 | 0.002336 |
| 5.37 | ubiquitin specific peptidase 15 | 2.14 | 8.70 | 0.21 | 0.004248 |
| 5.36 | protocadherin beta 14 | 3.03 | 7.74 | 0.09 | 0.000265 |
| 5.35 | mitogen-activated protein kinase associated protein 1 | 2.20 | 8.59 | 0.20 | 0.003630 |
| 5.33 | predicted gene 5446; similar to FUS interacting protein (serine-arginine rich) 1 | 2.57 | 8.16 | 0.15 | 0.001337 |
| 5.29 | protein phosphatase 2 (formerly 2A), catalytic subunit, alpha isoform | 2.41 | 8.24 | 0.17 | 0.001988 |
| 5.14 | NAD kinase | 2.16 | 8.21 | 0.20 | 0.003315 |
| 5.12 | hypothetical protein LOC100047911 | 2.04 | 8.29 | 0.21 | 0.004320 |
| 5.12 | cyclin D binding myb-like transcription factor 1 | 2.78 | 7.51 | 0.11 | 0.000434 |
| 5.11 | cytochrome c, somatic | 2.26 | 8.03 | 0.18 | 0.002402 |
| 5.10 | predicted gene 318; predicted gene 4776 | 2.66 | 7.61 | 0.12 | 0.000681 |
| 5.10 | IMP4, U3 small nucleolar ribonucleoprotein, homolog (yeast) | 2.17 | 8.12 | 0.19 | 0.003115 |
| 5.10 | transmembrane protein 107 | 2.17 | 8.12 | 0.19 | 0.003090 |
| 5.10 | proline rich 8 | 1.97 | 8.32 | 0.23 | 0.004996 |
| 5.09 | transmembrane protein 41B | 2.29 | 7.96 | 0.18 | 0.002207 |
| 5.08 | predicted gene 12500 | 2.26 | 7.99 | 0.18 | 0.002375 |
| 5.05 | E2F transcription factor 4 | 2.60 | 7.55 | 0.13 | 0.000743 |
| 5.03 | cell adhesion molecule 2 | 2.20 | 7.94 | 0.18 | 0.002619 |
| 5.03 | mitochondrial ribosomal protein L15 | 2.50 | 7.61 | 0.13 | 0.001020 |
| 5.01 | testis specific 10 | 2.00 | 8.11 | 0.21 | 0.004264 |
| 4.98 | cystatin 12 | 2.66 | 7.35 | 0.11 | 0.000531 |
| 4.93 | predicted gene 9971 | 2.16 | 7.76 | 0.18 | 0.002566 |
| 4.90 | ubiquitin specific peptidase 16 | 1.98 | 7.91 | 0.21 | 0.004081 |
| 4.87 | pyruvate carboxylase | 1.98 | 7.85 | 0.21 | 0.003970 |
| 4.87 | RIKEN cDNA 2010002N04 gene | 2.25 | 7.55 | 0.17 | 0.001819 |
| 4.83 | protocadherin beta 12 | 2.58 | 7.13 | 0.11 | 0.000529 |
| 4.83 | NFKB inhibitor interacting Ras-like protein 1 | 2.56 | 7.15 | 0.11 | 0.000579 |
| 4.82 | reactive oxygen species modulator 1 | 2.26 | 7.45 | 0.16 | 0.001671 |
| 4.80 | bromodomain, testis-specific | 2.42 | 7.23 | 0.13 | 0.000925 |
| 4.77 | predicted gene 5626; stomatin (Epb7.2)-like 2 | 2.25 | 7.35 | 0.16 | 0.001608 |
| 4.77 | coiled-coil-helix-coiled-coil-helix domain containing 8 | 2.21 | 7.38 | 0.16 | 0.001784 |
| 4.76 | platelet-activating factor acetylhydrolase, isoform 1b, subunit 1 | 2.02 | 7.57 | 0.19 | 0.003104 |
| 4.76 | mitochondrial ribosomal protein L51 | 2.39 | 7.19 | 0.13 | 0.000965 |
| 4.75 | progesterone immunomodulatory binding factor 1 | 2.08 | 7.50 | 0.18 | 0.002615 |
| 4.74 | predicted gene 715 | 2.35 | 7.18 | 0.14 | 0.001079 |
| 4.73 | polymerase (RNA) III (DNA directed) polypeptide K | 1.87 | 7.68 | 0.22 | 0.004478 |
| 4.72 | G-protein signalling modulator 2 (AGS3-like, C. elegans) | 2.20 | 7.31 | 0.16 | 0.001733 |
| 4.72 | similar to vacuolar protein sorting 25; vacuolar protein sorting 25 (yeast) | 1.97 | 7.55 | 0.20 | 0.003473 |
| 4.71 | COBW domain containing 1 | 1.87 | 7.62 | 0.22 | 0.004377 |
| 4.66 | transmembrane protein 135 | 2.12 | 7.27 | 0.17 | 0.002074 |
| 4.57 | histone cluster 2, H2ab | 2.15 | 7.06 | 0.16 | 0.001659 |
| 4.57 | predicted gene 4995 | 2.20 | 7.00 | 0.15 | 0.001398 |
| 4.57 | family with sequence similarity 171, member A2 | 1.84 | 7.38 | 0.21 | 0.004169 |
| 4.57 | coiled-coil domain containing 132 | 1.86 | 7.35 | 0.21 | 0.003877 |
| 4.57 | helicase-like transcription factor | 2.39 | 6.79 | 0.12 | 0.000664 |
| 4.53 | WAP four-disulfide core domain 3 | 2.24 | 6.86 | 0.14 | 0.001081 |
| 4.50 | WD repeat domain 92 | 2.38 | 6.67 | 0.12 | 0.000598 |
| 4.49 | mitogen-activated protein kinase 9 | 2.30 | 6.73 | 0.13 | 0.000814 |
| 4.46 | RIKEN cDNA 4930455F23 gene | 1.89 | 7.10 | 0.19 | 0.003203 |
| 4.46 | galactosylceramidase | 1.92 | 7.05 | 0.19 | 0.002845 |
| 4.45 | RIKEN cDNA 8430426J06 gene | 2.28 | 6.66 | 0.13 | 0.000805 |
| 4.44 | GC-rich promoter binding protein 1 | 1.99 | 6.95 | 0.18 | 0.002254 |
| 4.41 | adaptor-related protein complex AP-4, mu 1 | 2.07 | 6.80 | 0.16 | 0.001655 |
| 4.39 | taurine upregulated gene 1 | 1.75 | 7.11 | 0.22 | 0.004412 |
| 4.34 | Ras and Rab interactor-like | 1.92 | 6.82 | 0.18 | 0.002457 |
| 4.28 | predicted gene 9897 | 2.09 | 6.52 | 0.15 | 0.001238 |
| 4.28 | spastin | 2.44 | 6.15 | 0.08 | 0.000243 |
| 4.25 | ubiquitin protein ligase E3A | 2.33 | 6.21 | 0.10 | 0.000405 |
| 4.23 | helicase-like transcription factor | 1.76 | 6.75 | 0.20 | 0.003493 |
| 4.12 | predicted gene 2785 | 1.70 | 6.60 | 0.20 | 0.003682 |
| 4.10 | membrane bound O-acyltransferase domain containing 2 | 1.60 | 6.66 | 0.22 | 0.004863 |
| 4.07 | DOT1-like, histone H3 methyltransferase (S. cerevisiae) | 1.84 | 6.35 | 0.17 | 0.002135 |
| 4.06 | ubiquitin specific peptidase 34 | 2.23 | 5.92 | 0.10 | 0.000391 |
| 4.03 | integrator complex subunit 3 | 1.64 | 6.48 | 0.21 | 0.003931 |
| 4.02 | sterile alpha motif domain containing 12 | 1.87 | 6.22 | 0.17 | 0.001801 |
| 4.00 | RIKEN cDNA 4930407I10 gene | 1.96 | 6.09 | 0.15 | 0.001229 |
| 4.00 | profilin 2 | 1.59 | 6.46 | 0.22 | 0.004470 |
| 3.99 | high mobility group box transcription factor 1 | 2.05 | 5.96 | 0.13 | 0.000778 |
| 3.98 | ligase III, DNA, ATP-dependent | 1.65 | 6.38 | 0.20 | 0.003648 |
| 3.97 | RIKEN cDNA 2810432L12 gene | 1.70 | 6.29 | 0.19 | 0.002968 |
| 3.94 | BCL2-associated athanogene 2 | 1.79 | 6.14 | 0.17 | 0.002075 |
| 3.91 | F-box and WD-40 domain protein 4 | 1.89 | 5.96 | 0.15 | 0.001324 |
| 3.90 | nuclear receptor subfamily 1, group H, member 5 | 2.14 | 5.69 | 0.10 | 0.000404 |
| 3.88 | signal transducer and activator of transcription 6 | 1.65 | 6.16 | 0.19 | 0.003156 |
| 3.87 | pleckstrin homology-like domain, family B, member 1 | 2.06 | 5.72 | 0.11 | 0.000561 |
| 3.84 | uronyl-2-sulfotransferase | 1.53 | 6.20 | 0.22 | 0.004356 |
| 3.84 | cell division cycle associated 8 | 1.49 | 6.24 | 0.23 | 0.004954 |
| 3.80 | DnaJ (Hsp40) homolog, subfamily B, member 3 | 1.80 | 5.84 | 0.16 | 0.001568 |
| 3.77 | golgi transport 1 homolog B (S. cerevisiae) | 1.57 | 6.02 | 0.20 | 0.003531 |
| 3.71 | tubulin, delta 1 | 1.49 | 5.98 | 0.21 | 0.004232 |
| 3.63 | aminoadipate-semialdehyde synthase | 1.50 | 5.81 | 0.20 | 0.003687 |
| 3.62 | predicted gene 14127; RIKEN cDNA 1110005A03 gene | 1.86 | 5.42 | 0.13 | 0.000825 |
| 3.62 | predicted gene 5434; ubiquitin-conjugating enzyme E2F (putative) | 1.47 | 5.82 | 0.21 | 0.004025 |
| 3.59 | sciellin | 1.54 | 5.69 | 0.19 | 0.003043 |
| 3.56 | potassium channel, subfamily K, member 15 | 2.08 | 5.05 | 0.07 | 0.000175 |
| 3.55 | predicted gene 5465 | 1.40 | 5.75 | 0.22 | 0.004619 |
| 3.44 | potassium voltage-gated channel, subfamily Q, member 3 | 1.60 | 5.31 | 0.17 | 0.001808 |
| 3.43 | ribosomal protein L6 | 1.69 | 5.19 | 0.14 | 0.001134 |
| 3.41 | RIKEN cDNA 2310075K07 gene | 1.35 | 5.52 | 0.22 | 0.004560 |
| 3.41 | copine V; similar to Copine V | 1.42 | 5.44 | 0.20 | 0.003566 |
| 3.34 | p21 protein (Cdc42/Rac)-activated kinase 2 | 1.52 | 5.19 | 0.17 | 0.002108 |
| 3.30 | vesicle transport through interaction with t-SNAREs homolog 1A (yeast) | 1.31 | 5.33 | 0.22 | 0.004547 |
| 3.29 | chromodomain helicase DNA binding protein 6; predicted gene 8291 | 1.36 | 5.25 | 0.20 | 0.003729 |
| 3.28 | cysteine-rich perinuclear theca 8 | 1.43 | 5.16 | 0.18 | 0.002694 |
| 3.26 | mitochondrial ribosomal protein L45 | 1.67 | 4.87 | 0.13 | 0.000801 |
| 3.23 | adducin 2 (beta) | 1.57 | 4.92 | 0.15 | 0.001304 |
| 3.23 | UDP-Gal:betaGlcNAc beta 1,3-galactosyltransferase, polypeptide 1 | 1.41 | 5.08 | 0.18 | 0.002713 |
| 3.22 | sulfotransferase family 5A, member 1 | 1.43 | 5.06 | 0.18 | 0.002526 |
| 3.22 | WAP four-disulfide core domain 5 | 1.30 | 5.17 | 0.21 | 0.004065 |
| 3.20 | HIG1 domain family, member 1B | 1.28 | 5.16 | 0.22 | 0.004398 |
| 3.19 | LIM and senescent cell antigen-like domains 1 | 1.43 | 4.98 | 0.18 | 0.002270 |
| 3.18 | heparanase | 1.36 | 5.03 | 0.19 | 0.003008 |
| 3.16 | serine incorporator 5 | 1.36 | 5.00 | 0.19 | 0.002968 |
| 3.16 | G two S phase expressed protein 1 | 1.41 | 4.94 | 0.18 | 0.002341 |
| 3.15 | Mitogen-activated protein kinase kinase 1 interacting protein 1 (MEK binding partner 1) (Mp1) | 1.75 | 4.56 | 0.10 | 0.000351 |
| 3.15 | protein kinase C and casein kinase substrate in neurons 2 | 1.31 | 5.01 | 0.20 | 0.003507 |
| 3.14 | RPA interacting protein | 1.41 | 4.90 | 0.18 | 0.002334 |
| 3.13 | protein kinase, cGMP-dependent, type I | 1.32 | 4.98 | 0.20 | 0.003366 |
| 3.13 | predicted gene 10190 | 1.63 | 4.64 | 0.12 | 0.000689 |
| 3.11 | testis serine protease 3 | 1.41 | 4.85 | 0.18 | 0.002209 |
| 3.11 | predicted gene 5157; similar to serine/threonine kinase | 1.74 | 4.51 | 0.10 | 0.000335 |
| 3.07 | phosphatidylserine synthase 2 | 1.28 | 4.90 | 0.20 | 0.003532 |
| 3.06 | LIM domain only 1 | 1.19 | 4.96 | 0.23 | 0.004974 |
| 3.01 | CD86 antigen | 1.26 | 4.78 | 0.20 | 0.003403 |
| 2.96 | vomeronasal 1 receptor, C26 | 1.45 | 4.49 | 0.15 | 0.001208 |
| 2.96 | RIKEN cDNA D630042P16 gene | 1.23 | 4.71 | 0.20 | 0.003582 |
| 2.95 | ubiquitin specific petidase 45 | 1.24 | 4.69 | 0.20 | 0.003416 |
| 2.93 | leucyl/cystinyl aminopeptidase | 1.34 | 4.55 | 0.17 | 0.002064 |
| 2.87 | major urinary protein 1 | 1.41 | 4.34 | 0.14 | 0.001178 |
| 2.86 | ATP-binding cassette, sub-family B (MDR/TAP), member 5 | 1.16 | 4.60 | 0.21 | 0.004085 |
| 2.79 | predicted gene 15386; defensin beta 41 | 1.17 | 4.43 | 0.20 | 0.003462 |
| 2.77 | olfactory receptor 836 | 1.16 | 4.40 | 0.20 | 0.003510 |
| 2.75 | protocadherin beta 21 | 1.16 | 4.38 | 0.20 | 0.003426 |
| 2.73 | monoacylglycerol O-acyltransferase 2 | 1.08 | 4.41 | 0.22 | 0.004597 |
| 2.73 | similar to vomeronasal 1 receptor, D8; vomeronasal 1 receptor, D2; p | 1.19 | 4.29 | 0.18 | 0.002728 |
| 2.72 | serine (or cysteine) peptidase inhibitor, clade C (antithrombin), member 1 | 1.19 | 4.27 | 0.18 | 0.002651 |
| 2.66 | protamine 1 | 1.12 | 4.22 | 0.20 | 0.003405 |
| 2.66 | RIKEN cDNA C230094A16 gene | 1.11 | 4.22 | 0.20 | 0.003453 |
| 2.63 | FIP1 like 1 (S. cerevisiae) | 1.24 | 4.04 | 0.16 | 0.001671 |
| 2.57 | transmembrane protein 69 | 1.25 | 3.90 | 0.15 | 0.001285 |
| 2.56 | isoprenylcysteine carboxyl methyltransferase | 1.14 | 4.00 | 0.18 | 0.002478 |
| 2.56 | late cornified envelope 1L | 1.17 | 3.97 | 0.17 | 0.002094 |
| 2.53 | caldesmon 1 | 0.99 | 4.09 | 0.23 | 0.004891 |
| 2.47 | amine oxidase, copper containing 2 (retina-specific) | 1.14 | 3.82 | 0.17 | 0.001896 |
| 2.46 | cDNA sequence BC049349 | 1.10 | 3.84 | 0.18 | 0.002367 |
| 2.46 | EH-domain containing 2 | 1.28 | 3.66 | 0.13 | 0.000749 |
| 2.45 | interleukin 6 signal transducer | 1.07 | 3.86 | 0.19 | 0.002826 |
| 2.44 | ATPase, class VI, type 11B | 1.16 | 3.74 | 0.16 | 0.001548 |
| 2.43 | splicing factor, arginine/serine-rich 12 | 1.07 | 3.81 | 0.18 | 0.002650 |
| 2.41 | peptidylprolyl isomerase (cyclophilin)-like 3 | 1.08 | 3.76 | 0.18 | 0.002371 |
| 2.40 | olfactory receptor 474 | 1.05 | 3.78 | 0.19 | 0.002819 |
| 2.32 | mitochondrial ribosomal protein L20 | 1.08 | 3.59 | 0.17 | 0.001902 |
| 2.30 | vomeronasal 2, receptor, 43 | 1.36 | 3.25 | 0.07 | 0.000153 |
| 2.29 | platelet-derived growth factor, C polypeptide | 1.16 | 3.43 | 0.13 | 0.000919 |
| 2.28 | growth factor receptor bound protein 2; predicted gene 12791 | 0.99 | 3.59 | 0.19 | 0.002937 |
| 2.26 | olfactory receptor 411 | 0.90 | 3.63 | 0.22 | 0.004373 |
| 2.19 | slit homolog 2 (Drosophila) | 0.88 | 3.53 | 0.22 | 0.004443 |
| 2.16 | predicted gene 8160 | 1.13 | 3.19 | 0.12 | 0.000674 |
| 2.15 | inositol 1,4,5-triphosphate receptor interacting protein-like 1 | 0.96 | 3.36 | 0.18 | 0.002431 |
| 2.15 | predicted gene 4461; olfactory receptor 239; olfactory receptor 55 | 0.88 | 3.43 | 0.21 | 0.003879 |
| 2.11 | predicted gene 8640 | 0.89 | 3.35 | 0.20 | 0.003466 |
| 1.91 | gametocyte specific factor 1 | 0.78 | 3.06 | 0.21 | 0.003981 |
| 1.85 | similar to TRAV8D-2; predicted gene, EG667604 | 0.95 | 2.76 | 0.13 | 0.000804 |
| 1.74 | G protein-coupled receptor 132 | 0.72 | 2.76 | 0.20 | 0.003620 |
| 1.69 | predicted gene 4869 | 0.79 | 2.60 | 0.17 | 0.001863 |
| 1.66 | tyrosyl-tRNA synthetase 2 (mitochondrial) | 0.66 | 2.68 | 0.22 | 0.004710 |
| 1.52 | similar to zinc finger protein 420 | 0.60 | 2.44 | 0.22 | 0.004751 |
| 1.33 | mixed lineage kinase domain-like | 0.56 | 2.10 | 0.20 | 0.003446 |
